# Supplementary material for: McClintock: An Integrated Pipeline for Detecting Transposable Element Insertions in Whole-Genome Shotgun Sequencing Data
Source: G3 (Bethesda). 2017 Jun 21;7(8):2763–78. doi: 10.1534/g3.117.043893 (PMC5555480; doi:10.1534/g3.117.043893)
Supplement: Supplementary file 1 [file 2763FileS1.pdf]

McClintock: An integrated pipeline for detecting transposable element insertions in whole genome shotgun sequencing data.

Michael G. Nelson[*], Raquel S. Linheiro[*,†], and Casey M. Bergman[*,‡]

[*]Faculty of Life Sciences, University of Manchester, Oxford Road, Manchester, UK
[†]Current Address: Centro de Biotecnologia e Química Fina (CBQF), Universidade Católica Portuguesa, Rua Arquiteto Lobão Vital, Porto, PT
[‡]Current Address: Department of Genetics and Institute of Bioinformatics, University of Georgia, 120 E. Green St., Athens, GA, USA

Address for correspondence:
Casey M. Bergman
Department of Genetics and Institute of Bioinformatics
University of Georgia
Davison Life Sciences Building
120 E. Green St.
Athens, GA 30601
cbergman@uga.edu

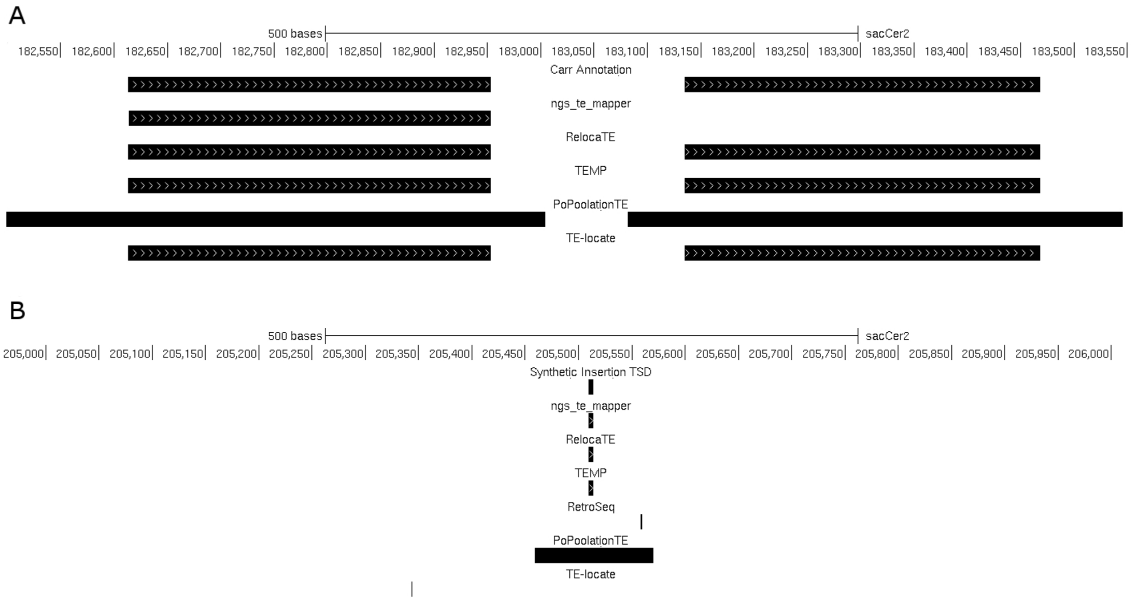

Figure S1. TE annotation frameworks used by McClintock component methods.

A. UCSC genome browser screenshot showing examples of reference TE annotations made by McClintock component methods for a section of ChrI from *S. cerevisiae*. The reference TE annotation from Carr *et al.* (2012) is displayed at the top, and shows two reference TE fragments annotated in this region. Arrowheads denote the direction of the TE insertion, which is provided for all component methods except PoPoolationTE. **B.** UCSC genome browser screenshot showing examples of non-reference TE annotations made by McClintock component methods for a synthetic TE insertion inserted into the *S. cerevisiae* genome. The five bp TSD of the synthetic TE insertion is shown at the top. Arrowheads in the predicted span denote the direction of the non-reference TE insertion, which is provided for all component methods except PoPoolationTE.

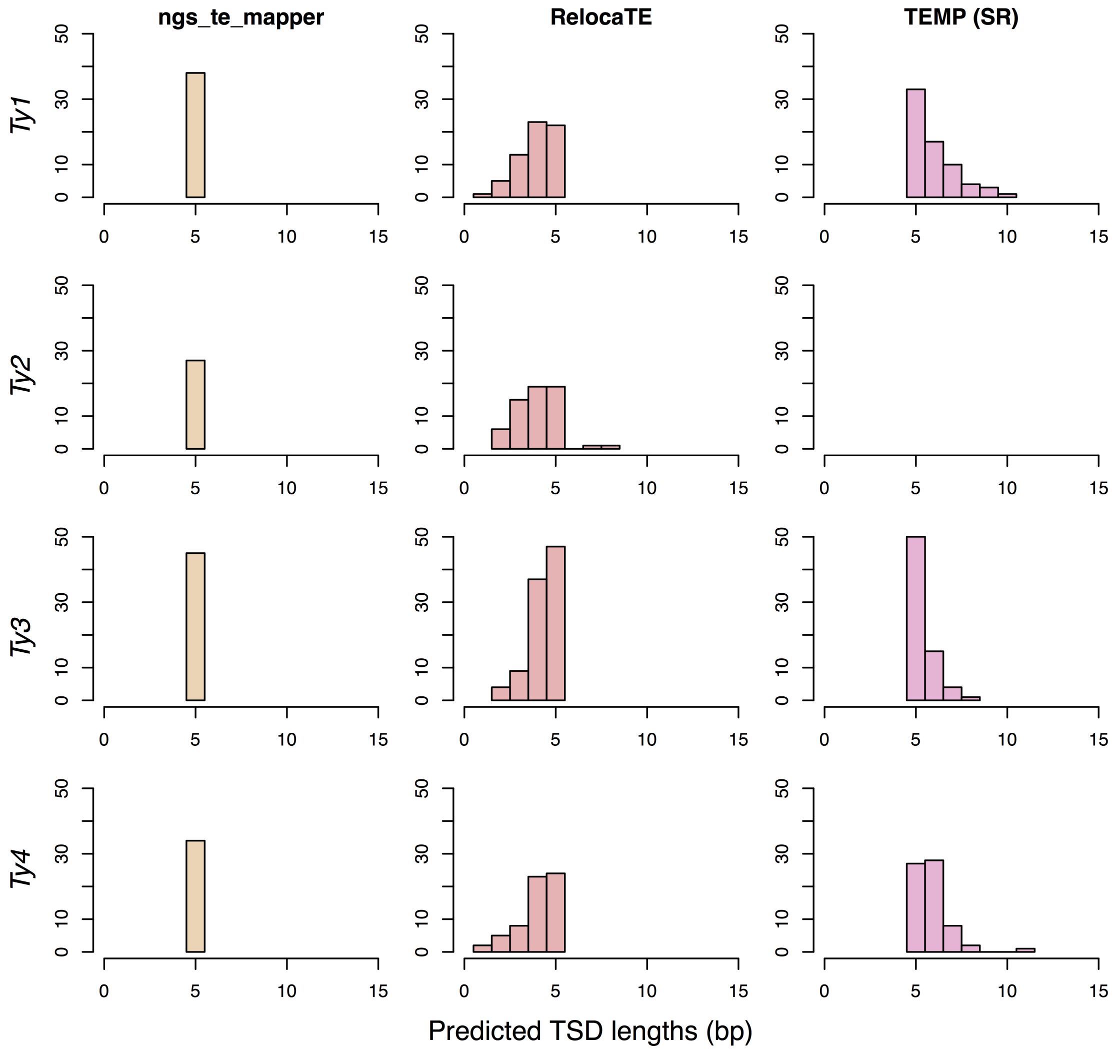

Figure S2. TSD lengths for predicted non-reference TE insertions with split-read evidence in single insertion synthetic genomes.

No insertions were predicted by TEMP for *Ty2* using split-read data.

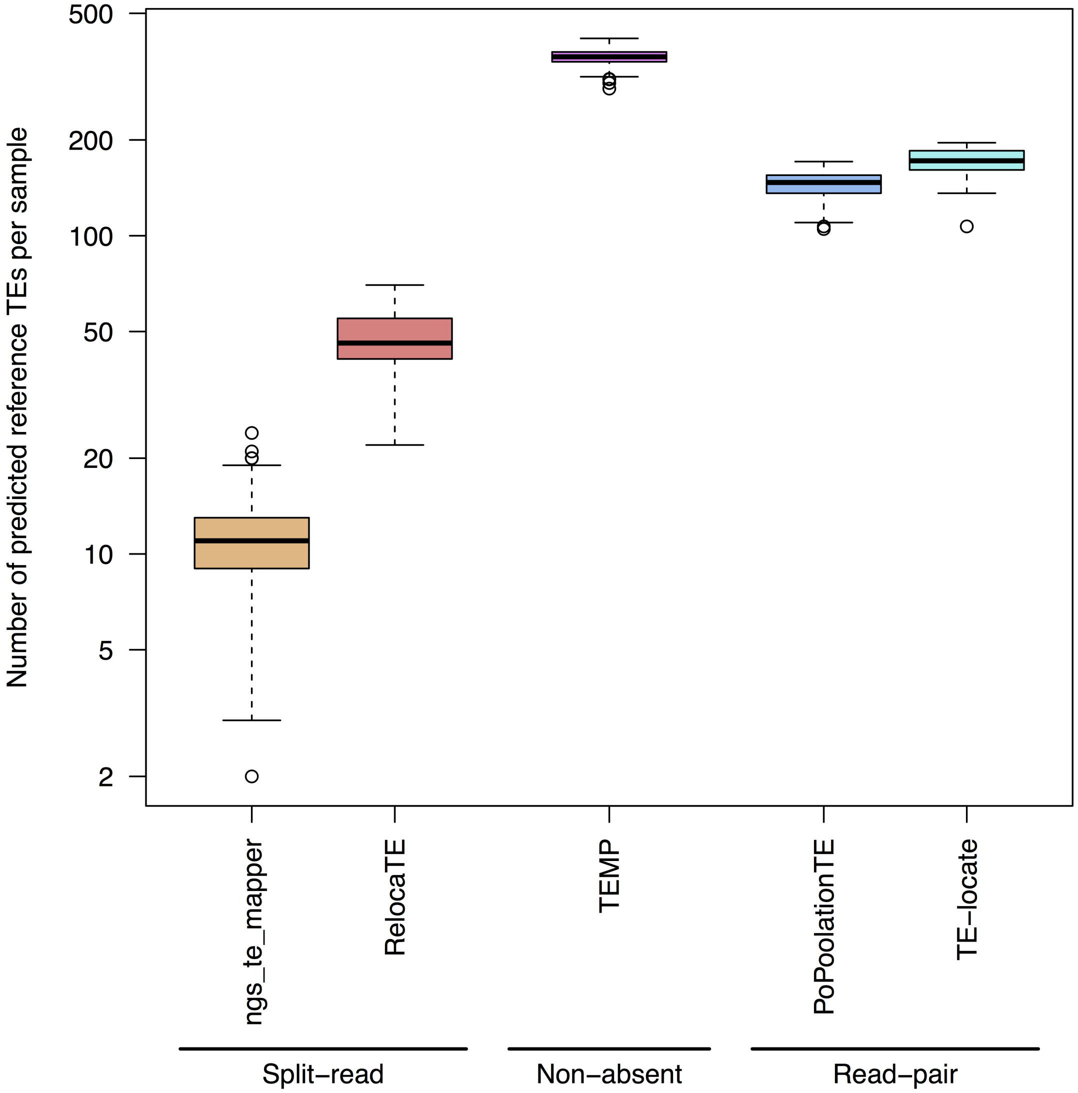

Figure S3. Numbers of reference TE insertions per strain predicted by McClintock component methods in real yeast genomes.

Data are based on 93 yeast strains taken from Strope *et al.* (2015). Predictions for TEMP are based on the no evidence for the absence of a reference TE insertion (non-absent) and are therefore not directly comparable to other split-read or read-pair methods. The box plot is shown on a $\log_{10}$ scale. The thick line indicates the median, the colored box is the interquartile range, the whiskers mark the most extreme data point which is no more than 1.5 times the interquartile range from the box, and the circles are outliers.

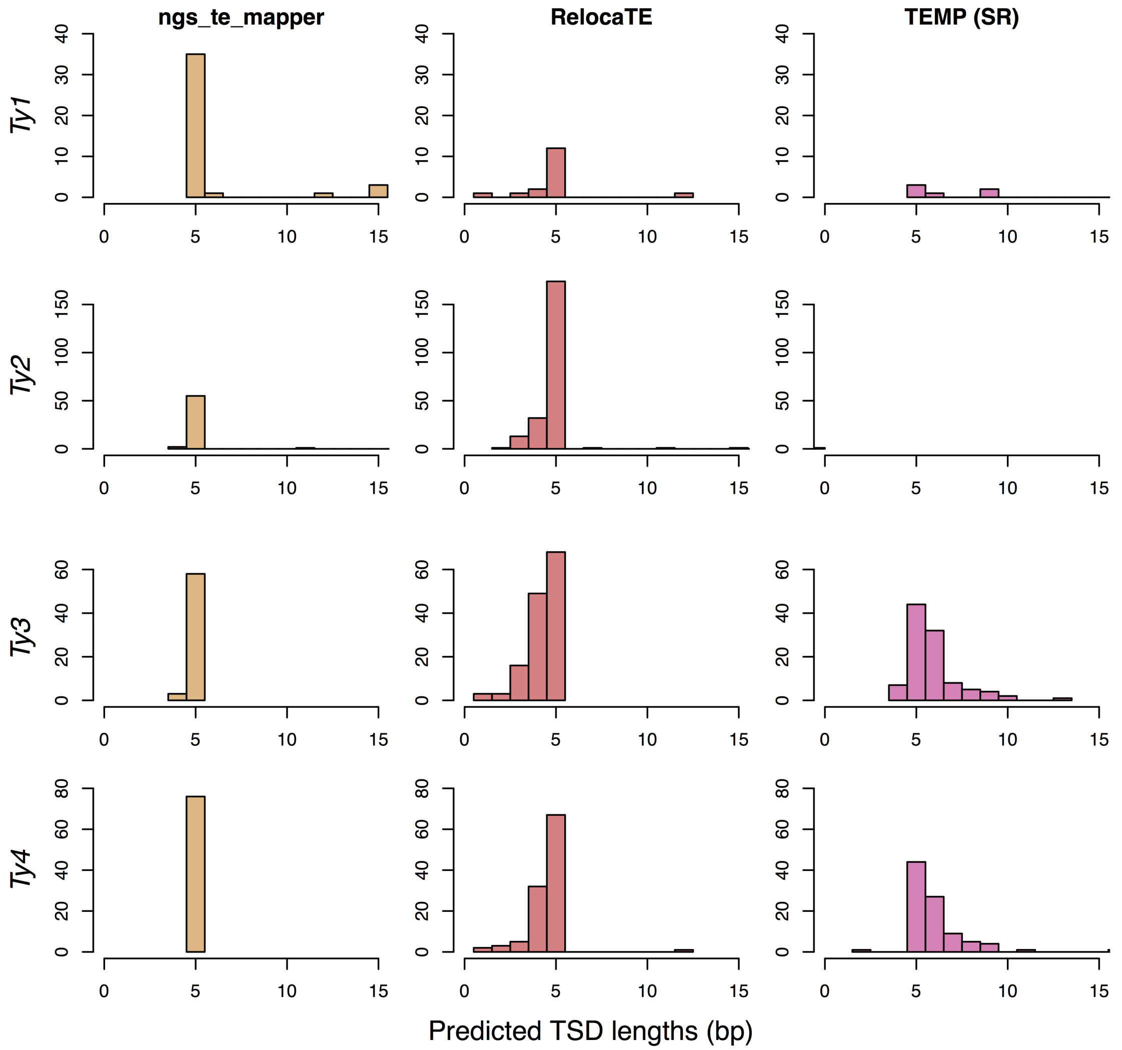

Figure S4. TSD lengths for predicted non-reference TE insertions with split-read evidence in real yeast genomes.

Histograms of TSD lengths for predicted non-reference TE insertions with split-read evidence in real yeast genomes. Data are based on 93 yeast strains taken from Strope *et al.* (2015). No insertions were predicted by TEMP for *Ty2* using split-read data. X-axes were truncated at 15 bp.

Table S1. Numbers of reference TEs predicted by McClintock component methods in simulated resequencing datasets of the reference genome of *S. cerevisiae*. Shown are the average number of non-reference TE predictions (plus or minus the standard deviation) across 100 simulated datasets. WGS shotgun reads were simulated at 10X or 100X coverage, as indicated by the depth column. Carr or RM in the annotation column indicates whether the reference TE annotations were taken from Carr *et al.* (2012) or generated automatically by McClintock using RepeatMasker. A tick in the "Can." column indicates that the canonical TE sequences were added to the reference genome. A tick in the "Ref." column indicates that the TE sequences of reference genome instances were added to the reference genome used for TE detection. Simulated datasets are the same across all reference genome options and do not include these additional TE sequences. RetroSeq is not included in the table as it does not produce predictions for the presence of reference TEs.

Depth	Annotation	Can.	Ref.	ngs_te_mapper	RelocaTE	TEMP[i]	PoPoolationTE[ii]	TE-locate
10X	Carr			21.64 ± 2.50	93.28 ± 3.70	483.00 ± 0.00	80.18 ± 6.78	187.41 ± 5.46
10X	Carr	✔		21.63 ± 2.50	93.28 ± 3.70	483.00 ± 0.00	80.26 ± 6.67	187.41 ± 5.46
10X	Carr		✔	21.54 ± 2.52	80.49 ± 3.42	483.00 ± 0.00	80.00 ± 6.44	187.41 ± 5.46
10X	Carr	✔	✔	21.54 ± 2.52	80.49 ± 3.42	483.00 ± 0.00	80.09 ± 6.39	187.41 ± 5.46
10X	RM			21.64 ± 2.50	99.98 ± 3.77	564.00 ± 0.00	76.43 ± 6.23	182.87 ± 5.14
10X	RM	✔		21.63 ± 2.50	99.98 ± 3.77	564.00 ± 0.00	76.50 ± 6.38	182.87 ± 5.14
10X	RM		✔	21.60 ± 2.52	93.87 ± 3.63	564.00 ± 0.00	76.51 ± 6.24	182.87 ± 5.14
10X	RM	✔	✔	21.59 ± 2.51	93.87 ± 3.63	564.00 ± 0.00	76.39 ± 6.10	182.87 ± 5.14
100X	Carr			40.08 ± 3.35	131.54 ± 2.71	482.99 ± 0.10	164.90 ± 2.95	271.48 ± 2.14
100X	Carr	✔		40.08 ± 3.34	131.54 ± 2.71	482.99 ± 0.10	164.90 ± 2.67	271.48 ± 2.14
100X	Carr		✔	39.76 ± 3.37	114.42 ± 2.86	482.99 ± 0.10	164.84 ± 2.55	271.46 ± 2.14
100X	Carr	✔	✔	39.76 ± 3.37	114.42 ± 2.86	482.99 ± 0.10	164.59 ± 2.96	271.46 ± 2.14
100X	RM			40.08 ± 3.35	132.36 ± 2.88	563.99 ± 0.10	172.83 ± 2.19	272.60 ± 2.10
100X	RM	✔		40.08 ± 3.34	132.36 ± 2.88	563.99 ± 0.10	172.64 ± 2.37	272.60 ± 2.10
100X	RM		✔	39.91 ± 3.33	124.55 ± 3.00	563.99 ± 0.10	172.61 ± 2.14	272.58 ± 2.13
100X	RM	✔	✔	39.91 ± 3.33	124.55 ± 3.00	563.99 ± 0.10	172.66 ± 2.07	272.58 ± 2.13

[i] The TEMP pipeline does not make direct predictions of reference TEs present, these numbers are inferred from there being no evidence of absence for these TEs. [ii] PoPoolationTE produces its own alterations to the reference genome and McClintock does not apply modifications used for other methods to PoPoolationTE. Thus the only differences in input for PoPoolationTE are between the Carr *et al.* (2012) and RepeatMasker reference TE annotations.

Table S2. Numbers of non-reference TEs predicted in 100 simulated resequencing datasets of the reference strain of *S. cerevisiae*. Shown are the mean number of non-reference TE predictions plus or minus the standard deviation. Sequencing was simulated at 10 or 100X, indicated by the depth column. Carr or RM in the annotation column indicates whether the reference TE annotations were from Carr *et al.* (2012) or generated automatically by RepeatMasker. A tick in the “Can.” column indicates that the canonical TE sequences were added to the reference genome used for TE detection. A tick in the “Ref.” column indicates that the TE sequences of reference genome instances were added to the reference genome used for TE detection. Simulated datasets are the same across all reference genome options and do not include these additional TE sequences.

Depth	Annotation	Can.	Ref.	ngs_te_mapper	RelocaTE	TEMP	RetroSeq	PoPoolationTE[i]	TE-locate
10X	Carr			0.00 ± 0.00	0.02 ± 0.14	0.00 ± 0.00	0.00 ± 0.00	2.84 ± 1.25	0.00 ± 0.00
10X	Carr	✔		0.00 ± 0.00	0.02 ± 0.14	0.00 ± 0.00	0.00 ± 0.00	2.72 ± 1.09	0.00 ± 0.00
10X	Carr		✔	0.00 ± 0.00	0.02 ± 0.14	0.00 ± 0.00	0.00 ± 0.00	2.90 ± 1.24	0.00 ± 0.00
10X	Carr	✔	✔	0.00 ± 0.00	0.02 ± 0.14	0.00 ± 0.00	0.00 ± 0.00	2.79 ± 1.15	0.00 ± 0.00
10X	RM			0.00 ± 0.00	0.02 ± 0.14	0.00 ± 0.00	0.00 ± 0.00	1.60 ± 0.92	0.00 ± 0.00
10X	RM	✔		0.00 ± 0.00	0.02 ± 0.14	0.00 ± 0.00	0.00 ± 0.00	1.62 ± 0.90	0.00 ± 0.00
10X	RM		✔	0.00 ± 0.00	0.02 ± 0.14	0.00 ± 0.00	0.00 ± 0.00	1.62 ± 0.92	0.00 ± 0.00
10X	RM	✔	✔	0.00 ± 0.00	0.02 ± 0.14	0.00 ± 0.00	0.00 ± 0.00	1.60 ± 0.91	0.00 ± 0.00
100X	Carr			0.00 ± 0.00	0.16 ± 0.37	0.00 ± 0.00	0.00 ± 0.00	0.95 ± 0.74	0.00 ± 0.00
100X	Carr	✔		0.00 ± 0.00	0.16 ± 0.37	0.00 ± 0.00	0.00 ± 0.00	0.87 ± 0.75	0.00 ± 0.00
100X	Carr		✔	0.00 ± 0.00	0.16 ± 0.37	0.00 ± 0.00	0.00 ± 0.00	0.83 ± 0.73	0.00 ± 0.00
100X	Carr	✔	✔	0.00 ± 0.00	0.16 ± 0.37	0.00 ± 0.00	0.00 ± 0.00	0.90 ± 0.76	0.00 ± 0.00
100X	RM			0.00 ± 0.00	0.17 ± 0.38	0.00 ± 0.00	0.00 ± 0.00	2.01 ± 0.83	0.00 ± 0.00
100X	RM	✔		0.00 ± 0.00	0.17 ± 0.38	0.00 ± 0.00	0.00 ± 0.00	1.99 ± 0.86	0.00 ± 0.00
100X	RM		✔	0.00 ± 0.00	0.17 ± 0.38	0.00 ± 0.00	0.00 ± 0.00	2.09 ± 0.84	0.00 ± 0.00
100X	RM	✔	✔	0.00 ± 0.00	0.17 ± 0.38	0.00 ± 0.00	0.00 ± 0.00	2.04 ± 0.85	0.00 ± 0.00

[i] PoPoolationTE produces its own alterations to the reference genome and McClintock does not apply modifications used for other methods to PoPoolationTE. Thus the only differences in input for PoPoolationTE are between the Carr *et al.* (2012) and RepeatMasker reference TE annotations.

File S1. Supplemental Text.

Description of McClintock Component Methods

ngs_te_mapper

The TE detection pipeline ngs_te_mapper uses split-read evidence to detect both non-reference and reference TE insertions (Linheiro and Bergman, 2012). The original purpose of ngs_te_mapper was to investigate the target site preferences of many LTR-retrotransposon, and DNA transposon families in *D. melanogaster*. Version 79ef861f1d52cdd08eb2d51f145223fad0b2363c of ngs_te_mapper is used by McClintock.

The inputs required to run ngs_te_mapper are fastq sequence reads that can be either single-end or paired-end, a fasta reference genome, and a fasta file of the canonical TE sequences for that organism.

To detect TE insertions, ngs_te_mapper first independently aligns each of the pair of fastq reads against the canonical TE sequences using BWA, ignoring the paired-end information. Reads that uniquely map to the start or end of a canonical TE ("junction reads") are selected and the full read is mapped to the reference genome. If a TE-containing read aligns partially to the reference genome in a unique location then it was retained as support for one end of a non-reference TE insertion. From the set of reads supporting a non-reference TE insertion, clusters of junction reads that align to the same canonical TE and map to the reference genome with an overlap less than or equal to 20 bp were used to define the TSD of that TE insertion. For a non-reference TE insertion to be called, ngs_te_mapper requires at least one junction read from both ends of the TE to be present in each cluster, with the overlap from both ends defining the TSD. Information about which end of the TE, and in which orientation junction reads align to, is used to determine the orientation of non-reference TE insertions in the genome. Because the full read is retained and aligned to the reference genome, if a junction read maps to a location where a TE sequence for the same family is present in the reference genome, it is possible to differentiate reference from non-reference TE insertions. If a junction read aligns fully to the reference genome, instead of partially as mentioned previously, it supports a reference TE sequence in the sample at that location. To call a TE shared with the reference genome, ngs_-

te_mapper requires that at least one read is found to support each end of the reference TE. No reference TE annotation is required for ngs_te_mapper.

The output of ngs_te_mapper is a BED format file containing records for each detected TE. For non-reference TE predictions, the annotation contains the start and end coordinates of the predicted TSD for the insertion, in zero-based coordinates. For reference TEs, the annotation contains the predicted start and end coordinate for the entire TE span in the reference genome in zero-based coordinates. The coordinates of reference TEs output by ngs_te_mapper may differ slightly compared with pre-existing annotation of reference TEs, because no reference TE annotation information is used as input for ngs_te_mapper. Additional information, such as the orientation of the predicted TE, its family, and whether it was detected as a reference or non-reference insertion is saved within the record's name.

The approach used by ngs_te_mapper leads to some limitations, as discussed in (Linheiro and Bergman, 2012). Due to the ngs_te_mapper requiring the ends of the TE sequence to find junction reads, it can only detect TE insertions with intact termini. This means ngs_te_mapper is not effective at detecting non-LTR retrotransposons, which are commonly truncated at the 5' end due to incomplete reverse transcription. For non-reference TE insertions, ngs_te_mapper must also detect a TSD which further limits effectiveness for non-LTR retrotransposons because they do not always produce a TSD on integration and may instead introduce a deletion. Furthermore, since mapping of junction reads to the genome must occur at a unique location, ngs_te_mapper also has limited power to detect reference and non-reference TE insertions in repetitive regions. Finally, ngs_te_mapper can sometimes produce multiple overlapping reference TE predictions at approximately the same location when the boundaries of nearby TEs are uncertain.

No modifications were made to ngs_te_mapper code.

RelocaTE

The TE detection pipeline RelocaTE uses split-read evidence to detect both non-reference and reference TE insertions (Robb *et al.*, 2013). The original purpose of the pipeline was to investigate diversity of a single TE family (*mPing*) in the rice, *Oryza sativa*. RelocaTE version 1.0.5 (commit ce3a2066e15f5c14e2887fdf8dce0485e1750e5b) is used in McClintock.

The inputs required for RelocaTE are fastq sequence reads that can be either single or paired-end, a fasta reference genome, a fasta file of the canonical TE sequences, and a custom formatted annotation file containing the locations of TEs in the reference genome. RelocaTE requires a custom modification of the canonical TE sequence file, namely a comment on the identifier line about the TSD for each TE. For TSDs with known sequence and length this can be provided; for example, “TSD=AGCT”. For TSDs with known length but unknown sequence, the following can be used “TSD=...”, with the number of periods indicating the length. If nothing is known about the TSD for a TE, or it is likely to be highly variable, the user can add “TSD=UNK”. To detect the presence of reference TE copies, a custom formatted TE annotation file is required with the name, start and stop coordinates of each TE in the reference genome in one-based coordinates.

RelocaTE first splits the canonical TE file into separate files, one per TE sequence, and uses BLAT to align the NGS reads to each TE. Reads are then trimmed of TE sequence leaving only unique flanking genomic DNA. RelocaTE then uses bowtie to align trimmed reads to the reference genome. As mentioned previously, paired-end reads are not required, but the paired-end information can be used by RelocaTE to resolve mapping of trimmed reads in repetitive regions. Once reads are uniquely mapped to the genome, overlaps between mapped reads on genomic coordinates indicate the location of the TSD for non-reference TE insertions. At least one read from either side of an insertion giving a perfect overlap to form a TSD is required to call a non-reference TE insertion. The orientation of an insertion is determined from the relative mapping of the TSD and flanking portions of reads, with the predicted orientation based on either side of the TSD needing to agree to accept a prediction. To identify reference TE sequences present in the sample, RelocaTE compares locations of TEs in a reference genome annotation file to the TE-trimmed reads aligned to the reference genome. Unlike with non-reference TEs, because RelocaTE has prior information about reference TEs, it requires only one read at one end of a reference TE for it to be defined as shared with the sample.

The main output of RelocaTE consists of a GFF formatted file in one-based coordinates, one for each TE family supplied as input. The GFF file generated for each TE family provides annotation of the TSD for non-reference insertions. For reference TE sequences, all TE annotations given as input are output, even if they were not detected in the sample. A note in a custom format in column nine of the GFF file distinguishes those reference TEs that were detected as shared with the sample from those that are only present in the reference.

To predict a non-reference TE, RelocaTE must detect an overlap of TE supporting reads with respect to the reference genome, indicating a TSD. As with ngs_te_mapper, this requirement limits the ability to detect non-LTR retrotransposons, because they do not always produce a TSD on integration and may instead introduce a deletion. Since mapping to the genome must occur at a unique location RelocaTE also has limited power to detect non-reference insertions or make accurate reference insertion judgements in repetitive regions. Alignment of NGS reads is performed against individual canonical TE sequences so there is the potential that redundant predictions could be created for TE families that have similar sequences. The process of RelocaTE also includes a computationally expensive alignment using BLAT for each canonical TE sequence individually, meaning that compute time increases quickly with the number of canonical TEs. This is not a problem for the original application on a single TE family or for species like *S. cerevisiae* with only six TE families, but becomes problematic for species like *D. melanogaster* with over 100 TE families. When a sample is predicted to share a reference TE, the orientation of the reference TE is not annotated in the RelocaTE output, although this can be obtained from the original reference TE annotation.

During McClintock installation, a patch is applied to the RelocaTE method that fixes cases where non-reference predictions are identified correctly but are annotated in the wrong location (i.e. the TSD sequence is identified correctly, but the sequence of the reference genome for the coordinates given does not match the reported TSD sequence).

TEMP

TEMP uses both split-read and read-pair evidence to detect non-reference TE insertions and infer the absence of reference TE insertions (Zhuang *et al.*, 2014). The original purpose for TEMP was to analyse TE polymorphism in populations and strains of *D. melanogaster*. TEMP version 1.03 (commit d2500b904e2020d6a1075347b398525ede5feae1) is used by McClintock.

The inputs required for TEMP are a sorted and indexed BAM file of paired-end reads aligned to a reference genome, the fasta reference genome which the short-read alignment was performed against, a fasta file of the canonical TE sequences, annotated locations of TEs in the reference genome in zero-based BED format, and a “hierarchy” file with the first column containing the name of each TE in the reference TE BED file and the second column containing the TE family it belongs to.

To identify non-reference TE insertions, TEMP first identifies discordant paired-end reads in the BAM file that have one uniquely mapped read and one read that is unmapped, or maps to multiple distant locations. The non-uniquely mapped reads from such discordantly mapped pairs are then mapped to the canonical TE sequence file to determine the TE family that is predicted to be present near to the location of the uniquely mapped read. The orientation of the insertion is determined from the orientation of the two reads relative to the canonical TE sequence. From these discordant reads, TEMP can estimate the location of a non-reference TE as being between the end of the uniquely mapped reads plus some additional distance that is related to the average insert size of the sequencing library. By clustering discordant paired-end reads that support the same TE insertion in the same orientation, the estimated interval can be refined. To refine the location of the insertion site further, TEMP identifies soft-clipped (junction) reads from the BAM file that are located in the estimated insertion interval (extended in each direction by 20 bp). If the portion of the clipped read that aligns to the TE is longer than seven bp and maps perfectly to the predicted TE in the correct orientation, then this split read information is used to support an exact junction for the TE.

TEMP can also make predictions for reference TEs that are absent from a resequenced sample. (It is important to emphasise that TEMP does not report direct evidence of whether a reference TE is present in the sample.) To detect the absence of reference TEs, TEMP identifies uniquely mapped paired-end reads that have a longer than average insert size. If these reads span an annotated reference TE location and the insert size becomes the expected length when the reference TE length is removed, then these reads are used as evidence of the absence of the reference TE. Pairs of reads supporting the same absence event are clustered to provide more evidence for the absence call. In the same way as detecting non-reference TE insertions, soft-clipped reads are identified to detect single reads that contain genomic sequence from both sides of the predicted absent TE allowing the pre-TSD to be annotated.

The output of the TEMP non-reference insertion detection module is a custom format file that contains one line for each prediction in one-based coordinates. The information provided includes the estimated interval and whether it is supported by one read only, multiple reads at the same end of the TE, or at least one supporting read at each end of the TE. In addition, the file reports whether an exact junction was detected using split-read information from either or both sides of the TE in separate columns. In the case of a non-reference insertion being supported at only one end of the TE, a single base pair location is annotated. If the junction is detected from both sides, then two positions are reported, and this span can, but not always, represent a

TSD. The output from the reference TE absence module is a custom format file that provides the coordinates of the TE predicted to be absent and the ID of the TE from the reference TE annotation. In addition, if the results are of high quality, then the pre-TSD of the annotated reference TE will be annotated at either end of the TE sequence predicted to be absent.

In cases where TEMP has not detected reads that span the junction at either or both ends of a TE, the location of a TE insertion is an interval estimated from supporting discordant read pairs. As such, not all predictions produced by TEMP are at base level accuracy. Additionally, since mapping to the genome must occur at a unique location, TEMP has limited power to detect non-reference TEs in repetitive regions. As noted above, TEMP does not detect positive evidence of TEs that are shared with the reference genome, but instead detects evidence for the absence of reference TEs. This means assumptions must be made by the user about whether reference TEs that are not reported as absent are present in the sample or there is simply no information to support their absence.

In the absence detection module, TEMP occasionally produces an intermediate BED file that was malformed, with the start coordinate greater than the end coordinate. To prevent the module failing from this error, a patch is applied to TEMP during McClintock installation that removes any malformed BED entries.

RetroSeq

RetroSeq uses read-pair evidence to detect non-reference TE insertions (Nellaker *et al.*, 2012; Keane *et al.*, 2013). The original applications of RetroSeq were to analyse selection on TEs in laboratory mouse strains and to make TE calls in human resequencing data. RetroSeq version 700d4f76a3b996686652866f2b81fefc6f0241e0 is used by McClintock.

The inputs required to run RetroSeq are a BAM file of paired-end reads aligned to a reference genome, the fasta reference genome the alignment was performed against, individual fasta files for each of the canonical TE sequences, plus a file-of-files listing the location of each of the TE fasta files. Alternatively, a BED file with a genomic location of instances for each TE family, plus a file-of-files listing the location of the BED files for each TE family can be supplied instead of canonical TE sequences.

RetroSeq detects novel TE insertions by identifying discordant read pairs in the BAM file. When one end of a paired-end fragment maps uniquely to the genome and the other end either does not map, or maps to a distant location, these read-pairs are retained. The non-mapping or distant mapping reads are then aligned to the canonical TE sequences to identify read-pairs that support a TE insertion. RetroSeq requires at least ten read-pairs to support an insertion at this stage, which are first clustered on each side of the putative TE insertion and then combined to produce an initial window of approximately one to two kilobase pairs (kb) resolution. Within these initial regions RetroSeq attempts to predict potential TE insertion coordinates by scanning from 5' to 3' to determine the point at which the cumulative total of discordant reads supporting either end of the TE is maximised. RetroSeq does not predict whether or not a reference TE sequence is present in a resequenced sample.

The output of RetroSeq is in Variant Call Format (VCF). This file format lists a single base pair genomic location in one-based coordinates and reports what the "variant" is in the sample, in this case, a TE insertion. The output also contains a two base pair interval in the information column that starts with the single coordinate from the position column and ends with that coordinate plus one. This annotation framework implies that the insertion occurs after the single annotated base, in between the two bases listed in the information column. The final column includes the quality of the call and how many of the filters imposed by RetroSeq that it passes.

RetroSeq makes no attempt to determine the orientation or TSD of an insertion. Since RetroSeq does not pinpoint the exact location of a TE insertion, it does not provide predictions at base level accuracy. RetroSeq can optionally perform a highly computationally intensive step using Exonerate, that can sometimes cause the software to fail execution. To avoid this step, RetroSeq requires annotation files for each TE family in the reference genome. This means that RetroSeq cannot predict insertions of TE families with no copy in the reference genome, unless a modified version of the reference genome is supplied by the user.

No modifications were made to RetroSeq code.

PoPoolationTE

PoPoolationTE uses read-pair evidence to detect non-reference and reference TE insertions (Kofler *et al.*, 2012, 2015). The original application of PoPoolationTE was to analyse TE dynamics in populations of pooled sequencing data in *D. melanogaster*. PoPoolationTE version 1.02 is used by McClintock.

The inputs required to run PoPoolationTE are paired-end fastq sequencing data, a fasta reference genome, a fasta file of the canonical TE sequences, annotated locations of TEs in the reference genome in one-based GFF format, and a "hierarchy" file with the first column containing the name of each TE instance in the GFF annotation and the second column containing the TE family to which it belongs.

PoPoolationTE requires a special "extended reference" genome to be created. This involves using RepeatMasker to mask the reference genome of any TE sequences, and then modifying the reference genome to include new sequences of the canonical TE sequences plus sequences of any TE instances discovered by RepeatMasker as extra "chromosomes." Paired-end reads are mapped to this modified reference genome using the BWA-ALN algorithm. The resulting alignments are then processed by a script that marks paired-end reads where one read maps uniquely and the other maps to one of the TE "chromosomes". Locations where one paired-end read uniquely maps to the genome and the other read maps in the same direction and to the same TE family are then clustered if they are within a set distance from each other. Clusters of uniquely mapped reads at either end of a TE are then grouped together as a single insertion if they are for the same TE family and more than the read length but less than an empirically-determined threshold length (250 bp) apart. RepeatMasked sequence is not taken into account for the distance calculations, and thus this process allows reference as well as non-reference TEs to be detected using the same approach.

The output for PoPoolationTE is a custom text file which lists the predicted locations of non-reference and reference TE insertions on one-based coordinates. If a prediction is supported on only one side of the TE, then the annotation is the coordinates of the supporting cluster of reads plus a single location that is a fixed distance (100 bp by default) in the direction of where the insertion is predicted to occur. If an insertion is supported by reads on both sides, then the annotation is the coordinates of both supporting clusters of reads with a single location given as the midpoint between the innermost base pairs of each cluster. As reference TE sequences

are masked, the predictions of reference TEs are dealt with in the same way as non-reference TEs, and are also given single base pair coordinates based on the coordinates of the cluster(s) of reads that provide(s) the evidence for the insertion.

As PoPoolationTE does not use split-reads, it does not predict TE insertions to base pair accuracy. In addition, the process of dividing the range between read clusters by two to give an estimated location for an insertion can lead to genome coordinates with half base pairs to be reported. These half-base coordinates are non-standard in genomics and can cause problems with downstream analysis. Reference TEs are reported as a single base pair or a range containing the predicted reference TE, the same way non-reference TEs are reported. This means that reference insertions are not represented with base pair level accuracy as they are in the reference genome annotation. In addition, since detection of reference TEs is treated the same as non-reference TEs, reference TEs may be misinterpreted as non-reference TEs if PoPoolationTE does not identify the annotated TE sequence the insertion belongs to.

During testing of PoPoolationTE, it was discovered that a distance parameter used for clustering reads to be included in the same predicted insertion was hard-coded into the relevant script. Since this clustering parameter should match the properties of the library, McClintock patches the relevant script in PoPoolationTE during installation so that the empirical read length and insert size of the sample are taken into account for this clustering step. In addition, three other PoPoolationTE scripts are patched during the installation of McClintock to prevent a bug reporting fastq read IDs warnings producing large amounts of output, and also to prevent crashes from bugs relating to whether defined variables exist.

TE-locate

The final TE detection system in the McClintock pipeline is TE-locate, which uses read-pair evidence to detect non-reference and reference TE insertions (Platzer *et al.*, 2012). TE-locate was developed to detect TE insertions in *Arabidopsis thaliana* resequencing data. TE-locate version 1.0 is used by McClintock.

The inputs required to run TE-locate are a lexically sorted SAM file of paired-end NGS reads aligned a reference genome, a fasta file of the reference genome the alignment was performed against, and annotated locations of TEs in the reference genome in one-based GFF format.

Optionally, TE-locate can take as input a "hierarchy" file that indicates the relationship of individual TE sequences to a higher taxonomic level; for example, TE family or TE superfamily. This can then be used to assign non-reference insertions to a more generic taxonomic annotation, for example the TE family, rather than attempting to predict the exact reference copy that gave rise to an insertion.

To detect TE insertions, TE-locate identifies all paired-end reads that have one end mapped to an annotated TE location and the other end mapped uniquely and with good quality to unique genomic DNA. Clustering of reads mapping uniquely to the genome is used to refine predictions of TE insertions. This clustering allows paired-end reads mapped up to a user provided distance to be treated as support for the same insertion, with the recommended distance being three times the library insert size. To call an insertion, TE-locate requires at least three supporting read pairs.

The final output file of TE-locate is a custom text file listing the location of an insertion as a single base pair coordinate. For non-reference insertions, a one-based single base pair coordinate is reported along with the length of TE that potentially inserted at that site, and the orientation of the predicted insertion is given if it can be determined. For reference TEs, the annotation is a one-based single base coordinate taken from the start of the TE in the reference annotation and the length of that reference insertion, with no orientation provided (although this information can be obtained from the reference annotation provided as input). In addition, TE-locate reports the name of the TE detected and whether the TE is a non-reference or reference insertion.

TE-locate often produces predictions for non-reference TE insertions from different families in close proximity. It is not always possible to distinguish whether these are two true insertions of different TEs or simply one insertion that TE-locate incorrectly calls twice. TE-locate also reports predictions at a single base pair location, despite not actually producing predictions at base pair accuracy. TE-locate uses the annotation of TEs in the reference genome rather than a set of canonical TE sequences to detect non-reference insertions. This means it is not possible for TE-locate to predict insertions of TEs with no copy in the reference genome without providing a modified reference genome. Occasionally, TE-locate makes predictions that it reports as a reference insertion despite there being no reference TE annotation at that location.

No modifications were made to TE-locate code.

Overview of the McClintock process

From the limited set of inputs and options the user provides, McClintock then automatically generates all other input files required to run all six component methods. If the reference TE annotation and hierarchy file are provided by the user, the RepeatMasker step is skipped and the user-supplied reference TE annotation is used to make a hard-masked version of the reference genome using BEDTools (a step that is required only for PoPoolationTE). If the reference TE annotation and TE hierarchy file are not supplied by the user, McClintock launches RepeatMasker, which creates a reference TE annotation in GFF format that is in turn used by McClintock to create the TE hierarchy file. If specified, modifications can be made to the reference genome prior to automatic generation of the reference TE annotation and hierarchy file (see Options section in the main text). McClintock then converts the reference TE annotation to BED format, as required by TEMP and RetroSeq.

Prior to running any of the component methods, McClintock runs FastQC on the input fastq files to provide the user information to help interpret McClintock output. FastQC results are stored in a quality control subdirectory for each sample. Next, all indexing steps for the reference genome are performed. If only single-ended NGS data is provided, this is automatically detected by McClintock, and only the component methods that can analyse single-ended NGS data (ngs_te_mapper and RelocaTE) are launched. In this case, the main BWA-MEM alignment step is not performed because ngs_te_mapper and RelocaTE execute their own internal alignments. If paired-end NGS data is provided, then the main BWA-MEM alignment of the NGS data to the reference genome is launched and stored in SAM format. If TE-locate or TEMP are to be run, then the median insert size is calculated based on the distance between aligned pairs of reads in this SAM file. If TE-locate is to be run, then the SAM output of BWA-MEM is lexically sorted and a new SAM file is retained. If TEMP or RetroSeq are to be launched, then the SAM alignment file is sorted, converted into BAM format and indexed. In addition, if a BAM file is created, then McClintock will launch SAMtools flagstat to produce mapping summary statistics that are stored in the quality control subdirectory for each sample.

To launch ngs_te_mapper, the basic inputs to McClintock are sufficient and no additional preprocessing is required. To launch RelocaTE, "TSD=UNK" is automatically added to each identifier line in the canonical TE fasta file, providing maximum flexibility for this method. The custom reference TE annotation required by RelocaTE is produced from the user-supplied GFF or created from RepeatMasker output. To run TEMP, soft links are created to the BAM and

BAM index files to ensure they have the required suffixes ("sorted.bam" and "sorted.bam.bai," respectively). To run RetroSeq, the canonical TE file or reference TE annotation file is split into one file per TE family, and a file-of-files is produced with these file locations. For RetroSeq, McClintock uses the less computationally-intensive approach of assigning discordant reads to a TE family based on reference TE locations, rather than alignment of the reads to canonical TE sequences using Exonerate (we found the latter approach caused frequent failures during testing). The code to run the Exonerate step is included in McClintock if a user has data and a compatible computing environment. To launch PoPoolationTE, the basic TE hierarchy file is reformatted to add additional columns required by this method. Also, the identifiers of reads in the fastq input files are also changed so that they end with "\1" or "\2" for each member of a pair of reads. Finally, the median insert size of fragments is calculated based on the distance between aligned pairs of reads in a PoPoolationTE-specific SAM file (created using the BWA-ALN algorithm), and the read length is obtained from the fastq files. These values are passed to a patched version of PoPoolationTE that allows sample-specific parameters to be set for clustering TE-supporting reads. To run TE-locate, the reference TE annotation file is modified using the TE hierarchy file to ensure that the correct family level of annotation is provided in the column required by TE-locate. TE-locate also requires that the reference genome has more than five chromosomes. Should this not be the case, McClintock will add as many false chromosomes as required to produce five in total. Once these pre-processing steps are performed, each of the component methods are run following the guidelines described in their publications and manuals. (See Description of McClintock Component Methods above for further details).

To make McClintock runs more efficient for large resequencing datasets from the same species, input files that are reference genome specific but not sample specific (for example, genome indexes and reference TE annotations) are saved separately in the highest level of the output directory. If another sample is run for the same reference genome in the same output location, then these files can be reused, saving both space and time. As noted above, files that are not required, such as intermediate output and large genome alignments, can be deleted once used to minimize disk space held throughout the run. Also, if a subcomponent of McClintock is not run then, where possible, McClintock will not create any input files that are solely required for that method.

Post-processing and Standardization of Component Method Output

For ngs_te_mapper, predictions of non-reference TE insertions are produced as annotations of the TSD in zero-based coordinates in a BED format file. For reference insertions, the full span in the reference genome that is predicted to be TE sequence is annotated in zero-based coordinates in a BED format file. For both of these result types, data were reformatted slightly by McClintock to provide additional information to the ID and to add the orientation in the column dictated by the BED6 format specifications. All ngs_te_mapper predictions were annotated with "sr" in the name to denote that these predictions are based on split-read data. No other filtering steps were performed. There were no redundant predictions (of different TE families at the same coordinate) observed for non-reference TE insertions in ngs_te_mapper, so no redundancy removal was performed. In testing, some overlapping reference TE predictions were observed, though not with identical coordinates. These could not be removed automatically because reference TEs are often observed to overlap in reference genomes due to nested insertions.

RelocaTE produces multiple output files for each TE family in the canonical TE file provided as input. The main output file for each TE family is a GFF file. For non-reference predictions, the GFF data represents the TSD of the predicted insertion in one-based coordinates. For reference TE annotations, RelocaTE provides the span of the reference TE in one-based GFF format. If a reference TE is predicted to be present in the sample, RelocaTE denotes this TE as "shared" in the info field of the GFF file. For the data in each individual results file, the predictions were converted to zero-based coordinates, the file format was converted to BED6, and the results for different TE families were combined into a single file per sample. Only reference TEs with evidence for their presence in the sample were extracted and converted to BED6 format. As these reference TE predictions did not contain the orientation of insertion, this data was extracted from the reference TE annotation supplied to McClintock. All RelocaTE predictions were annotated with "sr" in the name to denote that predictions from this method are based on split-read data. Very rare cases of non-reference predictions for different TE families at the same coordinates were observed in testing with *S. cerevisiae*. In these cases, only the prediction that had the greatest number of supporting split-reads was retained. No other filtering step was performed for RelocaTE.

With TEMP, non-reference TEs are annotated either as a span containing the predicted TE in-

sertion when using only read-pair data, or as the predicted TSD when using split-read data. For non-reference TE predictions, TEMP outputs a custom file format where all coordinates are one-based. This custom file would occasionally produce impossible results where the coordinates had negative values; to prevent any errors in downstream analysis, these results were removed. Results are converted to BED6 by selecting the “start” and “end” columns for predictions that do not have split-read evidence for both junctions, and the “junction” columns where these are available for both termini of the predicted TE. One base coordinates are then converted to zero-based coordinates. Predictions of non-reference TEs that were based on read-pair data only were annotated with “rp”, and those that had split-read evidence were annotated with “sr”. Results from TEMP were first filtered to retain only non-reference TE predictions where there is evidence at both ends of an insertion and have a ratio of reads supporting the insertion to non-supporting reads of greater than ten percent. TEMP does not directly detect whether reference TEs are present in the sample, however the results of the absence module can be used to infer complementary information about the presence of reference annotated TEs. To obtain predicted reference TEs, BEDTools is used to subtract any TEs predicted to be absent by TEMP from the reference TE annotation used as input for McClintock. This leaves the set of reference TEs for which there was no evidence of absence in the resequencing data. These annotations are labelled with “nonab”, representing a TE inferred from non-absence to distinguish them from reference TE predictions based on direct evidence. TEMP’s absence module has a minor bug which causes the output to contain redundant annotations of the same predicted absence event. This redundancy in results from the TEMP absence module does not affect the output of McClintock because the complementary set of reference TEs is taken from TEMP absence results. Rare cases occur where two or more predicted non-reference TEs from different families share the same coordinates; these redundant predictions are resolved by retaining only the prediction that has the highest read support.

RetroSeq produces predictions for non-reference TEs in a one-based VCF file. The insertion is predicted to occur after the base that is annotated in the VCF position column. To convert RetroSeq predictions to BED format, the coordinates of the two bases that the non-reference TE insertion is predicted to occur between are used after converting to zero-based coordinates. This means the coordinate in the position column minus one is used as the start coordinate, and the position column plus one is used as the end coordinate. All predictions were annotated with “rp” in the name to denote these predictions are based on read-pair data. Predictions were filtered to retain only those that were assigned a call status of greater than or equal to six, as described in (Nellaker *et al.*, 2012). This call status represents predictions that have passed

filters for read depth of the call region, read threshold for a cluster supporting an insertion, total flanking reads, enough inconsistently mapped reads, and at least one side passing the ratio test. The status shows that a prediction only failed tests on distance at the breakpoint (greater than 120 bp between supporting clusters at either side of an insertion) and ratio of forward to reverse orientation support at only one end. Cases can occur where two predictions of different TE families share the same coordinates; in this situation redundant predictions were removed with the prediction with the highest genotype quality score retained.

PoPoolationTE provides annotations in one-based coordinates in a custom formatted output file. In addition to the spans defined by read clusters supporting each end of the predicted TE, a single base is provided estimating the location of the insertion. For predicted TEs with only one end supported, this base is given in the direction of the predicted insertion. Non-reference insertions were converted into BED format by taking the innermost coordinates of the supporting spans as the region in which an insertion is predicted to have occurred, and converting this interval to zero-based coordinates. Reference TE insertions are detected in the same way as non-reference insertions by PoPoolationTE and so the conversion process for reference insertions is similar. In the case of reference TEs, the innermost coordinates of the supporting spans are taken as the interval within which a reference TE sequence occurs and then converted to zero-based BED format. All PoPoolationTE predictions were annotated with "rp" in the name to denote they are based on read-pair data. Results are then filtered to retain only insertions that have evidence at both sides of the TE insertion and also have a ratio of reads supporting the insertion relative to reads supporting no insertion of greater than ten percent. Redundant predictions of different TE families at the exact same span are resolved by keeping the prediction that has the highest number of supporting reads.

TE-locate produces annotations in a one-based custom formatted output file. Non-reference TEs are annotated with a single base location and the length of the predicted insertion that may occur there. Reference TEs are annotated with a single base location as well as the length of the TE sequence. The coordinate for this base is converted to zero-based BED format in the McClintock output. To convert the reference TE prediction annotations to BED, the start coordinate is converted to zero-based coordinates, and the start location plus the length of the reference TE is used as the end coordinate. All TE-locate predictions are annotated with "rp" in the name to denote these predictions are based on read-pair data. No filtering is performed on the non-reference TE predictions. The reference TE results are filtered to remove any predictions that do not coincide with a reference TE annotation in the GFF input file. At this stage, orientation

annotation is also added to the reference TE predictions made by TE-locate using the input TE reference annotation GFF file. Any redundant predictions of different TE families at the same coordinates are removed with the prediction that has the highest read support retained.

If predictions for non-reference or reference TEs are made by any component method in the additional "chromosomes" added in modified reference genomes (see Options in Main Text), then these results are removed from the standard results files and retained in a subdirectory within the results directory called "non-ref_chromosome_results".

Simulating Resequencing of the *S. cerevisiae* Reference Genome

To test the performance of McClintock component methods, we simulated resequencing of the sacCer2 reference genome using WgSim (https://github.com/lh3/wgsim) (Li *et al.*, 2009) with parameters that resemble the properties of Illumina sequencing (as described by (Lee and Schatz, 2012)). Read lengths were chosen to be 101 bases each with an insert size of 300 bases to mimic the properties of the large sample of yeast genomes reported in Strope *et al.* (2015). One hundred simulated datasets were created at both 10X and 100X coverage of the 12Mb *S. cerevisiae* reference genome. To generate a coverage of 100X across the length of the sacCer2 reference genome (12,162,995 bp), 6,021,285 pairs of 101 bp per end were created for each simulated sample. To generate 10X coverage, seqtk (https://github.com/lh3/seqtk) was used to sub-sample ten percent of each of the 100X depth read sets.

To test the various different input options for each simulated sample, McClintock (version e945d20da22dc1186b97960b44b86bc21c96ac27) was run on each of the simulated datasets, with four different combinations of reference genome modifications: the unmodified reference genome, a modified reference genome including canonical TE sequences added as additional "chromosomes", a modified reference genome plus reference TE instances, and a modified reference genome with both canonical TE sequences and reference TE instances added (see Options section in Main Text). Canonical TE sequences were taken from Carr *et al.* (2012). Reference TE annotations were either taken from Carr *et al.* (2012) or produced automatically by McClintock using RepeatMasker. For the Carr *et al.* (2012) annotations, there are 483 reference TEs annotated, while RepeatMasker generates 564 reference TE annotations. RepeatMasker occasionally splits sequences that were annotated as one TE insertion in (Carr *et al.*,

2012) into multiple consecutive insertions of the same or closely related families. We note that the number of reads simulated for each sample was the same regardless of whether a modified reference genome option was used or not. The modified genome was only used by McClintock to test the effects of how these options influence TE predictions.

The mean and standard deviation of the number of reference TEs (Table S1) or non-reference TEs (Table S2) predicted per sample was calculated for all six component methods for all eight reference genome and reference TE annotation combinations.

Reference genome resequencing simulation: reference TE predictions

In our reference genome simulations, all reference TE sequences are present in the simulated samples and can theoretically be detected by all component methods. TE annotations from (Carr *et al.*, 2012) and RepeatMasker both include many clustered or nested TE insertions. It is known that mapping short reads uniquely in repetitive sequence is a problem (Treangen and Salzberg, 2012), and thus it is unlikely that any component method would be able to detect all reference TE insertions in reality. The average numbers of reference TEs predicted per sample across 100 simulations are shown in Table S1. Results are not shown for RetroSeq because this method does not have the ability to detect reference TE sequences.

ngs_te_mapper detected the lowest number of reference TEs at both coverage levels, much lower than the other split-read method, RelocaTE. A reason for this could be that ngs_te_mapper does not use a reference TE annotation to aid in calling a reference TE insertion. In addition to the low number of predictions, ngs_te_mapper is also non-optimal because this method can also produce multiple predictions of differing lengths for some reference TEs. In some cases these predictions represent true reference TEs that are in close proximity and that are falsely called as multiple overlapping TEs, rather than discrete insertions as they are in reality. Because of this effect it is not possible to effectively filter out which overlapping ngs_te_mapper reference predictions are truly redundant, as is done for non-reference TE predictions for other methods (see section Post-processing and Standardization of Component Method Output).

RelocaTE outperforms the other pure split-read method ngs_te_mapper for reference TE detection probably because RelocaTE takes the location of reference TEs as input. With this prior information, the threshold for making a reference TE prediction in the sample can be reduced.

RelocaTE only requires that one read supports one end of a reference TE to accept it as shared with the sample, rather than both ends as for ngs_te_mapper. Nevertheless, in all cases RelocaTE detects many fewer reference TEs than are present in the reference genome.

As noted above, unlike other component methods, TEMP does not predict reference TEs but rather produces predictions of reference TE absence. This difference in how reference TEs are called means that TEMP appears to perform almost perfectly in these simulation analyses. It is very unlikely that in the unmodified reference genome simulations there will be reads that map correctly across where a reference TE is annotated suggesting its absence. As such, a very low number of reference TE absence predictions are made by TEMP (a single TE in one sample at 100X coverage) and virtually all annotated reference TEs are added to the TEMP output by McClintock.

The result for PoPoolationTE shows numbers of reference TE predictions most similar to RelocaTE, but with lower performance at lower coverage, and vice versa, relative to RelocaTE. These difference could be explained by the requirement for PoPoolationTE to have at least 10 supporting reads to call an insertion. Like ngs_te_mapper and RelocaTE, many fewer reference TEs are predicted by PoPoolationTE than are present in the reference genome for all simulations. PoPoolationTE hard masks TE copies in the reference genome, then treats detection of reference and non-reference TEs in exactly the same way, which may explain the low number of reference TE predictions for this method relative to other read-pair methods like TE-locate.

TE-locate shows the best performance to detect reference TE insertions at both coverage levels, regardless of simulation conditions. Like RelocaTE, TE-locate uses information from the reference TE annotation to make predictions about the presence of reference TEs in a sample. This prior information, together with the ability to use read-pair data may explain the relatively high performance to detect reference TE insertions for this method. However, like all other methods that attempt to predict the presence of a reference TE, TE-locate detects many fewer reference TEs than are present in the reference genome for all simulation cases.

In general, Table S1 shows that all component methods except TEMP consistently detected more reference insertions with higher coverage resequencing data. The apparent insensitivity of TEMP to detect reference TEs as a function of coverage is likely an artifact because TEMP does not predict reference TEs per se, but rather produces predictions of reference TE absence,

which are converted to presence calls by McClintock to match the results from other component methods. Additionally, there does not appear to be a large effect on the numbers of reference TE predictions produced using different reference TE annotations or reference genome modifications. The main effect of different inputs is whether or not the annotation comes from Carr *et al.* (2012) or is automatically generated by RepeatMasker. This is to be expected since differences in reference TE annotation change the number and position of potential targets. RelocaTE is the only method that shows substantial differences in the numbers of reference TEs predicted depending on reference genome modification. When reference instances of TEs are added as additional "chromosomes", there is a considerable reduction in the number of reference TEs predicted by RelocaTE, suggesting that reads supporting some reference TE predictions map to additional chromosomes rather than their true location of the TE in the reference genome and are filtered out of the final results file.

Reference genome resequencing simulation: non-reference TE predictions

In addition to providing insight into the predicition of reference TE insertions, these unmodified genome simulations can be used to estimate the the false positive non-reference TE detection. Since simulated reads were created directly from the unmodified *S. cerevisiae* reference genome, non-reference TE insertions should not be detected by any of the McClintock component methods in these samples, and any non-reference TEs detected can be classified as false positives. Tables S2 shows that at both 10X and 100X coverage, the majority of component methods produce no false positive non-reference TE predictions in any of the 100 simulated datasets. The only exceptions are RelocaTE and PoPoolationTE, which both generate very low rates of false positive non-reference TE predictions. Changing the reference genome inputs or TE annotation provided to McClintock appears to have little influence on this conclusion.

For RelocaTE, false positive non-reference TE insertions were only observed in a very small number of samples. Closer inspection revealed that 16 simulated samples in the 100X datasets produced a single false positive prediction of either *Ty1* or *Ty2* in approximately the same location (sacCer2, chrXIV 102523-102548) for each reference genome and reference TE annotation combination. Two samples also had the same false positive prediction at 10X coverage. At this location, a *Ty1* element is separated from a *Ty3* element in the reference genome by only 110 bp and so it is possible some mismapping is occurring due to the repetitive nature of the reference sequence in this region.

PoPoolationTE produced 1-2 false positive non-reference TE insertion in all samples, regardless of coverage, or reference TE annotation. Inspection of these false positive non-reference TE predictions revealed them to be caused by PoPoolationTE failing to assign a reference TE ID to one of its predictions, causing a reference TE to be mislabelled as a non-reference TE prediction. Since PoPoolationTE always uses its own modified reference genome for input (see Options in Main Text), the only variation between the eight input combinations expected for this method is between the use of either RepeatMasker or the annotation of Carr *et al.* (2012) reference TE annotations. The four tests run using the Carr *et al.* (2012) annotation as input and the four using the RepeatMasker produced annotation produce slightly different numbers of false positive non-reference TE despite being given identical input. This suggests that the results of PoPoolationTE non-reference TE detection are probabilistic to a small degree.

Overall these results show that the majority of McClintock component methods do not produce many false positive predictions, at least within the context of our simulation. In addition, manipulating coverage, changing the reference TE annotation, or adding TE sequences to reference genome as new "chromosomes" does not substantially affect the rate of false positive non-reference TE predictions. These results form the basis of interpreting results for simulated reference genomes with single synthetic insertions described in the Main Text.

Supplemental References

Carr, M., D. Bensasson, and C. M. Bergman, 2012 Evolutionary genomics of transposable elements in Saccharomyces cerevisiae. PLoS ONE **7**: e50978.

Keane, T. M., K. Wong, and D. J. Adams, 2013 RetroSeq: transposable element discovery from next-generation sequencing data. Bioinformatics **29**: 389–390.

Kofler, R., A. J. Betancourt, and C. Schlötterer, 2012 Sequencing of pooled DNA samples (pool-seq) uncovers complex dynamics of transposable element insertions in Drosophila melanogaster. PLoS Genet **8**: e1002487.

Kofler, R., V. Nolte, and C. Schlötterer, 2015 Tempo and Mode of Transposable Element Activity in Drosophila. PLoS Genet **11**: e1005406.

Lee, H. and M. C. Schatz, 2012 Genomic dark matter: The reliability of short read mapping illustrated by the genome mappability score. Bioinformatics **28**: 2097–2105.

Li, H., B. Handsaker, A. Wysoker, T. Fennell, J. Ruan, N. Homer, G. Marth, G. Abecasis, R. Durbin, and . G. P. D. P. Subgroup, 2009 The Sequence Alignment/Map format and SAMtools. Bioinformatics **25**: 2078–2079.

Linheiro, R. S. and C. M. Bergman, 2012 Whole genome resequencing reveals natural target site preferences of transposable elements in Drosophila melanogaster. PLoS One **7**: e30008.

Nellaker, C., T. M. Keane, B. Yalcin, K. Wong, A. Agam, T. G. Belgard, J. Flint, D. J. Adams, W. N. Frankel,

and C. P. Ponting, 2012 The genomic landscape shaped by selection on transposable elements across 18 mouse strains. Genome Biol **13**: R45.

Platzer, A., V. Nizhynska, and Q. Long, 2012 TE-Locate: a tool to locate and group transposable element occurrences using paired-end next-generation sequencing data. Biology **1**: 395–410.

Robb, S. M. C., L. Lu, E. Valencia, J. M. Burnette, Y. Okumoto, S. R. Wessler, and J. E. Stajich, 2013 The use of RelocaTE and unassembled short reads to produce high-resolution snapshots of transposable element generated diversity in rice. G3 **3**: 949–957.

Strope, P. K., D. A. Skelly, S. G. Kozmin, G. Mahadevan, E. A. Stone, P. M. Magwene, F. S. Dietrich, and J. H. McCusker, 2015 The 100-genomes strains, an S. cerevisiae resource that illuminates its natural phenotypic and genotypic variation and emergence as an opportunistic pathogen. Genome Res. **25**: 762–774.

Treangen, T. J. and S. L. Salzberg, 2012 Repetitive DNA and next-generation sequencing: computational challenges and solutions. Nature Reviews Genetics **13**: 36–46.

Zhuang, J., J. Wang, W. Theurkauf, and Z. Weng, 2014 TEMP: a computational method for analyzing transposable element polymorphism in populations. Nucleic Acids Res. **42**: 6826–6838.

File S2. BED files with McClintock predictions for 93 yeast genome in SRA072302.

File S3. Code used to generate simulated yeast genomes and apply McClintock to simulated and real yeast genome data.
