# Supplementary material for: McClintock: An Integrated Pipeline for Detecting Transposable Element Insertions in Whole-Genome Shotgun Sequencing Data
Source: G3 (Bethesda). 2017 Jun 21;7(8):2763–78. doi: 10.1534/g3.117.043893 (PMC5555480; doi:10.1534/g3.117.043893)
Supplement: Supplementary file 3 [file 2763FileS3.zip › mcclintock_analysis_code/seqplots-master/inst/doc/QuickStart.html]

SeqPlots R workflow


# SeqPlots R workflow

#### *Przemyslaw Setmpor*

#### *16 September 2015*

# Quick start demo

1. Start SeqPlots. Refer to **installation guides** for platform specific information. After successful initiation the web interface should automatically open in your default web browser. If you are using web server version just navigate your browser to the server address.
2. Upload feature (BED or GFF) and track (BigWig or WIG) files. They can be gzip compressed (e.g. file1.bed.gz). Press green “Add files…” button or just drag and drop files into the window. The ready to upload files will show up in upload window, where you select user name, reference genome and optionally add some comments.
3. When all is done press blue “Start upload” button. After upload and processing is done the green “SUCCESS” label should show. It means that file is on the registered and ready to use. Occasionally the file might be mot formatted properly or chromosome names might not agree with reference genome. In such case a verbose error will window appears and file as labeled as “ERROR”. For further information please refer to **errors chapter**.
4. Dismiss upload window and press blue “New plot set” button on side panel. This will bring up file management window. In file management window select at least one file from “Features” tab and at least one file from “Tracks” or sequence motif(s). The sequence motifs and tracks can be processed and plotted together. Select files by clicking on file name, selected files will be highlighted.
5. After choosing files/motifs to plot, set up the processing options. You can find these in the button of plotting window.
6. After options are set up press blue “Run calculation” button. This will dismiss the file management window and show processing message. Here you can observe the progress of the task and optionally cancel it if no longer required or you forgot to add some important file to the plot-set.
7. After some time the calculation will finish (fingers crossed, without the error) and you will be able to see plot set array. In here you can choose which feature-track or feature-motif pairs to plot. Choose one or more checkboxes and press grey “Line plot” button (or hit RETURN from your keyboard).
8. Congratulation! Your First plot is complete, you can see the preview of it on the side panel.
9. You are able to set up labels, titles, font sizes, legends and many more on control panel tabs - please see **Plotting** chapter for details.
10. By clicking the plot preview you can enlarge it for better view. When everything is ready you can get the plot as PDF by clicking green “Line plot” button just on the top of side panel.
11. You can also visualize the signal as a heatmap. Please note that heatmap plotting is possible only for a single feature file. It is possible to sort heatmaps based on mean row signals and/or cluster them using k-means algorithm, hierarchical clustering or self organising maps. To learn more about heatmaps see **Heatmaps** chapter.
12. Heatmaps can be downloaded sa PDFs using ‘Heatmap’ button just on the bottom of setup panel. The small button, at the right sied of ‘Heatmap’ button downloads cluster definition and sorting order.

# Session Information

```
## R version 3.2.2 (2015-08-14)
## Platform: x86_64-apple-darwin13.4.0 (64-bit)
## Running under: OS X 10.10.2 (Yosemite)
## 
## locale:
## [1] en_US.UTF-8/en_US.UTF-8/en_US.UTF-8/C/en_US.UTF-8/en_US.UTF-8
## 
## attached base packages:
## [1] stats4    stats     graphics  grDevices utils     datasets  methods  
## [8] base     
## 
## other attached packages:
##  [1] seqplots_1.7.2                    ggplot2_1.0.1                    
##  [3] BSgenome.Celegans.UCSC.ce10_1.4.0 BSgenome_1.36.3                  
##  [5] rtracklayer_1.28.10               Biostrings_2.36.4                
##  [7] XVector_0.8.0                     GenomicRanges_1.20.6             
##  [9] GenomeInfoDb_1.4.2                IRanges_2.2.7                    
## [11] S4Vectors_0.6.5                   testthat_0.10.0                  
## [13] BiocGenerics_0.14.0               devtools_1.8.0                   
## 
## loaded via a namespace (and not attached):
##  [1] Rcpp_0.12.0             Rsamtools_1.20.4       
##  [3] class_7.3-14            digest_0.6.8           
##  [5] mime_0.4                R6_2.1.1               
##  [7] plyr_1.8.3              futile.options_1.0.0   
##  [9] evaluate_0.7.2          RSQLite_1.0.0          
## [11] spam_1.0-1              httr_1.0.0             
## [13] zlibbioc_1.14.0         lazyeval_0.1.10        
## [15] curl_0.9.3              rstudioapi_0.3.1       
## [17] DT_0.1.31               rmarkdown_0.8          
## [19] proto_0.3-10            BiocParallel_1.2.21    
## [21] stringr_1.0.0           htmlwidgets_0.5        
## [23] RCurl_1.95-4.7          munsell_0.4.2          
## [25] shiny_0.12.2            httpuv_1.3.3           
## [27] rversions_1.0.2         htmltools_0.2.6        
## [29] gridExtra_2.0.0         roxygen2_4.1.1         
## [31] codetools_0.2-14        XML_3.98-1.3           
## [33] crayon_1.3.1            GenomicAlignments_1.4.1
## [35] MASS_7.3-44             bitops_1.0-6           
## [37] grid_3.2.2              jsonlite_0.9.17        
## [39] xtable_1.7-4            gtable_0.1.2           
## [41] DBI_0.3.1               formatR_1.2            
## [43] git2r_0.11.0            magrittr_1.5           
## [45] covr_1.2.0              scales_0.3.0           
## [47] stringi_0.5-5           kohonen_2.0.19         
## [49] reshape2_1.4.1          rex_1.0.1              
## [51] xml2_0.1.2              futile.logger_1.4.1    
## [53] BiocStyle_1.6.0         lambda.r_1.1.7         
## [55] RColorBrewer_1.1-2      tools_3.2.2            
## [57] Cairo_1.5-8             maps_2.3-11            
## [59] fields_8.2-1            yaml_2.1.13            
## [61] plotrix_3.5-12          parallel_3.2.2         
## [63] colorspace_1.2-6        memoise_0.2.1          
## [65] knitr_1.11
```
