# Supplementary material for: McClintock: An Integrated Pipeline for Detecting Transposable Element Insertions in Whole-Genome Shotgun Sequencing Data
Source: G3 (Bethesda). 2017 Jun 21;7(8):2763–78. doi: 10.1534/g3.117.043893 (PMC5555480; doi:10.1534/g3.117.043893)
Supplement: Supplementary file 3 [file 2763FileS3.zip › mcclintock_analysis_code/seqplots-master/inst/doc/SeqPlotsGUI.html]

SeqPlots R workflow


# SeqPlots R workflow

#### *Przemyslaw Setmpor*

#### *16 September 2015*

- Adding and managing files
  - Supported file formats
  - Adding files
  - Downloading and removing files
- Running the plot-set jobs
  - Selecting files
  - Setting up plot options
  - Plotting sequence motif density
  - Starting the plot set calculation
- Plotting
  - Previewing plot
  - Titles and axis panel
  - Guide lines and data scaling
  - Keys, labels and colors panel
  - Heatmap setup tab
  - Other options controlling heatmap appearance
- Getting PDFs and cluster info
  - PDF output size
- Batch operations
  - Load or save plotset
  - Plot set files structure
- Advanced options
- Genomes managment
- Error messages
- Explanations
- References
- Session Information

# Adding and managing files

## Supported file formats

Tracks:

- BigWig (.bw) - http://genome.ucsc.edu/FAQ/FAQformat.html#format6.1
- Wiggle (.wig) - http://genome.ucsc.edu/goldenPath/help/wiggle.html
- BedGraph (.bdg) - http://genome.ucsc.edu/goldenPath/help/bedgraph.html

Features:

- BED - http://genome.ucsc.edu/FAQ/FAQformat.html#format1
- GFF - http://genome.ucsc.edu/FAQ/FAQformat.html#format3
- GTF (with .gff extension) - http://genome.ucsc.edu/FAQ/FAQformat.html#format4

Files must be formatted according to UCSC guidelines. All widely used chromosome names conventions are accepted, e.g. for human files either ‘chr1’ or ‘1’ can be used, however these conventions should not be mixed within single files.

## Adding files

Press the `Add files` button to bring up the **file upload panel**.


You can drag and drop files here or press the `Add files...` button to open a file selection menu. Before starting the upload the following mandatory information must be provided about each file:

- User ID
- Reference genome - drop-down menu containing reference genome package currently installed in R

Comments are optional.

The contents of the text field can be copied to all files by clicking the icon at the left of the field. The default values can be set using `Set defaults...` button. Default values are stored using the browser cookies, and the settings will be remembered across different sessions as long as the same web browser is used. File extensions that are not supported will raise an error.


Individual files can be uploaded by pressing ‘start’ next to the file name or all files can be uploaded at once by pressing the `Start upload` button at the top of **file upload panel**.

During the upload process a progress bar is displayed. After upload SeqPlots gives a message that upload was successful or or gives an error message. Common errors are misformatted file formats or chromosome names do not matched the reference genome. For more information please refer to *Error explained* chapter.


To dismiss the upload window, click on `X` or outside the window.

## Downloading and removing files

Clicking the `New plot set` button brings up the **file collection window**. The primary function of this window is to choose signal tracks and feature files to use for calculating the plots. However, it also provides basic file management capabilities. Information on files can be reviewed and files can be downloaded or deleted. Fields can be searched, filtered and sorted by any column. The red `x` button on the right site of file table removes a single file from the collection, while `Remove selected files` button will erase all selected files.

# Running the plot-set jobs

Pressing the `New plot set` button brings up the **file collection window** from which you can choose signal tracks and feature files to calculate average plots and heat maps. If you wish to upload more files please refer to adding new files documentation. The **file collection window** has three tabs:

- `Tracks` - signal files, i.e., Wiggle, BigWiggle and BedGraph files.
- `Features` - genomic feature files, i.e., BED, GFF and GTF files
- `Sequence features` - input any motif of interest that you want to plot.


## Selecting files

The `Tracks` and `Features` tabs displays information about the files and allows you to filter and sort by any column. The “Search:” dialog allows you to find any keyword in any field, while dropdowns below the file grid allow for more advanced filtering on specific columns.

Select files by clicking on the file name or any other part of the row beside `Show comment` and `Download` or `Remove` buttons. Chosen files are highlighted in light blue. Clicking the file name again will cancel the selection. At least one signal track or motif and one feature file must be selected before starting the calculation.

## Setting up plot options

Options controlling the plot settings is found below the file selection window:

1. **`Bin track @ [bp]:`** - this numeric input determines the resolution of data acquisition; the default value 10 means that 10bp intervals within the plotting range will be summarized by calculating the mean. Higher values increases the speed of calculation, but decreases resolution. See the explanations.
2. **`Choose the plot type`** - there are three options:
   - *`Point Features`* - anchor plot on the start of a feature. By default, plot will be directional if strand information is present (i.e, use start position and plot on positive strand for + strand features and use end position and plot on negative strand for minus strand features). If strand information is not present in the feature file (or if the “ignore strand” option is chosen), plot will use start position of feature and be plotted on the positive strand (see explanations). User chooses length of upstream and downstream sequence to plot.
   - *`Midpoint Features`* - similar to point feature, but plot is centered on the midpoint of the feature.
   - *`Endpoint Features`* - similar to point feature, but plot is centered on the end of the feature. Strand information is used by default to determine the end side.
   - *`Anchored Features`* - features are anchored at start and stop positions and given pseudo-length chosen by the user. Additionally, the user chooses the length of sequence upstream of the start and downstream of the end to plot.
3. **`Ignore strand`** - the directionality (strand) will be ignored all features plotted on the positive strand.
4. **`Ignore zeros`** - signal values of 0 in the track will be be excluded from calculations
5. **`Calculate heatmap`** - selecting this generates and saves a heat map matrix. Select if you wish to generate heatmap; uncheck if you only wish to generate average plots, as this will speed calculations.
6. **`Plotting distances in [bp]`** - the distances in to be plotted:
   - *`Upstream`* - the plotting distance in base pairs upstream to the feature
   - *`Anchored`* - the pseudo-length, to which the features will be extended or shrunk using linear approximation (only for anchored plots)
   - *`Downstream`* - the plotting distance in base pairs downstream to the feature

## Plotting sequence motif density

The `Sequence features` tab allows you to calculate and plot the density of any user-defined motif around the chosen genomic feature using the reference sequence package. Motif plots can be mixed with track files’ signal plots. The following options can be set:

1. **`DNA motif`** - the DNA motif
2. **`Sliding window size in base pairs [bp]`** - the size of the sliding window for motif calculation. The value (number of matching motifs within the window) is reported in the middle of the window, e.g. if window is set to 200bp, DNA motif is “GC” and there are 8 CpGs in first 200 bp of the chromosome the value 8 will be reported at 100th bp.
3. **`Display name`** - The name of the motif that will be shown in key and heatmap labels. Leave blank to use `DNA motif` value.
4. **`Plot heatmap or error estimates`** - this checkbox determines if heatmap matrix and error estimates should be calculated. If unchecked much faster algorithm will be used for motif density calculation, but only the average plot without the error estimates will be available.
5. **`Match reverse complement as well`** - select if reverse complement motif should be reported as well. For example the TATA motif will report both TATA and ATAT with this option selected.


Clicking `Add` button adds the motif to plot set, while `Reset All` clears the motif selection. On the right side of the motif setting panel gives a list summary of included motifs.

## Starting the plot set calculation

The options are executed by pressing the `Run calculation` button. This dismisses the **file collection window** and brings up the calculation dialog, which shows the progress. On Linux and Mac OS X (systems supporting fork based parallelization) the calculation can be stopped using the `Cancel` button - this will bring back all settings in **file collection window**.


After successful execution the **plot array** and **plot preview panel** will appear. In case of error an informative error pop-up will explain the problem. Please refer to the error section for further information.

# Plotting

> This section focuses on average (line) plots and options common between these and heatmaps. For heatmap options please refer to heatmap documentation.

## Previewing plot

After calculating or loading a plot set, a **plot array** of checkboxes is displayed to select the desired pairs of features and tracks/motifs. Clicking on the column name (tracks/motifs) or row name (features) selects/deselects the whole column or row. Clicking on top-left most cell of **plot array** toggles the selection of whole array.


If at least one pair on **plot array** is selected pressing the `Line plot` button produces an average plot preview and the `Heatmap` button produces a heatmap preview. Alternatively, pressing the [RETURN] key will also produce the new selection and options. These operations are done automatically in reactive mode (see **Advanced options** chapter). Plots can be downloaded as PDF files using the Line plot and Heatmap buttons next to Download (at the top of the panel).

Below the plotting buttons are options for labeling plots and setting axes. On application start the first panel responsible for bringing file upload, management and plot set calculation modals is active. The further three panels hold common plot settings.

## Titles and axis panel


This panel groups settings influencing the plot main title, axis labels, various font sizes plus vertical and horizontal plot limits.

- `Title` - The main title of the plot, shown in top-center part of the figure; default empty
- `X-axis label` - Label shown below horizontal axis; default empty
- `Y-axis label` - Label shown below vertical axis; default empty
- `Title font size` - Font size of the title in points (point = ~1/72 an inch for standard A4 output); default 20 points
- `Labels font size` - Font size of axis labels in points; default 16 points
- `Axis font size` - Controls axis ticks font size, that is size of the numbers indicating position in base pairs on X-axis and means signal value on X-axis; default 14 points
- `Set X-axis limits` - Set hard plotting limits for X-axis; default values are whole range chosen during plot set calculation
- `Set Y-axis limits` - Set hard plotting limits for Y-axis; default values are a range between lowest and highest mean signal extended by error estimate

## Guide lines and data scaling


Controls in this panel controls the display of guide lines and error estimates, and allows to log scale the signal prior to plotting.

- `Transform signal` - if set to *`Log2 transform`* performs log2 transformation of the signal prior to plotting; default setting is *`Do not transform`*
- `Show vertical guide line` - show the vertical line at point 0 - beginning of the feature or midpoint and end of the pseudo-length scaled features (only for anchored plots); turn on by default
- `Show horizontal guide line` - show the horizontal line at user determined height; turn off by default
- `Show error estimates` - show error standard error and 95% confidence interval as fields, if turned off only the line representing the mean signal is shown; turn on by default

## Keys, labels and colors panel


This panel groups two types of controls. `Colors`, `Label` and `Priority/Order` are a checkboxes revealing further controls on **plot set grid**, specific for a feature-track pair or sub-heatmap. `Show plot key`, `Show error estimate key` and `Legend font size` re global controls specific for average plots. Inputs on **plot set grid** do not have specific labels, but the tooltip explaining their meaning is shown on mouse cursor hover.

- `Colors` - checkboxes revealing a color picker on **plot set grid**. This input allows to control the colors of specific feature-track pair average plots or sub-heatmaps. In browser supporting the color picker ‘e.g Chrome’ the system dialog will show up. In other browsers (e.g. Firefox) the javaScript color picker will be initialized.
- `Label` - checkboxes revealing a label text input **plot set grid**. This controls the names shown on the **key** with average plots or the heatmap top labels.
- `Priority/Order` - checkboxes revealing numeric input on **plot set grid**. These number determine the order of average plots and hetamaps. Feature-track pair with the highest priority will be listed on the top of **key** for average plots and left-most for heatmaps.
- `Show plot key` - shows the key giving the color to feature-track pair label mapping. If turned on the additional drop-down allows to choose the position on the plot, top-right by default
- `Show error estimate key` - shows the key gexplaining the meaning of error fields. If turnedon the additional drop-down allows to choose the position on the plot, top-left by default
- `Legend font size` - set the size of font used to plot the keys; 12 default Heatmaps ========

Heatmaps are often more informative than average plots. If there is variability in signal along individual instances of a given genomic feature (e.g., because there are different biological classes), an average plot might not represent the behavior of any individual feature and could even give a misleading picture. SeqPlots plots track-feature pairs as sub-heatmaps horizontally aligned on single figure. All sub-heatmaps must have the same number of data rows, hence in single plot mode simultaneous plotting is possible only on single features or feature files containing exact same number of rows. The heatmaps can be sorted and clustered by k-means, hierarchical clustering or super self organising maps (SupreSOM).

## Heatmap setup tab

This tab has heatmap specific options for data processing and display.


- **`Sort heatmap rows by mean signal`** - sorts the heatmap rows based on the mean value of each row across included sub-heatmps. Can be set to increasing or decreasing order. Turned off by default.
- **`Clustering algorithm`** - choose clustering algorithm (k-means, hierarchical or SupreSOM). If clustering is not desired, choose *`do not cluster`*, which uses the feature file in the uploaded order . K-means by default.
- **`Make cluster calculation repeatable`** - enforces, that clustering with non-deterministic algorithms, like k-means or SupreSOM will generate the same results as most recently plotted heatmap. This is achieved by re-using R random number generator seed.
- **`Plot selected cluster`** - this option is available only if `Make cluster calculation repeatable` is turned on. Allows to select one of the clusters and zoom it to whole plot height. Plot all clusters by default.
- **`Choose individual heatmaps for sorting/clustering`** - this checkbox brings up a new control panel on the **plot set grid** to determine if a given sub-heatmap should be included in plotting and/or clustering. The excluded sub-plots will be plotted in the order of the other sub-heatmaps, but their values will not influence the clustering/sorting. By default all sub-heatmaps are included.
- **`Heatmaps have individual color keys`** - by default all sub-heatmap have their own color keys. This option determines if each sub-heatmap should have a separate color key (plotted below the heatmap) or a single, common key should be calculated for all sub-plots (plotted rightmost). The example below show the difference between separate (left) and common (right) color keys:
- **`Set default color key limits`** - this option determines the limits in mapping the numerical values to the colors. The range of colors generated is dependent on these options. Values lower or higher than the given limits will be plotted in the limit value color. If this checkbox is not selected, limits are auto-generated using **`Color key scaling`** parameter. If this option it turned off two numerical fields, **`min`** and **`max`**, are shown to manually set the limits.

  - **`Color key scaling`** - this slider influences how color key limits are generated. For example, 0.01 (default value) calculates limits using data ranging from 1-99 percentile of available data points. 0.1 uses data ranging from 10-90 percentile. The general formula for limit is: [quantile(data, `Color key scaling`); quantile(data, 1-`Color key scaling`)]
  - **`min`** and **`max`** numeric inputs - enter values to manually specify color key limits as numeric values.
- **`Set individual color key limits`** - this option is similar to manual set up of color key limits, but this allows one to specify different values for individual sub-heatmaps. When this checkbox is selected **`min`** and **`max`** numeric input menu is shown on the **plot set grid**
- **`Set colorspace`** - This input allows to use color palettes from RColorBrewer package, which would replace default color palette for heatmaps. By selecting **`Reverse`** checkbox the reversed color palette will be used. Here are available color pallets (click here to see example heatmap plotted with different color palettes):

  When the `Custom` option is selected three-color pickers are shown to setup custom color mappings for heatmaps. The following example below shows standard jet colors (left), default blue color mapping after selecting the checkbox (middle) and custom color selection (right):

## Other options controlling heatmap appearance

The heatmap output shares many display options from other tabs. Here we provide a list of these inputs, please refer to “Viewing and manipulating plots” for further reference.

- **Titles and axis panel**
  - `X-axis label` - Label shown below horizontal axis, drawn separately for each sub-heatmap; default empty
  - `Y-axis label` - Label shown next to vertical axis, drawn separately for each sub-heatmap; default empty
  - `Labels font size` - Font size for axis labels and main labels of sub-heatmaps; default 16 points
  - `Axis font size` - Controls axis ticks font size; default 14 points
  - `Set X-axis limits` - Set hard plotting limits for X-axis; default values are whole range chosen during plot set calculation
- **Guide lines and data scaling panel**
  - `Transform signal` - if set to *`Log2 transform`* performs log2 transformation of the signal prior to plotting; default setting is *`Do not transform`*
  - `Show vertical guide line` - show the vertical line at point 0 - beginning of the feature or midpoint and end of the pseudo-length scaled features (only for anchored plots); turn on by default
- **Keys, labels and colors panel**
  - `Colors` - for hetmaps this input allows to control the color mapping of specific sub-heatmaps. The map starts with white (for low color key limit) and finishes with selected color (for high color key limit).
  - `Label` - allows to set up custom sub-heatmap top labels
  - `Priority/Order` - Use this to place heatmaps in your desired order. The feature-track pairs with the highest priority will be plotted as left-most sub-heatmaps.
  - `Legend font size` - control the font size of common color key, inactive if heatmaps have individual color keys; 12 default

# Getting PDFs and cluster info

Plots can be downladed as PDFs by clicking `Line plot` or `Heatmap` buttons in the “Download:” section of the **tool panel** (above the plot preview).


The small buttons next to `Line plot` and `Heatmap` produce additional output files:

- the `i` button next to `Line plot` downloads the PDF containing average plot keys
- `cluster diagram` button next to `Heatmap` downloads a cluster report giving cluster assignments and sorting order for each feature as a comma separated value (CSV) spreadsheet.

The cluster report contains following columns:

- `chromosome` - the name of chromosome, contig or scaffold
- `start` - start of the feature (1 based chromosomal coordinate)
- `end` - end of the feature (1 based chromosomal coordinate)
- `width` - width of the feature in base pairs
- `strand` - strand of the feature
- `metadata_...` - annotation columns present in the original GFF/BED e.g. gene name, score, group
- `originalOrder` - number of feature (row) in GFF/BED, can be used to restore original order after sorting on cluster ID
- `ClusterID` - the numeric ID of the cluster. The topmost cluster on the heatmap is annotated with 1, and the bottom cluster with k, where k equals to number of clusters selected, exported only if clustering is enabled
- `SortingOrder` - the order imposed on heatmap by sorting by mean row(s) values, exported only if sorting is enabled
- `FinalOrder` - the final order of heatmap’s rows, this can be influenced by sorting and clustering; 1 indicates topmost row

Sample report:

```
chromosome  start   end     width   strand  metadata_group  originalOrder   ClusterID   SortingOrder    FinalOrder
chrI        9065087 9070286 5200    +       g1              1               1           3               3
chrI        5171285 5175522 4238    -       g1              2               3           50              43
chrI        9616508 9618109 1602    -       g1              3               3           13              43
chrI        3608395 3611844 3450    +       g1              4               3           11              12
```

Table view:

| chromosome | start | end | width | strand | metadata\_group | originalOrder | ClusterID | SortingOrder | FinalOrder |
| --- | --- | --- | --- | --- | --- | --- | --- | --- | --- |
| chrI | 9065087 | 9070286 | 5200 | + | g1 | 1 | 1 | 3 | 3 |
| chrI | 5171285 | 5175522 | 4238 | - | g1 | 2 | 3 | 50 | 43 |
| chrI | 9616508 | 9618109 | 1602 | - | g1 | 3 | 3 | 13 | 43 |
| chrI | 3608395 | 3611844 | 3450 | + | g1 | 4 | 3 | 11 | 12 |

## PDF output size

The last tab (`Batch operation and setup`) on the **tool panel** includes batch operations and various other settings including PDF output size. By default the output PDF will be A4 landscape. This can be changed using the drop-down list to following settings:

- `user defined` - this option reveals two numeric inputs that allows to set output PDF width and height. The values must be given in inches.
- `Legal rotated` - US Legal landscape: 14" by 8.5"
- `A4` - A4 portrait: - 8.27" × 11.69"
- `Letter` - US Letter portrait: 8.5" × 11"
- `Legal` - US Legal portrait: 8.5" × 14"
- `Executive` - a.k.a Monarch paper: 7.25 × 10.5"

# Batch operations

Controls to plot multiple plots at once are located on the `Batch operation and setup` tab, just below PDF paper options. It is possible to output the plots to multipage PDF, plot an array of plots on a single page (for average plots) or mix these options together.


The first drop-down controls the type of the plot - either average or heatmap. The second drop down determines the strategy to traverse the **plot grid**. The options include:

- `single` - every single feature-track pair will be plotted on separate plot
- `rows` - the **plot grid** will be traversed by rows, which means one plot that contains all tracks per feature will be prepared
- `columns` - the **plot grid** will be traversed by columns, which means one plot that contains all features per tracks will be prepared

The `multi plot grid` option controls how many plots will be placed on each page of the PDF output, e.g. 1x1 means one plot per one page, while 3x4 means 3 columns and 4 rows of plots. If number of plots exceeds the number of slots on page the new page will be added to the PDF.

`Filter names` will apply a filter to plot titles, which are based on on uploaded file names. For example, if you uploaded 100 files starting with a prefix of “my\_experiment\_”, you can remove this fragment from each plot title and/or heatmap caption by putting this string in `Filter names`.

Finally, pressing `Get PDF` produces the final output file. Please see example below:

> Saving and loading plotsets ============================

If desired, SeqPlots will save plot sets as binary R files, allowing you to quickly load the pre-calculated set for replotting. Saved plot sets can also be shared with other SeqPlots users.

## Load or save plotset

Controls available on the “Load or save plotset” panel:

- **`Load saved plot set`** - drop-down list to select a plotset. Once the Rdata binary file is selected the **plot grid** will be displayed. Selecting the file reveals two additional buttons:
  - **`Remove dataset`** - this button deletes the selected saved plot set from user data.
  - **`Download plotset`** - this button saves a copy of the plotset in selected location.
- **`Save current plot set`** - Enter desired name and press the `Save` button (appears after input of name). It is also possible to save a copy of loaded plot sets. The plot set binary files can be renamed simply by loading them, saving a copy and deleting original source file.

All saved dataset can be found in `data location`/publicFiles. Any SeqPlots Rdata binaries put in the folder will become available for loading in **`Load saved plot set`** control.

## Plot set files structure

The plot sets files can be also directly loaded in R. This allows further processing and customization of the plots. Data structure is a nested list, which elements be accessed by `[[` R operator. The nesting goes as follow:

- **`feature`** - R list
  - **`track`** - R list
    - `means` - numeric vector giving mean signal value for each (binned) genomic position
    - `stderror` - numeric vector giving standard error for each (binned) genomic position
    - `conint` - numeric vector giving 95% confidence interval for each (binned) genomic position
    - `all_ind` - numeric vector giving the genomic position in base pairs
    - `e` - character string giveing numeric vector giving the indicates of anchored distance, NULL for point features plots
    - `desc` - auto generated title of the plot
    - `heatmap` - numeric matrix, (binned) signal values for each genomic position (columns) and each feature (rows)

The example structure:

```
List of 2
 $ HTZ1_Differential_genes_TOP100_v2.gff:List of 2
  ..$ HTZ1_JA00001_IL1andIL2_F_N2_L3_NORM_linear_1bp_IL010andIL009_averaged.bw    :List of 7
  .. ..$ means   : num [1:501] 2.52 2.52 2.52 2.53 2.54 ...
  .. ..$ stderror: num [1:501] 0.114 0.112 0.111 0.11 0.109 ...
  .. ..$ conint  : num [1:501] 0.226 0.223 0.221 0.218 0.217 ...
  .. ..$ all_ind : num [1:501] -1000 -995 -990 -985 -980 -975 -970 -965 -960 -955 ...
  .. ..$ e       : NULL
  .. ..$ desc    : chr "HTZ1_JA00001_IL1andIL2...\n@HTZ1_Differential_genes_TOP100_v2"
  .. ..$ heatmap : num [1:100, 1:501] 2.36 5.25 2.2 3.48 4.32 ...
  ..$ HTZ1_JA00001_IL3andIIL5_F_lin35_L3_NORM_linear_1bp_IL008andIL011_averaged.bw:List of 7
  .. ..$ means   : num [1:501] 2.36 2.35 2.35 2.36 2.38 ...
  .. ..$ stderror: num [1:501] 0.126 0.125 0.125 0.126 0.125 ...
  .. ..$ conint  : num [1:501] 0.249 0.249 0.247 0.251 0.249 ...
  .. ..$ all_ind : num [1:501] -1000 -995 -990 -985 -980 -975 -970 -965 -960 -955 ...
  .. ..$ e       : NULL
  .. ..$ desc    : chr "HTZ1_JA00001_IL3andIIL5...\n@HTZ1_Differential_genes_TOP100_v2"
  .. ..$ heatmap : num [1:100, 1:501] 2.61 3.17 1.42 2.46 4.26 ...
 $ HTZ1_Differential_genes_BOTTOM100.gff:List of 2
  ..$ HTZ1_JA00001_IL1andIL2_F_N2_L3_NORM_linear_1bp_IL010andIL009_averaged.bw    :List of 7
  .. ..$ means   : num [1:501] 1.57 1.57 1.58 1.6 1.62 ...
  .. ..$ stderror: num [1:501] 0.0996 0.0985 0.1003 0.1022 0.1018 ...
  .. ..$ conint  : num [1:501] 0.198 0.195 0.199 0.203 0.202 ...
  .. ..$ all_ind : num [1:501] -1000 -995 -990 -985 -980 -975 -970 -965 -960 -955 ...
  .. ..$ e       : NULL
  .. ..$ desc    : chr "HTZ1_JA00001_IL1andIL2...n@HTZ1_Differential_genes_BOTTOM100"
  .. ..$ heatmap : num [1:100, 1:501] 1.64 1.37 1.61 1.77 1.86 ...
  ..$ HTZ1_JA00001_IL3andIIL5_F_lin35_L3_NORM_linear_1bp_IL008andIL011_averaged.bw:List of 7
  .. ..$ means   : num [1:501] 1.94 1.94 1.95 1.96 1.97 ...
  .. ..$ stderror: num [1:501] 0.123 0.123 0.124 0.126 0.128 ...
  .. ..$ conint  : num [1:501] 0.244 0.245 0.246 0.251 0.253 ...
  .. ..$ all_ind : num [1:501] -1000 -995 -990 -985 -980 -975 -970 -965 -960 -955 ...
  .. ..$ e       : NULL
  .. ..$ desc    : chr "HTZ1_JA00001_IL3andIIL5...\n@HTZ1_Differential_genes_BOTTOM100"
  .. ..$ heatmap : num [1:100, 1:501] 1.61 1.37 1.29 3.04 3.77 ...
```

# Advanced options

Some additional SeqPlots options are located at very bottom of the `Batch operation and setup` tab:


- `Keep 1:1 aspect ratio in batch mode` - This option guarantee that the ratio between X- and Y-axis height will be 1, hence the produced plots will be square in batch mode. This prevents stretching the plots while fitting single rows or columns to one page. Turned on by default.
- `Always keep 1:1 aspect ratio` - This checkbox extends the 1:1 aspect ratio option to single plots. Turned off by default.
- `Reactive plotting` - When selected, all plotting operations are executed upon selection and will be visible in preview. `Reactive plotting` might be useful for exploratory data analysis using plots, but it is not recommended for heatmap plots because speed is decreased. Select/delelect from keyboard by pressing [ctrl/cmd+R]. Turned off by default.
- `Use multithreading for calculations` - This option is available only on desktop instances of SeqPlots under Mac OS X and Linux. While turned off R will not fork the child processes for plotting and plot set calculations. It is useful for debugging, since in single process mode all warning/errors will be directly printed to R console. Also might increase the performance for plotting small average plots. Turned off by default.
- `Use ggplot2 graphics package for heatmaps` - uses GGplot2 (http://ggplot2.org/) graphics system to draw the heatmaps. This feature is experimental.

# Genomes managment

The `Manage reference genomes` tab allows to add or remove reference genomes installed with SeqPlots. New genomes are added to user data folder, so they can be easily moved along with data and would not be removed when updating R or SeqPlots. User can choose to install standard genomes package available with Bioconductor or install forged genomic package from local file.

# Error messages

```
Problem with line N: "line_text" [internal_error]
```

> The import of feature file (GFF or BED) was not successful due to mis-formatted file.

---

```
Chromosome names provided in the file does not match ones defined in reference genome. 
INPUT: [chr3R, chr2L, chr2R, chr3L] 
GENOME: [chrI, chrII, chrIII, chrIV, chrV, ...]
```

> There are unexpected chromosome names in input file. Following genomes: *Arabidopsis thaliana, Caenorhabditis elegans, Cyanidioschyzon\_merolae, Drosophila melanogaster, Homo sapiens, Oryza sativa, Populus trichocarpa, Saccharomyces cerevisiae and Zea mays* support chromosome names remapping between different naming conventions, including: AGPv2, ASM9120v1, Ensembl, JGI2\_0, MSU6, NCBI, TAIR10 and UCSC. If you see above error in one of these genomes there are still unexpected names after the correction. The problematic chromosome names are given in the error message. Remove GFF/BED lines corresponding to them or upgrade the genome to one containing proper naming. Alternatively set genome to NA.

---

```
File already exists, change the name or remove old one.
```

> File named like this already exists in the database, it is impossible to have two files sharing same filename.

---

```
ERROR: solving row 300: negative widths are not allowed
```

> The the row 300 have end coordinate smaller than beginning, hence the width in negative. To fix it the start and stop indicates should be swapped. This error often happens when negative strand (-) ranges are misformatted.

---

# Explanations

---

- “**feature**” - a genomic interval defined by **chromosome** name, **start** and **end** positions and the **directionality** (strand). The end must always be a bigger number than start, so the width of the range is not negative. Start and end means here the numeric start of the interval and should not be confused with TSS and TTS.

  For example, in BED format this information is stored in following text tab delimited format: `chr7 127471196 127472363 . . +`

  ---
- “**directionality**” - the strand of genomic feature, determining if the plotting range should be anchored around the star or and, and the direction in which signal is being processed to create the average track or heatmap. Unknown directionality is marked by `*` and treated as `+` for calculations.

  ---
- “**track**” - the file assigning the continuous signal (score) to genomic locations across the chromosomes. The signal usually comes from sequencing experiments, like ChIP-seq, RNA-seq, DNase-seq, MNase-seq, or from computational tools, for example nucleosome occupancy prediction, CpG density.

  For example, in BedGraph format this information is stored in following text tab delimited format: `chr19 49302300 49302600 -0.75`

  ---
- “**reference genome package**” - the R BSgemome package containing the full reference sequence for given species. It is also used to provide universal chromosome names and chromosome lengths taht are used as plotting boundaries.

  ---
- “**reads coverage**” - The basic way to calculate the signal from sequencing based assays. The numeric representation shows how much reads was aligned to given genomic location. This can be a proxy to protein-DNA binding (ChIP-seq) or the expression (RNA-seq). Can be calculated using BedTools: http://bedtools.readthedocs.org/en/latest/content/tools/genomecov.html Also known as `pileups`.

  ---

# References

> This section lists R, JavaScript and CSS libraries used by SeqPlots, important conceptual contributions to the software, and publication, where figures generated by SeqPlots are featured.

**R project and Bioconductor**

- R Core Team (2014). R: A language and environment for statistical computing. R Foundation for Statistical Computing, Vienna, Austria. URL http://www.R-project.org/.
- Bioconductor: Open software development for computational biology and bioinformatics R. Gentleman, V. J. Carey, D. M. Bates, B.Bolstad, M.Dettling, S. Dudoit, B. Ellis, L. Gautier, Y. Ge, and others 2004, Genome Biology, Vol. 5, R80. URL http://www.bioconductor.org/.
- RStudio and Inc. (2014). shiny: Web Application Framework for R. R package version 0.10.1. http://shiny.rstudio.com/
- **Other CRAN packages:** digest, DBI,RSQLite, RJSONIO, plotrix, fields, grid, kohonen, ggplot2, Cairo and parallel
- **Bioconductor packages:** IRanges, BSgenome, Rsamtools, rtracklayer, GenomicRanges and Biostrings

**JavaScript and CSS**

- jQuery framework - http://jquery.com
- Bootstrap - http://getbootstrap.com
- DataTables, Table plug-in for jQuery - http://www.datatables.net
- jQuery File Upload Plugin - https://github.com/blueimp/jQuery-File-Upload
- jQuery throttle - http://benalman.com/projects/jquery-throttle-debounce-plugin/
- jQuery Cookie Plugin - https://github.com/carhartl/jquery-cookie
- Modernizer JS library - http://modernizr.com
- JavaScript Templates - https://github.com/blueimp/JavaScript-Templates
- JavaScript Color Picker - http://jscolor.com
- md5-js - https://github.com/wbond/md5-js
- Font Awesome - http://fortawesome.github.io/Font-Awesome
- Google Fonts - https://www.google.com/fonts
- jQuery user interface - http://jqueryui.com (documentation)
- jquery.tocify.js: jQuery Table of Contents - https://github.com/gfranko/jquery.tocify.js (documentation)
- Strapdown https://github.com/arturadib/strapdown (documentation)
- Bootswatch themes - http://bootswatch.com (documentation)
- google-code-prettify - https://code.google.com/p/google-code-prettify (documentation)
- marked - https://github.com/chjj/marked (documentation)

**Important conceptual contribution to the project**

- Liu T, Ortiz J, Taing L, Meyer C, Lee B, Zhang Y, Shin H, Wong S, Ma J, Lei Y, et al. 2011. Cistrome: an integrative platform for transcriptional regulation studies. Genome Biology 12: R83.
- Thomas Williams, Colin Kelley and others (2010). Gnuplot 4.4: an interactive plotting program. URL http://www.R-project.org/.
- Kent, W.J., Sugnet, C.W., Furey, T.S., Roskin, K.M., Pringle, T.H., Zahler, A.M. and Haussler, a. D. (2002). The Human Genome Browser at UCSC. Genome Research. 12:996–1006.
- Kent WJ, Zweig AS, Barber G, Hinrichs AS, Karolchik D. (2010). BigWig and BigBed: enabling browsing of large distributed datasets. Bioinformatics. 1;26(17):2204-7
- Nicol, J.W., Helt, G.A., Blanchard, S.G., Raja, A. and Loraine, A.E. (2009). The Integrated Genome Browser: free software for distribution and exploration of genome-scale datasets. Bioinformatics (Oxford, England). 25:2730–1.
- Thorvaldsdóttir, H., Robinson, J.T. and Mesirov, J.P. (2012). Integrative Genomics Viewer (IGV): high-performance genomics data visualization and exploration. Briefings in bioinformatics. bbs017

**Server deployment**

- Shiny Server - https://github.com/rstudio/shiny-server
- ShinyApps - https://github.com/rstudio/shinyapps

**Publications containing figures made by SeqPlots**

- Chen RA, Stempor P, Down TA, Zeiser E, Feuer SK, Ahringer J. Extreme HOT regions are CpG-dense promoters in C. elegans and humans. Genome Res 24(7):1138-1146 Jul 2014
- Latorre I, Chesney MA, Garrigues JM, Stempor P et al. The DREAM complex promotes gene body H2A.Z for target repression. Genes Dev 2015 Mar 1;29(5):495-500.

# Session Information

```
## R version 3.2.2 (2015-08-14)
## Platform: x86_64-apple-darwin13.4.0 (64-bit)
## Running under: OS X 10.10.2 (Yosemite)
## 
## locale:
## [1] en_US.UTF-8/en_US.UTF-8/en_US.UTF-8/C/en_US.UTF-8/en_US.UTF-8
## 
## attached base packages:
## [1] stats4    stats     graphics  grDevices utils     datasets  methods  
## [8] base     
## 
## other attached packages:
##  [1] seqplots_1.7.2                    ggplot2_1.0.1                    
##  [3] BSgenome.Celegans.UCSC.ce10_1.4.0 BSgenome_1.36.3                  
##  [5] rtracklayer_1.28.10               Biostrings_2.36.4                
##  [7] XVector_0.8.0                     GenomicRanges_1.20.6             
##  [9] GenomeInfoDb_1.4.2                IRanges_2.2.7                    
## [11] S4Vectors_0.6.5                   testthat_0.10.0                  
## [13] BiocGenerics_0.14.0               devtools_1.8.0                   
## 
## loaded via a namespace (and not attached):
##  [1] Rcpp_0.12.0             Rsamtools_1.20.4       
##  [3] class_7.3-14            digest_0.6.8           
##  [5] mime_0.4                R6_2.1.1               
##  [7] plyr_1.8.3              futile.options_1.0.0   
##  [9] evaluate_0.7.2          RSQLite_1.0.0          
## [11] spam_1.0-1              httr_1.0.0             
## [13] zlibbioc_1.14.0         lazyeval_0.1.10        
## [15] curl_0.9.3              rstudioapi_0.3.1       
## [17] DT_0.1.31               rmarkdown_0.8          
## [19] proto_0.3-10            BiocParallel_1.2.21    
## [21] stringr_1.0.0           htmlwidgets_0.5        
## [23] RCurl_1.95-4.7          munsell_0.4.2          
## [25] shiny_0.12.2            httpuv_1.3.3           
## [27] rversions_1.0.2         htmltools_0.2.6        
## [29] gridExtra_2.0.0         roxygen2_4.1.1         
## [31] codetools_0.2-14        XML_3.98-1.3           
## [33] crayon_1.3.1            GenomicAlignments_1.4.1
## [35] MASS_7.3-44             bitops_1.0-6           
## [37] grid_3.2.2              jsonlite_0.9.17        
## [39] xtable_1.7-4            gtable_0.1.2           
## [41] DBI_0.3.1               formatR_1.2            
## [43] git2r_0.11.0            magrittr_1.5           
## [45] covr_1.2.0              scales_0.3.0           
## [47] stringi_0.5-5           kohonen_2.0.19         
## [49] reshape2_1.4.1          rex_1.0.1              
## [51] xml2_0.1.2              futile.logger_1.4.1    
## [53] BiocStyle_1.6.0         lambda.r_1.1.7         
## [55] RColorBrewer_1.1-2      tools_3.2.2            
## [57] Cairo_1.5-8             maps_2.3-11            
## [59] fields_8.2-1            yaml_2.1.13            
## [61] plotrix_3.5-12          parallel_3.2.2         
## [63] colorspace_1.2-6        memoise_0.2.1          
## [65] knitr_1.11
```
