# Supplementary material for: McClintock: An Integrated Pipeline for Detecting Transposable Element Insertions in Whole-Genome Shotgun Sequencing Data
Source: G3 (Bethesda). 2017 Jun 21;7(8):2763–78. doi: 10.1534/g3.117.043893 (PMC5555480; doi:10.1534/g3.117.043893)
Supplement: Supplementary file 3 [file 2763FileS3.zip › mcclintock_analysis_code/seqplots-master/inst/seqplots/www/help/help.html]

SeqPlots


Toggle navigation

- Bioconductor
- GitHub
- Known issues
- Report an issue


# 

- Welcome to **SeqPlots**
  - Summary
  - Availability
  - Key features
  - Video presentation
  - Issues and bugs
- Installation - Mac OS X app
  - How to install
  - How to start
  - Package content
- Installation - R/Bioconductor
  - How to install
  - How to start
- Installation - Server deployment
  - Installation
  - Usage
  - Alternative installation
- Installation - Docker image
  - How to install Docker
  - How to use on Mac and Windows
  - How to use Linux
  - Troubleshooting
  - Package content
- Quick start demo
- Adding and managing files
  - Supported file formats
  - Adding files
  - Downloading and removing files
- Running the plot-set jobs
  - Selecting files
  - Setting up plot options
  - Plotting sequence motif density
  - Starting the plot set calculation
- Plotting
  - Previewing plot
  - Titles and axis panel
  - Guide lines and data scaling
  - Keys, labels and colors panel
- Heatmaps
  - Heatmap setup tab
  - Other options controlling heatmap appearance
- Getting PDFs and cluster info
  - PDF output size
- Batch operations
- Saving and loading plotsets
  - Load or save plotset
  - Plot set files structure
- Advanced options
- Genomes managment
- Error messages
- Versions history
  - CHANGES IN VERSION 1.4.1
  - CHANGES IN VERSION 1.3.0
  - CHANGES IN VERSION 1.2.0
  - CHANGES IN VERSION 1.0.0
  - CHANGES IN VERSION 0.99.1
  - CHANGES IN VERSION 0.99
  - CHANGES IN VERSION 0.9.3
  - CHANGES IN VERSION 0.9.2
  - CHANGES IN VERSION 0.9.1
  - CHANGES IN VERSION 0.9.0
  - CHANGES IN VERSION 0.8.2
  - CHANGES IN VERSION 0.8.1
  - CHANGES IN VERSION 0.8.0
  - CHANGES IN VERSION 0.7.0
  - CHANGES IN VERSION 0.6.0
  - CHANGES IN VERSION 0.5.0
- Explanations
- References


---

# Welcome to **SeqPlots**

> An interactive tool for visualizing NGS signals and sequence motif densities along genomic features using average plots and heatmaps.

## Summary

SeqPlots is a web browser tool for plotting average track signals (e.g. read coverage) and sequence motif densities over user specified genomic features. The data can be visualized in linear plots with error estimates or as series of heatmaps that can be sorted and clustered. The software can be run locally on a desktop or deployed on a server and allows easy data sharing. SeqPlots pre-calculates and stores binary result matrices, allowing rapid plot generation. Plots can also be run in batch.

## Availability

SeqPlots is distributed as Bioconductor package available on platforms and operating systems supported by R project. A standalone SeqPlot OS X bundle, combing R and all required packages, is available as for Mac OS X (10.6 or higher). SeqPlots can also be deployed on a server using free and open sourced (GPL licensed) Shiny Server.

## Key features

- Easy to use web interface (R or shell expertise not required)
- Web server or desktop versions
- Generates publication quality plots out of the box
- Plots average signals or heatmaps
- Accepts Wiggle, BedGraph, BigWiggle, and GFF and BED formats
- Calculates motif density from reference genome packages
- Tracks and features are searchable and old calculations stored
- Converts tracks to binary BigWiggle format for rapid data extraction and efficient storage
- Implemented using Shiny R framework providing internet browser reactive GUI and session based connectivity (websocets)

## Video presentation

## Issues and bugs

Please visit **issue tracker** to view currently know issues. To report a new issue/bug/feature request please click **here**. If the issue is connected to file upload please attach the file in the form.

---

# Installation - Mac OS X app

> The Mac OS X bundle is an easy way to use SeqPlots for Mac OS X users. It contains all R binaries and packages that SeqPlots depends on. Additionally, test tracks and genome annotations, and reference sequences for *Homo sapiens*, *Drosophila melanogaster* and *Caenorhabditis elegans* are included. Sequences for other organisms can be downloaded using a graphical user interface.

**System requirements**

- Mac OS X 10.6 (Snow Leopard) or higher

## How to install

1. Download the compressed application from https://github.com/Przemol/seqplots\_osx/releases/latest
2. Double-click on downloaded file to unzip the contents of archive
3. Drag and drop SeqPlots.app to Applications folder

## How to start

Start SeqPlots from Applications. If run for the *first time* the operating system security notice might be shown.


To run the application right-click on SeqPlots icon and choose “Open”, followed by clicking “Open” button in popup window. This need to be done only once, later SeqPlots can be started as usual Mac OS X application.


After starting SeqPlots the welcome screen displays the software version, the currently installed genomes and the data folder location.


This screen allows you to set up following options:

- Change your data location folder - by default your home directory will be used (**`Change data location`** button)
- Exit application (**`Quit`** button)

Press the **`START`** button to initiate SeqPlots. If initiation was successful the user interface should open in your default web browser.

For SeqPlots usage instructions please refer to **Quick start demo** or specific chapters of this document.

The window in the background allows you to assess if SeqPlots is running properly and exit the application at any moment.


The full progress bar indicates that SeqPlots is running. Press “Cancel” to stop it. Pressing “Details” will reveal a small text window that displays diagnostic and error messages.

## Package content

- Platypus (http://www.sveinbjorn.org/platypus) wrapper
- R (http://www.r-project.org/) branch 3.1 for Snow leopard (http://r.research.att.com/snowleopard/R-3.1-branch/R-3.1-branch-snowleopard-sa-x86\_64.tar.gz)
- SeqPlots dependency packages (including Shiny, rtracklayer and BSgenome)
- Full genome sequences for Caenorhabditis elegans (UCSC version ce10) - BSgenome.Celegans.UCSC.ce10 R package
- SeqPlots package

---

# Installation - R/Bioconductor

> The Bioconductor package runs SeqPlots locally on desktop computers from the R environment. It is available for Windows, GNU/Linux and Mac OS X operating systems. For further information please refer to SeqPlots Bioconductor webpage.

**System requirements:**

- R 3.1 or higher

## How to install

To install SeqPlots package, start R and enter:

```
source("http://bioconductor.org/biocLite.R")
          biocLite("seqplots")
```

## How to start

To start SeqPlots web interface, start R and enter:

```
library(seqplots)
          run()
```

After a successful initiation the user interface will be opened in your default web browser. For further usage please refer to **Quick start guide** or specific chapters.

The `run` function accepts “root” argument, which allows to change the data location folder (by default your home directory will be used), e.g.:

```
run(root='/path/to/data/location')
```

### Additional genome packages

Genomic packages can be installed using standard bioconductor installer (Internet connection is required). For example, to instal human reference genome (hg19):

```
source("http://bioconductor.org/biocLite.R")
          biocLite("BSgenome.Hsapiens.UCSC.hg19")
```

Genome packages are required before uploading files for plotting. Full list of supported genomes is avilable here: http://www.bioconductor.org/packages/release/BiocViews.html#\_\_\_BSgenome

---

# Installation - Server deployment

> The server version allows the sharing of signal and feature files by a group of users and for remote calculation and file storage. This is the ideal method for providing a shared SeqPlots service to a group.

**System requirements:**

- R 3.1 or higher
- Shiny Server 1.0 or higher

## Installation

1. Install and configure the Shiny Server by following the instructions on https://github.com/rstudio/shiny-server

2. Install SeqPlots R package and dependences by following the instruction here

3. Copy SeqPlots files to Shiny Server application folder:

```
cp -r $(Rscript -e "cat(system.file('seqplots', package='seqplots'))") /srv/shiny-server/
```

4. Set up SeqPlots **data location** by running from R:

```
seqplots(root='/path/to/data/location')
```

5. Edit first line of `/srv/shiny-server/shiny/server_config.R`, so the environment variable `root` matches the **data location**; for example:

```
Sys.setenv('root'='/var/shiny-server/DATA')
```

## Usage

After successful installation the SeqPlost web GUI will be available at `your_server_name:3838/seqplots/`.

For further usage please refer to quick start guide or specific chapters of documentation.

### Additional genome packages

Genomic packages can be installed using standard bioconductor installer (Internet connection required). For example, to instal human reference genome (hg19):

```
source("http://bioconductor.org/biocLite.R")
          biocLite("BSgenome.Hsapiens.UCSC.hg19")
```

Corresponding genome packages are required before uploading the files for plotting. Full list of supported genomes is avilable here: http://www.bioconductor.org/packages/release/BiocViews.html#\_\_\_BSgenome

## Alternative installation

SeqPlots can be directly cloned into Shiny Server application folder from git repository by using `git clone https://github.com/Przemol/seqplots.git`. The data location can be set up with following R code:

```
root <- "/data/location"
          dir.create(root)
          setwd(root)
          require(RSQLite)
          sqlite <- dbDriver("SQLite")
          con <- dbConnect(sqlite, dbname = "files.sqlite")
          dbGetQuery(con, "CREATE TABLE files (id INTEGER PRIMARY KEY ASC, name TEXT UNIQUE, 
            ctime TEXT, type TEXT, format TEXT, genome TEXT, user TEXT, comment TEXT)")
          if (!dbListTables(con) == "files") 
              warning("Database not created!")
          dbDisconnect(con)
          if (!all(sapply(c("removedFiles", "files", "publicFiles", "tmp"), dir.create))) 
              warning("Folders not created!")
          message("\nData loaction: ", root)
```

The following dependencies must be installed in R:

```
              package  version
          1              shiny   0.10.0
          2             fields      7.1
          3           parallel    3.1.0
          4          multicore      0.2
          5           BSgenome   1.32.0
          6      GenomicRanges   1.16.3
          7            plotrix    3.5-7
          8        rtracklayer   1.24.2
          9            RJSONIO  1.2-0.2
          10           RSQLite   0.11.4
          11           kohonen   2.0.14
          12             Cairo    1.5-5
          13            digest    0.6.4
          14           methods    3.1.0
          15             tools    3.1.0
          16             utils    3.1.0
          17            httpuv    1.3.0
          18           caTools     1.17
          19            xtable    1.7-3
          20         htmltools    0.2.4
          21            bitops    1.0-6
          22              Rcpp   0.11.2
          23              spam   0.41-0
          24              maps    2.3-7
          25              grid    3.1.0
          26         grDevices    3.1.0
          27      BiocGenerics   0.10.0
          28           IRanges   1.22.9
          29        Biostrings   2.32.0
          30           XVector    0.4.0
          31         Rsamtools   1.16.1
          32          graphics    3.1.0
          33             stats    3.1.0
          34          zlibbioc   1.10.0
          35      GenomeInfoDb    1.0.2
          36            stats4    3.1.0
          37               XML 3.98-1.1
          38             RCurl 1.95-4.1
          39 GenomicAlignments    1.0.1
          40      BiocParallel    0.6.1
          41           foreach    1.4.2
          42         BatchJobs      1.2
          43            BBmisc      1.7
          44               DBI    0.2-7
          45         sendmailR    1.1-2
          46              brew    1.0-6
          47              plyr    1.8.1
          48           stringr    0.6.2
          49              fail      1.2
          50         checkmate      1.0
          51         codetools    0.2-8
          52         iterators    1.0.7
          53         base64enc    0.1-1
          54             class   7.3-10
          55              MASS   7.3-33
```

---

# Installation - Docker image

> SeqPlots is available as Docker image, which can be easily run on Mac OS, Windows and Linux systems. The image can be obtained from Docker Hub: https://hub.docker.com/r/przemol/seqplots/

**System requirements**: Windows, Mac OS X, Linux or any other system supporting the Docker software. Refer to https://www.docker.com/ for details.

## How to install Docker

Install the Docker Toolbox/Docker executables:

- Windows: http://docs.docker.com/windows/step\_one
- Mac OS X: http://docs.docker.com/mac/step\_one
- Linux: http://docs.docker.com/linux/step\_one

## How to use on Mac and Windows

1. On Mac or Windows start Kitematic. The application will be installed as a part of Docker Toolbox. You may skip the registration/login.
2. Search for “seqplots”, then click on `CREATE` button. This will download the container, all it’s dependencies and run the application. The SeqPlots web interface should be shown in “WEB PREVIEW” window on right side of Kitematic.
3. To open SeqPlots interface click on `WEB PREVIEW`. This will open the GUI is system default web browser. At this point the SeqPlots is ready to use.
4. The uploaded files, datasets and downloaded reference genomes will be saved inside the Docker container. To use local system storage or access files previously added using SeqPlots Mac OS X app or R package click on the cog icon “VOLUMES” section of Kitematic (below `WEB PREVIEW`). There should be one Docker folder (/var/shiny-server/DATA), currently not mapped to local filesystem. Click on `CHANGE` button on the right side and select your data location, e.g. ~/SeqPlots\_data. Verify that path is correct in `LOCAL FOLDER` column, restart SeqPlots Docker image if needed.

## How to use Linux

1. After installing Docker and verifying it works properly (`docker run hello-world`) run following commands in terminal:

   ```
   docker pull przemol/seqplots
             docker run -p 80:80 przemol/seqplots
   ```
2. At this point the SeqPlots should be available on port 80 of local web interface. Open the web browser and navigate to http://localhost.

   If port 80 is already taken (e.g. by Apache web server), change port mapping in docker command, e.g. `docker run --rm -p 3838:80 przemol/seqplots` and navigate to http://localhost:3838.
3. To use local file system for SeqPlots data storage use `-v <host_dir>:<container_dir>` parameter:

   ```
   docker run -p 3838:80 -v ~/SeqPlots_data:/var/shiny-server/DATA przemol/seqplots
   ```

## Troubleshooting

When using shared folder error messages `The application unexpectedly exited.` or `Error in plot.new() : could not open file 'tmp/sessionID_1b61fb3846.png'` indicate that shared file system is not writeable for SeqPlots in Docker container. Make sure all files and folders have “Read & Write” permission set for everyone (all users). This can be changed using right click followed by “More info” (Mac) or “Properties” (Windows). On Mac and Linux the following command will fix all issues:

```
chmod -R a+w ~/SeqPlots_data
```

## Package content

- R (http://www.r-project.org/) branch 3.2 for Linux
- Shiny Server (https://github.com/rstudio/shiny-server)
- SeqPlots dependency packages (including Shiny, rtracklayer and BSgenome)
- Full genome sequences for Caenorhabditis elegans (UCSC version ce10) - BSgenome.Celegans.UCSC.ce10 R package
- SeqPlots package

---

# Quick start demo

1. Start SeqPlots. Refer to **installation guides** for platform specific information. After successful initiation the web interface should automatically open in your default web browser. If you are using web server version just navigate your browser to the server address.
2. Upload feature (BED or GFF) and track (BigWig or WIG) files. They can be gzip compressed (e.g. file1.bed.gz). Press green “Add files…” button or just drag and drop files into the window. The ready to upload files will show up in upload window, where you select user name, reference genome and optionally add some comments.
3. When all is done press blue “Start upload” button. After upload and processing is done the green “SUCCESS” label should show. It means that file is on the registered and ready to use. Occasionally the file might be mot formatted properly or chromosome names might not agree with reference genome. In such case a verbose error will window appears and file as labeled as “ERROR”. For further information please refer to **errors chapter**.
4. Dismiss upload window and press blue “New plot set” button on side panel. This will bring up file management window. In file management window select at least one file from “Features” tab and at least one file from “Tracks” or sequence motif(s). The sequence motifs and tracks can be processed and plotted together. Select files by clicking on file name, selected files will be highlighted.
5. After choosing files/motifs to plot, set up the processing options. You can find these in the button of plotting window.
6. After options are set up press blue “Run calculation” button. This will dismiss the file management window and show processing message. Here you can observe the progress of the task and optionally cancel it if no longer required or you forgot to add some important file to the plot-set.
7. After some time the calculation will finish (fingers crossed, without the error) and you will be able to see plot set array. In here you can choose which feature-track or feature-motif pairs to plot. Choose one or more checkboxes and press grey “Line plot” button (or hit RETURN from your keyboard).
8. Congratulation! Your First plot is complete, you can see the preview of it on the side panel.
9. You are able to set up labels, titles, font sizes, legends and many more on control panel tabs - please see **Plotting** chapter for details.
10. By clicking the plot preview you can enlarge it for better view. When everything is ready you can get the plot as PDF by clicking green “Line plot” button just on the top of side panel.
11. You can also visualize the signal as a heatmap. Please note that heatmap plotting is possible only for a single feature file. It is possible to sort heatmaps based on mean row signals and/or cluster them using k-means algorithm, hierarchical clustering or self organising maps. To learn more about heatmaps see **Heatmaps** chapter.
12. Heatmaps can be downloaded sa PDFs using ‘Heatmap’ button just on the bottom of setup panel. The small button, at the right sied of ‘Heatmap’ button downloads cluster definition and sorting order.

---

# Adding and managing files

## Supported file formats

Tracks:

- BigWig (.bw) - http://genome.ucsc.edu/FAQ/FAQformat.html#format6.1
- Wiggle (.wig) - http://genome.ucsc.edu/goldenPath/help/wiggle.html
- BedGraph (with .wig extension) - http://genome.ucsc.edu/goldenPath/help/bedgraph.html

Features:

- BED - http://genome.ucsc.edu/FAQ/FAQformat.html#format1
- GFF - http://genome.ucsc.edu/FAQ/FAQformat.html#format3
- GTF (with .gff extension) - http://genome.ucsc.edu/FAQ/FAQformat.html#format4

Files must be formatted according to UCSC guidelines. All widely used chromosome names conventions are accepted, e.g. for human files either ‘chr1’ or ‘1’ can be used, however these conventions should not be mixed within single files.

## Adding files

Press the `Add files` button to bring up the **file upload panel**.


You can drag and drop files here or press the `Add files...` button to open a file selection menu. Before starting the upload the following mandatory information must be provided about each file:

- User ID
- Reference genome - drop-down menu containing reference genome package currently installed in R

Comments are optional.

The contents of the text field can be copied to all files by clicking the icon at the left of the field. The default values can be set using `Set defaults...` button. Default values are stored using the browser cookies, and the settings will be remembered across different sessions as long as the same web browser is used. File extensions that are not supported will raise an error.


Individual files can be uploaded by pressing ‘start’ next to the file name or all files can be uploaded at once by pressing the `Start upload` button at the top of **file upload panel**.

During the upload process a progress bar is displayed. After upload SeqPlots gives a message that upload was successful or or gives an error message. Common errors are misformatted file formats or chromosome names do not matched the reference genome. For more information please refer to *Error explained* chapter.


To dismiss the upload window, click on `X` or outside the window.

## Downloading and removing files

Clicking the `New plot set` button brings up the **file collection window**. The primary function of this window is to choose signal tracks and feature files to use for calculating the plots. However, it also provides basic file management capabilities. Information on files can be reviewed and files can be downloaded or deleted. Fields can be searched, filtered and sorted by any column. The red `x` button on the right site of file table removes a single file from the collection, while `Remove selected files` button will erase all selected files.


---

# Running the plot-set jobs

Pressing the `New plot set` button brings up the **file collection window** from which you can choose signal tracks and feature files to calculate average plots and heat maps. If you wish to upload more files please refer to adding new files documentation. The **file collection window** has three tabs:

- `Tracks` - signal files, i.e., Wiggle, BigWiggle and BedGraph files.
- `Features` - genomic feature files, i.e., BED, GFF and GTF files
- `Sequence features` - input any motif of interest that you want to plot.


## Selecting files

The `Tracks` and `Features` tabs displays information about the files and allows you to filter and sort by any column. The “Search:” dialog allows you to find any keyword in any field, while dropdowns below the file grid allow for more advanced filtering on specific columns.

Select files by clicking on the file name or any other part of the row beside `Show comment` and `Download` or `Remove` buttons. Chosen files are highlighted in light blue. Clicking the file name again will cancel the selection. At least one signal track or motif and one feature file must be selected before starting the calculation.

## Setting up plot options

Options controlling the plot settings is found below the file selection window:

1. **`Bin track @ [bp]:`** - this numeric input determines the resolution of data acquisition; the default value 10 means that 10bp intervals within the plotting range will be summarized by calculating the mean. Higher values increases the speed of calculation, but decreases resolution. See the explanations.
2. **`Choose the plot type`** - there are three options:
   - *`Point Features`* - anchor plot on the start of a feature. By default, plot will be directional if strand information is present (i.e, use start position and plot on positive strand for + strand features and use end position and plot on negative strand for minus strand features). If strand information is not present in the feature file (or if the “ignore strand” option is chosen), plot will use start position of feature and be plotted on the positive strand (see explanations). User chooses length of upstream and downstream sequence to plot.
   - *`Midpoint Features`* - similar to point feature, but plot is centered on the midpoint of the feature.
   - *`Endpoint Features`* - similar to point feature, but plot is centered on the end of the feature. Strand information is used by default to determine the end side.
   - *`Anchored Features`* - features are anchored at start and stop positions and given pseudo-length chosen by the user. Additionally, the user chooses the length of sequence upstream of the start and downstream of the end to plot.
3. **`Ignore strand`** - the directionality (strand) will be ignored all features plotted on the positive strand.
4. **`Ignore zeros`** - signal values of 0 in the track will be be excluded from calculations
5. **`Calculate heatmap`** - selecting this generates and saves a heat map matrix. Select if you wish to generate heatmap; uncheck if you only wish to generate average plots, as this will speed calculations.
6. **`Plotting distances in [bp]`** - the distances in to be plotted:
   - *`Upstream`* - the plotting distance in base pairs upstream to the feature
   - *`Anchored`* - the pseudo-length, to which the features will be extended or shrunk using linear approximation (only for anchored plots)
   - *`Downstream`* - the plotting distance in base pairs downstream to the feature

## Plotting sequence motif density

The `Sequence features` tab allows you to calculate and plot the density of any user-defined motif around the chosen genomic feature using the reference sequence package. Motif plots can be mixed with track files’ signal plots. The following options can be set:

1. **`DNA motif`** - the DNA motif
2. **`Sliding window size in base pairs [bp]`** - the size of the sliding window for motif calculation. The value (number of matching motifs within the window) is reported in the middle of the window, e.g. if window is set to 200bp, DNA motif is “GC” and there are 8 CpGs in first 200 bp of the chromosome the value 8 will be reported at 100th bp.
3. **`Display name`** - The name of the motif that will be shown in key and heatmap labels. Leave blank to use `DNA motif` value.
4. **`Plot heatmap or error estimates`** - this checkbox determines if heatmap matrix and error estimates should be calculated. If unchecked much faster algorithm will be used for motif density calculation, but only the average plot without the error estimates will be available.
5. **`Match reverse complement as well`** - select if reverse complement motif should be reported as well. For example the TATA motif will report both TATA and ATAT with this option selected.


Clicking `Add` button adds the motif to plot set, while `Reset All` clears the motif selection. On the right side of the motif setting panel gives a list summary of included motifs.

## Starting the plot set calculation

The options are executed by pressing the `Run calculation` button. This dismisses the **file collection window** and brings up the calculation dialog, which shows the progress. On Linux and Mac OS X (systems supporting fork based parallelization) the calculation can be stopped using the `Cancel` button - this will bring back all settings in **file collection window**.


After successful execution the **plot array** and **plot preview panel** will appear. In case of error an informative error pop-up will explain the problem. Please refer to the error section for further information.


---

# Plotting

> This section focuses on average (line) plots and options common between these and heatmaps. For heatmap options please refer to heatmap documentation.

## Previewing plot

After calculating or loading a plot set, a **plot array** of checkboxes is displayed to select the desired pairs of features and tracks/motifs. Clicking on the column name (tracks/motifs) or row name (features) selects/deselects the whole column or row. Clicking on top-left most cell of **plot array** toggles the selection of whole array.


If at least one pair on **plot array** is selected pressing the `Line plot` button produces an average plot preview and the `Heatmap` button produces a heatmap preview. Alternatively, pressing the [RETURN] key will also produce the new selection and options. These operations are done automatically in reactive mode (see **Advanced options** chapter). Plots can be downloaded as PDF files using the Line plot and Heatmap buttons next to Download (at the top of the panel).

Below the plotting buttons are options for labeling plots and setting axes. On application start the first panel responsible for bringing file upload, management and plot set calculation modals is active. The further three panels hold common plot settings.

## Titles and axis panel


This panel groups settings influencing the plot main title, axis labels, various font sizes plus vertical and horizontal plot limits.

- `Title` - The main title of the plot, shown in top-center part of the figure; default empty
- `X-axis label` - Label shown below horizontal axis; default empty
- `Y-axis label` - Label shown below vertical axis; default empty
- `Title font size` - Font size of the title in points (point = ~1/72 an inch for standard A4 output); default 20 points
- `Labels font size` - Font size of axis labels in points; default 16 points
- `Axis font size` - Controls axis ticks font size, that is size of the numbers indicating position in base pairs on X-axis and means signal value on X-axis; default 14 points
- `Set X-axis limits` - Set hard plotting limits for X-axis; default values are whole range chosen during plot set calculation
- `Set Y-axis limits` - Set hard plotting limits for Y-axis; default values are a range between lowest and highest mean signal extended by error estimate

## Guide lines and data scaling


Controls in this panel controls the display of guide lines and error estimates, and allows to log scale the signal prior to plotting.

- `Transform signal` - if set to *`Log2 transform`* performs log2 transformation of the signal prior to plotting; default setting is *`Do not transform`*
- `Show vertical guide line` - show the vertical line at point 0 - beginning of the feature or midpoint and end of the pseudo-length scaled features (only for anchored plots); turn on by default
- `Show horizontal guide line` - show the horizontal line at user determined height; turn off by default
- `Show error estimates` - show error standard error and 95% confidence interval as fields, if turned off only the line representing the mean signal is shown; turn on by default

## Keys, labels and colors panel


This panel groups two types of controls. `Colors`, `Label` and `Priority/Order` are a checkboxes revealing further controls on **plot set grid**, specific for a feature-track pair or sub-heatmap. `Show plot key`, `Show error estimate key` and `Legend font size` re global controls specific for average plots. Inputs on **plot set grid** do not have specific labels, but the tooltip explaining their meaning is shown on mouse cursor hover.

- `Colors` - checkboxes revealing a color picker on **plot set grid**. This input allows to control the colors of specific feature-track pair average plots or sub-heatmaps. In browser supporting the color picker ‘e.g Chrome’ the system dialog will show up. In other browsers (e.g. Firefox) the javaScript color picker will be initialized.
- `Label` - checkboxes revealing a label text input **plot set grid**. This controls the names shown on the **key** with average plots or the heatmap top labels.
- `Priority/Order` - checkboxes revealing numeric input on **plot set grid**. These number determine the order of average plots and hetamaps. Feature-track pair with the highest priority will be listed on the top of **key** for average plots and left-most for heatmaps.
- `Show plot key` - shows the key giving the color to feature-track pair label mapping. If turned on the additional drop-down allows to choose the position on the plot, top-right by default
- `Show error estimate key` - shows the key gexplaining the meaning of error fields. If turnedon the additional drop-down allows to choose the position on the plot, top-left by default
- `Legend font size` - set the size of font used to plot the keys; 12 default

---

# Heatmaps

Heatmaps are often more informative than average plots. If there is variability in signal along individual instances of a given genomic feature (e.g., because there are different biological classes), an average plot might not represent the behavior of any individual feature and could even give a misleading picture. SeqPlots plots track-feature pairs as sub-heatmaps horizontally aligned on single figure. All sub-heatmaps must have the same number of data rows, hence in single plot mode simultaneous plotting is possible only on single features or feature files containing exact same number of rows. The heatmaps can be sorted and clustered by k-means, hierarchical clustering or super self organising maps (SupreSOM).

## Heatmap setup tab

This tab has heatmap specific options for data processing and display.


- **`Sort heatmap rows by mean signal`** - sorts the heatmap rows based on the mean value of each row across included sub-heatmps. Can be set to increasing or decreasing order. Turned off by default.
- **`Clustering algorithm`** - choose clustering algorithm (k-means, hierarchical or SupreSOM). If clustering is not desired, choose *`do not cluster`*, which uses the feature file in the uploaded order . K-means by default.
- **`Make cluster calculation repeatable`** - enforces, that clustering with non-deterministic algorithms, like k-means or SupreSOM will generate the same results as most recently plotted heatmap. This is achieved by re-using R random number generator seed.
- **`Plot selected cluster`** - this option is available only if `Make cluster calculation repeatable` is turned on. Allows to select one of the clusters and zoom it to whole plot height. Plot all clusters by default.
- **`Choose individual heatmaps for sorting/clustering`** - this checkbox brings up a new control panel on the **plot set grid** to determine if a given sub-heatmap should be included in plotting and/or clustering. The excluded sub-plots will be plotted in the order of the other sub-heatmaps, but their values will not influence the clustering/sorting. By default all sub-heatmaps are included.
- **`Heatmaps have individual color keys`** - by default all sub-heatmap have their own color keys. This option determines if each sub-heatmap should have a separate color key (plotted below the heatmap) or a single, common key should be calculated for all sub-plots (plotted rightmost). The example below show the difference between separate (left) and common (right) color keys:
- **`Set default color key limits`** - this option determines the limits in mapping the numerical values to the colors. The range of colors generated is dependent on these options. Values lower or higher than the given limits will be plotted in the limit value color. If this checkbox is not selected, limits are auto-generated using **`Color key scaling`** parameter. If this option it turned off two numerical fields, **`min`** and **`max`**, are shown to manually set the limits.

  - **`Color key scaling`** - this slider influences how color key limits are generated. For example, 0.01 (default value) calculates limits using data ranging from 1-99 percentile of available data points. 0.1 uses data ranging from 10-90 percentile. The general formula for limit is: [quantile(data, `Color key scaling`); quantile(data, 1-`Color key scaling`)]
  - **`min`** and **`max`** numeric inputs - enter values to manually specify color key limits as numeric values.
- **`Set individual color key limits`** - this option is similar to manual set up of color key limits, but this allows one to specify different values for individual sub-heatmaps. When this checkbox is selected **`min`** and **`max`** numeric input menu is shown on the **plot set grid**
- **`Set colorspace`** - This input allows to use color palettes from RColorBrewer package, which would replace default color palette for heatmaps. By selecting **`Reverse`** checkbox the reversed color palette will be used. Here are available color pallets (click here to see example heatmap plotted with different color palettes):

  When the `Custom` option is selected three-color pickers are shown to setup custom color mappings for heatmaps. The following example below shows standard jet colors (left), default blue color mapping after selecting the checkbox (middle) and custom color selection (right):

## Other options controlling heatmap appearance

The heatmap output shares many display options from other tabs. Here we provide a list of these inputs, please refer to “Viewing and manipulating plots” for further reference.

- **Titles and axis panel**
  - `X-axis label` - Label shown below horizontal axis, drawn separately for each sub-heatmap; default empty
  - `Y-axis label` - Label shown next to vertical axis, drawn separately for each sub-heatmap; default empty
  - `Labels font size` - Font size for axis labels and main labels of sub-heatmaps; default 16 points
  - `Axis font size` - Controls axis ticks font size; default 14 points
  - `Set X-axis limits` - Set hard plotting limits for X-axis; default values are whole range chosen during plot set calculation
- **Guide lines and data scaling panel**
  - `Transform signal` - if set to *`Log2 transform`* performs log2 transformation of the signal prior to plotting; default setting is *`Do not transform`*
  - `Show vertical guide line` - show the vertical line at point 0 - beginning of the feature or midpoint and end of the pseudo-length scaled features (only for anchored plots); turn on by default
- **Keys, labels and colors panel**
  - `Colors` - for hetmaps this input allows to control the color mapping of specific sub-heatmaps. The map starts with white (for low color key limit) and finishes with selected color (for high color key limit).
  - `Label` - allows to set up custom sub-heatmap top labels
  - `Priority/Order` - Use this to place heatmaps in your desired order. The feature-track pairs with the highest priority will be plotted as left-most sub-heatmaps.
  - `Legend font size` - control the font size of common color key, inactive if heatmaps have individual color keys; 12 default

---

# Getting PDFs and cluster info

Plots can be downladed as PDFs by clicking `Line plot` or `Heatmap` buttons in the “Download:” section of the **tool panel** (above the plot preview).


The small buttons next to `Line plot` and `Heatmap` produce additional output files:

- the `i` button next to `Line plot` downloads the PDF containing average plot keys
- `cluster diagram` button next to `Heatmap` downloads a cluster report giving cluster assignments and sorting order for each feature as a comma separated value (CSV) spreadsheet.

The cluster report contains following columns:

- `chromosome` - the name of chromosome, contig or scaffold
- `start` - start of the feature (1 based chromosomal coordinate)
- `end` - end of the feature (1 based chromosomal coordinate)
- `width` - width of the feature in base pairs
- `strand` - strand of the feature
- `metadata_...` - annotation columns present in the original GFF/BED e.g. gene name, score, group
- `originalOrder` - number of feature (row) in GFF/BED, can be used to restore original order after sorting on cluster ID
- `ClusterID` - the numeric ID of the cluster. The topmost cluster on the heatmap is annotated with 1, and the bottom cluster with k, where k equals to number of clusters selected, exported only if clustering is enabled
- `SortingOrder` - the order imposed on heatmap by sorting by mean row(s) values, exported only if sorting is enabled
- `FinalOrder` - the final order of heatmap’s rows, this can be influenced by sorting and clustering; 1 indicates topmost row

Sample report:

```
chromosome  start   end     width   strand  metadata_group  originalOrder   ClusterID   SortingOrder    FinalOrder
          chrI        9065087 9070286 5200    +       g1              1               1           3               3
          chrI        5171285 5175522 4238    -       g1              2               3           50              43
          chrI        9616508 9618109 1602    -       g1              3               3           13              43
          chrI        3608395 3611844 3450    +       g1              4               3           11              12
```

Table view:

| chromosome | start | end | width | strand | metadata\_group | originalOrder | ClusterID | SortingOrder | FinalOrder |
| --- | --- | --- | --- | --- | --- | --- | --- | --- | --- |
| chrI | 9065087 | 9070286 | 5200 | + | g1 | 1 | 1 | 3 | 3 |
| chrI | 5171285 | 5175522 | 4238 | - | g1 | 2 | 3 | 50 | 43 |
| chrI | 9616508 | 9618109 | 1602 | - | g1 | 3 | 3 | 13 | 43 |
| chrI | 3608395 | 3611844 | 3450 | + | g1 | 4 | 3 | 11 | 12 |

## PDF output size

The last tab (`Batch operation and setup`) on the **tool panel** includes batch operations and various other settings including PDF output size. By default the output PDF will be A4 landscape. This can be changed using the drop-down list to following settings:

- `user defined` - this option reveals two numeric inputs that allows to set output PDF width and height. The values must be given in inches.
- `Legal rotated` - US Legal landscape: 14" by 8.5"
- `A4` - A4 portrait: - 8.27" × 11.69"
- `Letter` - US Letter portrait: 8.5" × 11"
- `Legal` - US Legal portrait: 8.5" × 14"
- `Executive` - a.k.a Monarch paper: 7.25 × 10.5"


---

# Batch operations

Controls to plot multiple plots at once are located on the `Batch operation and setup` tab, just below PDF paper options. It is possible to output the plots to multipage PDF, plot an array of plots on a single page (for average plots) or mix these options together.


The first drop-down controls the type of the plot - either average or heatmap. The second drop down determines the strategy to traverse the **plot grid**. The options include:

- `single` - every single feature-track pair will be plotted on separate plot
- `rows` - the **plot grid** will be traversed by rows, which means one plot that contains all tracks per feature will be prepared
- `columns` - the **plot grid** will be traversed by columns, which means one plot that contains all features per tracks will be prepared

The `multi plot grid` option controls how many plots will be placed on each page of the PDF output, e.g. 1x1 means one plot per one page, while 3x4 means 3 columns and 4 rows of plots. If number of plots exceeds the number of slots on page the new page will be added to the PDF.

`Filter names` will apply a filter to plot titles, which are based on on uploaded file names. For example, if you uploaded 100 files starting with a prefix of “my\_experiment\_”, you can remove this fragment from each plot title and/or heatmap caption by putting this string in `Filter names`.

Finally, pressing `Get PDF` produces the final output file. Please see example below:


---

# Saving and loading plotsets

If desired, SeqPlots will save plot sets as binary R files, allowing you to quickly load the pre-calculated set for replotting. Saved plot sets can also be shared with other SeqPlots users.

## Load or save plotset

Controls available on the “Load or save plotset” panel:

- **`Load saved plot set`** - drop-down list to select a plotset. Once the Rdata binary file is selected the **plot grid** will be displayed. Selecting the file reveals two additional buttons:
  - **`Remove dataset`** - this button deletes the selected saved plot set from user data.
  - **`Download plotset`** - this button saves a copy of the plotset in selected location.
- **`Save current plot set`** - Enter desired name and press the `Save` button (appears after input of name). It is also possible to save a copy of loaded plot sets. The plot set binary files can be renamed simply by loading them, saving a copy and deleting original source file.

All saved dataset can be found in `data location`/publicFiles. Any SeqPlots Rdata binaries put in the folder will become available for loading in **`Load saved plot set`** control.

## Plot set files structure

The plot sets files can be also directly loaded in R. This allows further processing and customization of the plots. Data structure is a nested list, which elements be accessed by `[[` R operator. The nesting goes as follow:

- **`feature`** - R list
  - **`track`** - R list
    - `means` - numeric vector giving mean signal value for each (binned) genomic position
    - `stderror` - numeric vector giving standard error for each (binned) genomic position
    - `conint` - numeric vector giving 95% confidence interval for each (binned) genomic position
    - `all_ind` - numeric vector giving the genomic position in base pairs
    - `e` - character string giveing numeric vector giving the indicates of anchored distance, NULL for point features plots
    - `desc` - auto generated title of the plot
    - `heatmap` - numeric matrix, (binned) signal values for each genomic position (columns) and each feature (rows)

The example structure:

```
List of 2
           $ HTZ1_Differential_genes_TOP100_v2.gff:List of 2
            ..$ HTZ1_JA00001_IL1andIL2_F_N2_L3_NORM_linear_1bp_IL010andIL009_averaged.bw    :List of 7
            .. ..$ means   : num [1:501] 2.52 2.52 2.52 2.53 2.54 ...
            .. ..$ stderror: num [1:501] 0.114 0.112 0.111 0.11 0.109 ...
            .. ..$ conint  : num [1:501] 0.226 0.223 0.221 0.218 0.217 ...
            .. ..$ all_ind : num [1:501] -1000 -995 -990 -985 -980 -975 -970 -965 -960 -955 ...
            .. ..$ e       : NULL
            .. ..$ desc    : chr "HTZ1_JA00001_IL1andIL2...\n@HTZ1_Differential_genes_TOP100_v2"
            .. ..$ heatmap : num [1:100, 1:501] 2.36 5.25 2.2 3.48 4.32 ...
            ..$ HTZ1_JA00001_IL3andIIL5_F_lin35_L3_NORM_linear_1bp_IL008andIL011_averaged.bw:List of 7
            .. ..$ means   : num [1:501] 2.36 2.35 2.35 2.36 2.38 ...
            .. ..$ stderror: num [1:501] 0.126 0.125 0.125 0.126 0.125 ...
            .. ..$ conint  : num [1:501] 0.249 0.249 0.247 0.251 0.249 ...
            .. ..$ all_ind : num [1:501] -1000 -995 -990 -985 -980 -975 -970 -965 -960 -955 ...
            .. ..$ e       : NULL
            .. ..$ desc    : chr "HTZ1_JA00001_IL3andIIL5...\n@HTZ1_Differential_genes_TOP100_v2"
            .. ..$ heatmap : num [1:100, 1:501] 2.61 3.17 1.42 2.46 4.26 ...
           $ HTZ1_Differential_genes_BOTTOM100.gff:List of 2
            ..$ HTZ1_JA00001_IL1andIL2_F_N2_L3_NORM_linear_1bp_IL010andIL009_averaged.bw    :List of 7
            .. ..$ means   : num [1:501] 1.57 1.57 1.58 1.6 1.62 ...
            .. ..$ stderror: num [1:501] 0.0996 0.0985 0.1003 0.1022 0.1018 ...
            .. ..$ conint  : num [1:501] 0.198 0.195 0.199 0.203 0.202 ...
            .. ..$ all_ind : num [1:501] -1000 -995 -990 -985 -980 -975 -970 -965 -960 -955 ...
            .. ..$ e       : NULL
            .. ..$ desc    : chr "HTZ1_JA00001_IL1andIL2...n@HTZ1_Differential_genes_BOTTOM100"
            .. ..$ heatmap : num [1:100, 1:501] 1.64 1.37 1.61 1.77 1.86 ...
            ..$ HTZ1_JA00001_IL3andIIL5_F_lin35_L3_NORM_linear_1bp_IL008andIL011_averaged.bw:List of 7
            .. ..$ means   : num [1:501] 1.94 1.94 1.95 1.96 1.97 ...
            .. ..$ stderror: num [1:501] 0.123 0.123 0.124 0.126 0.128 ...
            .. ..$ conint  : num [1:501] 0.244 0.245 0.246 0.251 0.253 ...
            .. ..$ all_ind : num [1:501] -1000 -995 -990 -985 -980 -975 -970 -965 -960 -955 ...
            .. ..$ e       : NULL
            .. ..$ desc    : chr "HTZ1_JA00001_IL3andIIL5...\n@HTZ1_Differential_genes_BOTTOM100"
            .. ..$ heatmap : num [1:100, 1:501] 1.61 1.37 1.29 3.04 3.77 ...
```

---

# Advanced options

Some additional SeqPlots options are located at very bottom of the `Batch operation and setup` tab:


- `Keep 1:1 aspect ratio in batch mode` - This option guarantee that the ratio between X- and Y-axis height will be 1, hence the produced plots will be square in batch mode. This prevents stretching the plots while fitting single rows or columns to one page. Turned on by default.
- `Always keep 1:1 aspect ratio` - This checkbox extends the 1:1 aspect ratio option to single plots. Turned off by default.
- `Reactive plotting` - When selected, all plotting operations are executed upon selection and will be visible in preview. `Reactive plotting` might be useful for exploratory data analysis using plots, but it is not recommended for heatmap plots because speed is decreased. Select/delelect from keyboard by pressing [ctrl/cmd+R]. Turned off by default.
- `Use multithreading for calculations` - This option is available only on desktop instances of SeqPlots under Mac OS X and Linux. While turned off R will not fork the child processes for plotting and plot set calculations. It is useful for debugging, since in single process mode all warning/errors will be directly printed to R console. Also might increase the performance for plotting small average plots. Turned off by default.
- `Use ggplot2 graphics package for heatmaps` - uses GGplot2 (http://ggplot2.org/) graphics system to draw the heatmaps. This feature is experimental.

---

# Genomes managment

The `Manage reference genomes` tab allows to add or remove reference genomes installed with SeqPlots. New genomes are added to user data folder, so they can be easily moved along with data and would not be removed when updating R or SeqPlots. User can choose to install standard genomes package available with Bioconductor or install forged genomic package from local file.


---

# Error messages

```
Problem with line N: "line_text" [internal_error]
```

> The import of feature file (GFF or BED) was not successful due to mis-formatted file.

---

```
Chromosome names provided in the file does not match ones defined in reference genome. 
          INPUT: [chr3R, chr2L, chr2R, chr3L] 
          GENOME: [chrI, chrII, chrIII, chrIV, chrV, ...]
```

> There are unexpected chromosome names in input file. Following genomes: *Arabidopsis thaliana, Caenorhabditis elegans, Cyanidioschyzon\_merolae, Drosophila melanogaster, Homo sapiens, Oryza sativa, Populus trichocarpa, Saccharomyces cerevisiae and Zea mays* support chromosome names remapping between different naming conventions, including: AGPv2, ASM9120v1, Ensembl, JGI2\_0, MSU6, NCBI, TAIR10 and UCSC. If you see above error in one of these genomes there are still unexpected names after the correction. The problematic chromosome names are given in the error message. Remove GFF/BED lines corresponding to them or upgrade the genome to one containing proper naming. Alternatively set genome to NA.

---

```
File already exists, change the name or remove old one.
```

> File named like this already exists in the database, it is impossible to have two files sharing same filename.

---

```
ERROR: solving row 300: negative widths are not allowed
```

> The the row 300 have end coordinate smaller than beginning, hence the width in negative. To fix it the start and stop indicates should be swapped. This error often happens when negative strand (-) ranges are misformatted.

---


---

# Versions history

## CHANGES IN VERSION 1.4.1

PACKAGE:

- RJSONIO replaced with jsonlite package for encoding/decoding JSON
- Sub-heatmaps accepts multilevel color palettes in plotHeatmap function
- Better dendrogram positioning for heatmaps with hclust and single sub-heatmap

GUI:

- “Load saved plot set” select input replaced with searchable selectize input
- Tracks and feature names are shown without extensions
- Plot Preview GUI elements are shown directly after calculation finishes or save dataset si loaded, even if no pair is selected.
- Download buttons moved to bottom of side panel
- Color palettes from RColorBrewer package can be selected in GUI
- Web interface tab for managing the reference genomes

BUGFIX:

- Plot Preview shows up when needed with shiny 0.12.0 (issue #6)
- Heatmap top labels and color key font sizing works properly
- Clustering report download works when source feature file were deleted

## CHANGES IN VERSION 1.3.0

- New package version for Bioconductor 3.1 release

## CHANGES IN VERSION 1.2.0

PACKAGE:

- plot a dendrogram as first panel on heatmap plot clustered with hierarchical clustering
- heatmap can be plotted as vector graphics (default for R scripting) or raster graphics (default for web GUI)
- heatmaps can be sorted in increasing or decreasing order
- y-axis is annotated with cluster IDs and number of data rows (heatmap)
- x-axis is annotated with base pairs, e.g. 10bp, 1kb, 1.2Mb, etc.
- heatmaps can be plotted using GGplot2 package instead of R base graphics system
- speed-up in motif plots thanks to faster, vectored implementation of motif density data acquisition
- code simplification - seqplots in Shiny GUI mode use all functions from package core

GUI:

- feature-track pairs selection grid have UI elements to multi-select and batch change labels/colors/etc.
- option to make a clustering repeatable (works by reusing a .Random.seed)
- option to plot selected cluster only as heatmap
- SeqPlots GUI updated to work with Bootstrap v3.3.1

BUGFIX:

- lines separating the clusters are drawn in correct places on grDevices::quartz devices
- proper positioning of 3’ ends of anchored region in motif plot
- fixed error with anchored plots and genomic feature width equals 1bp
- y-axis does respect cex.axis parameter
- GUI fixed to work with shiny 0.11.0 and above

## CHANGES IN VERSION 1.0.0

PACKAGE:

- plotHeatmap function returns cluster report as GRanges structure
- redundant parameters removed from plotting functions
- plotHeatmap function have “embed” parameter for plotHeatmap - allows to plot 1st heatmap without using grid system, intended to use with complex plots

BUGFIX:

- motif plot orientation properly dependents on strand
- GUI - reordering the heatmap respects previously set include/exclude parameters

## CHANGES IN VERSION 0.99.1

PACKAGE:

- heatmap plotting function returns cluster report ad data.table
- getPlotSetArray function have “verbose” parameter that controls messages and warnings output
- references added to documentation

BUGFIX:

- plotHeatmap and plotAverage generic methods for SeqPlots-classes respect the parameters
- package passes tests and check on 32bit Windows (plotting only, because no rtrackalyer::BigWigFile support for Win32)

## CHANGES IN VERSION 0.99

GENERAL:

- Anchored plots and heatmaps uses [downstream]–0–0–[upstream] X-axis coordinate system instead [downstream]–0–[anchored]-[upstream+anchored]

PACKAGE:

- package really on reference class system including MotifSetup, PlotSetArray, PlotSetList and PlotSetPair
- generic subset and data manipulation methods for SeqPlots-classes including ‘[’, “[[” and “unlist”, which allows to switch between classes
- automatic tests for class system, calculations and plotting functions
- documentation for all functions and classes
- PDF vignette engine replaced by HTML one
- QuickStart vignette added

GUI:

- automated GUI tests using Rselenium package

BUGFIX:

- issue #1: some server instances loads empty .Rdata file on startup

## CHANGES IN VERSION 0.9.3

- The web GUI and R package projects merged into singe project distributed as Bioconductor compatible R package
- The command line interface have the same capabilities as GUI version
- Web GUI vignette added

## CHANGES IN VERSION 0.9.2

GENERAL:

- use Cairo package for plotting, X11 installation no longer required
- colors in plot grid are initiated automatically (same color palette as for auto-generated average plots), white color is allowed
- more informative error messages during file upload
- documentation is integrated with SeqPlots GUI help
- Web GUI debug console added
- Exit button, that closes web interface and background R process

BUGFIX:

- fixed errors reporting in singe core/Windows mode
- the custom color gradient controls for hetmap (three color pickers) work correctly now
- heatmap main title no longer overlaps with sub-plot labels

## CHANGES IN VERSION 0.9.1

GENERAL:

- inputs and features sorted alphabetically
- DataTables v1.10.0 with pagination, selection number indicator and infinite row selections
- buttons for heatmap and lineplots, PDF default sizes,
- changes in GUI layout
- default PDF output paper size set to A4 horizontal,
- Font sizes are in points
- preview is compatible with A4 PDF output (at 100 DPI)
- color key for heatmap are always generated using image.plot function that provides better labeling
- batch plots do not override individual labels if set
- option to keep 1:1 aspect ratio (default for batch plots)
- miscellaneous options renamed for clarity

## CHANGES IN VERSION 0.9.0

FEATURES:

- Multi-plot grid option in batch mode - many line plots on single page

GENERAL:

- R 3.1 and BioC 2.14 compatibility
- faster BigWig signal retrieval, no need for modified rtracklayer C code in the package
- warning message if JS File API is not supported (old browsers)
- improved the performance of heatmap plotting by using list of matrices instead concatenated matrix

BUGFIX:

- application start properly without any BSGenome genomic packages installed
- cluster report - the final order agrees with cluster indicates

## CHANGES IN VERSION 0.8.2

FEATURES:

- Hierarchical and super self-organizing network clustering added for heatmaps
- Anchored motif plots
- The row order of the heatmap is exported along with cluster report

GENERAL:

- JS color picker added for browsers, that do not support select input type=“color” i.e. Firefox (checked with modernizr.js library)
- Single process mode and Microsoft Windows compatibility (running without fork parallelization)
- Shiny 0.9.1 compatibility
- Saved datasets can be downloaded for local usage
- Clicking row or column name in plot grid toggles the checkboxes
- Minor GUI changes

## CHANGES IN VERSION 0.8.1

GENERAL:

- GUI redesign: plot matrix incorporates sub-plot/heatmap specific controls, all heatmap options gathered in single tab
- warning before closing/refreshing a webpage with active session
- cookie based default options: user, genome and deactivate page exit warning
- heat-map clusters provided as cluster report - a CSV file containing original features, annotations and cluster information, see more: https://bitbucket.org/przemol/seqplots/wiki/Heatmaps#markdown-header-cluster-report
- Wiggle files processing: correct for multiple header definitions and roman/arabic chromosome names correction
- Optimised keyboard shortcuts: plot - RETUTRN or ctrl/cmd+SPACE, switch heatmap - ctrl/cmd+H, switch reactive plotting - ctrl/cmd+R
- minor speed improvement

BUGFIX:

- Motif density plots and heatmaps: flip rows on (-) strand

## CHANGES IN VERSION 0.8.0

GENERAL:

- GUI redesign, option partitioned to more tabs
- preview plot is zoomed on click rather than on mouse hover
- possibility to remove multiple files
- comments visible as popup in file managmed window
- all chromosome naming conventions (most notably chrX/X and variants of chrM/M/MtDNA/MT etc.) are accepted (http://www.bioconductor.org/packages/release/data/annotation/html/seqnames.db.html)
- incoming featurefiles (GFF and BED) are not processed, just chacked for errors
- explicit error handling for incoming flies, the line with problem or unexpected chromosome(s) are indentified to the user
- motif density tracks can be binned (defoult at 10bp)
- tracks amd motif densities cna be mixed together in plots

SERVER:

- server\_config.R added - a configuration file that allows to set up server varaiables, e.g. the user data location

MAC OS X APP:

- interface to insrall new genomes from Bioconductor and local resources (R BSgemome format: http://www.bioconductor.org/packages/release/bioc/html/BSgenome.html)
- option to set up data location

## CHANGES IN VERSION 0.7.0

- SeqPlots for Mac OS X relesed - an user frienddly wrapper app containig R, packages and SeqPlots coede
- heatmap plotting added
- motif denstty plotting added for lineplot and heatmap
- minior interface redesign
- reactive interface can be turn off for plottting, user plots on demend
- adding files from jQuery File Upload (http://blueimp.github.io/jQuery-File-Upload/) is handled directly by R eliminating additional node.js server application and making proper file handling for desktop version
- computationally expensive operations (calculating plot matrix and plotting) are handeled by new R process (parallel R library) - many proces can run simmutainously in same Shiny instance, user can get fedback from the calcualtion can be cancelled

## CHANGES IN VERSION 0.6.0

- Shiny (https://github.com/rstudio/shiny) used as R web fraimwork, support for Rserver/EXT JS version dropped
- support for 145 genomes from UCSC database (via user provideing valid genome symbol)
- new reactive user interface
- new plot type: midpoint features - it calculates the middle of given features and centres the summary on it
- the option to ignore the strand (plot always in the same direction)
- the option to remove the zeros (0 value of score in Wiggle track) from mean and error estimate calculations
- the support for BED feature files (in addition to GFF, Wiggle (all variants), BigWiggle)
- automatic chromosome name correction for C. elegans genomes (I => chrI, MtDNA => chrM, etc.)
- accepts wiggle with overlapping ranges (e.g. microarray experiments processed using MA2C)
- basic user management for uploaded files
- option to download the features and track files directly from application

## CHANGES IN VERSION 0.5.0

- Initial test and alpha releases

---

# Explanations

---

- “**feature**” - a genomic interval defined by **chromosome** name, **start** and **end** positions and the **directionality** (strand). The end must always be a bigger number than start, so the width of the range is not negative. Start and end means here the numeric start of the interval and should not be confused with TSS and TTS.

  For example, in BED format this information is stored in following text tab delimited format: `chr7 127471196 127472363 . . +`

  ---
- “**directionality**” - the strand of genomic feature, determining if the plotting range should be anchored around the star or and, and the direction in which signal is being processed to create the average track or heatmap. Unknown directionality is marked by `*` and treated as `+` for calculations.

  ---
- “**track**” - the file assigning the continuous signal (score) to genomic locations across the chromosomes. The signal usually comes from sequencing experiments, like ChIP-seq, RNA-seq, DNase-seq, MNase-seq, or from computational tools, for example nucleosome occupancy prediction, CpG density.

  For example, in BedGraph format this information is stored in following text tab delimited format: `chr19 49302300 49302600 -0.75`

  ---
- “**reference genome package**” - the R BSgemome package containing the full reference sequence for given species. It is also used to provide universal chromosome names and chromosome lengths taht are used as plotting boundaries.

  ---
- “**reads coverage**” - The basic way to calculate the signal from sequencing based assays. The numeric representation shows how much reads was aligned to given genomic location. This can be a proxy to protein-DNA binding (ChIP-seq) or the expression (RNA-seq). Can be calculated using BedTools: http://bedtools.readthedocs.org/en/latest/content/tools/genomecov.html Also known as `pileups`.

  ---

---

# References

> This section lists R, JavaScript and CSS libraries used by SeqPlots, important conceptual contributions to the software, and publication, where figures generated by SeqPlots are featured.

**R project and Bioconductor**

- R Core Team (2014). R: A language and environment for statistical computing. R Foundation for Statistical Computing, Vienna, Austria. URL http://www.R-project.org/.
- Bioconductor: Open software development for computational biology and bioinformatics R. Gentleman, V. J. Carey, D. M. Bates, B.Bolstad, M.Dettling, S. Dudoit, B. Ellis, L. Gautier, Y. Ge, and others 2004, Genome Biology, Vol. 5, R80. URL http://www.bioconductor.org/.
- RStudio and Inc. (2014). shiny: Web Application Framework for R. R package version 0.10.1. http://shiny.rstudio.com/
- **Other CRAN packages:** digest, DBI,RSQLite, RJSONIO, plotrix, fields, grid, kohonen, ggplot2, Cairo and parallel
- **Bioconductor packages:** IRanges, BSgenome, Rsamtools, rtracklayer, GenomicRanges and Biostrings

**JavaScript and CSS**

- jQuery framework - http://jquery.com
- Bootstrap - http://getbootstrap.com
- DataTables, Table plug-in for jQuery - http://www.datatables.net
- jQuery File Upload Plugin - https://github.com/blueimp/jQuery-File-Upload
- jQuery throttle - http://benalman.com/projects/jquery-throttle-debounce-plugin/
- jQuery Cookie Plugin - https://github.com/carhartl/jquery-cookie
- Modernizer JS library - http://modernizr.com
- JavaScript Templates - https://github.com/blueimp/JavaScript-Templates
- JavaScript Color Picker - http://jscolor.com
- md5-js - https://github.com/wbond/md5-js
- Font Awesome - http://fortawesome.github.io/Font-Awesome
- Google Fonts - https://www.google.com/fonts
- jQuery user interface - http://jqueryui.com (documentation)
- jquery.tocify.js: jQuery Table of Contents - https://github.com/gfranko/jquery.tocify.js (documentation)
- Strapdown https://github.com/arturadib/strapdown (documentation)
- Bootswatch themes - http://bootswatch.com (documentation)
- google-code-prettify - https://code.google.com/p/google-code-prettify (documentation)
- marked - https://github.com/chjj/marked (documentation)

**Important conceptual contribution to the project**

- Liu T, Ortiz J, Taing L, Meyer C, Lee B, Zhang Y, Shin H, Wong S, Ma J, Lei Y, et al. 2011. Cistrome: an integrative platform for transcriptional regulation studies. Genome Biology 12: R83.
- Thomas Williams, Colin Kelley and others (2010). Gnuplot 4.4: an interactive plotting program. URL http://www.R-project.org/.
- Kent, W.J., Sugnet, C.W., Furey, T.S., Roskin, K.M., Pringle, T.H., Zahler, A.M. and Haussler, a. D. (2002). The Human Genome Browser at UCSC. Genome Research. 12:996–1006.
- Kent WJ, Zweig AS, Barber G, Hinrichs AS, Karolchik D. (2010). BigWig and BigBed: enabling browsing of large distributed datasets. Bioinformatics. 1;26(17):2204-7
- Nicol, J.W., Helt, G.A., Blanchard, S.G., Raja, A. and Loraine, A.E. (2009). The Integrated Genome Browser: free software for distribution and exploration of genome-scale datasets. Bioinformatics (Oxford, England). 25:2730–1.
- Thorvaldsdóttir, H., Robinson, J.T. and Mesirov, J.P. (2012). Integrative Genomics Viewer (IGV): high-performance genomics data visualization and exploration. Briefings in bioinformatics. bbs017

**Server deployment**

- Shiny Server - https://github.com/rstudio/shiny-server
- ShinyApps - https://github.com/rstudio/shinyapps

**Publications containing figures made by SeqPlots**

- Chen RA, Stempor P, Down TA, Zeiser E, Feuer SK, Ahringer J. Extreme HOT regions are CpG-dense promoters in C. elegans and humans. Genome Res 24(7):1138-1146 Jul 2014
- Latorre I, Chesney MA, Garrigues JM, Stempor P et al. The DREAM complex promotes gene body H2A.Z for target repression. Genes Dev 2015 Mar 1;29(5):495-500.

---

- Welcome to **SeqPlots**
  - Summary
  - Availability
  - Key features
  - Video presentation
  - Issues and bugs
- Installation - Mac OS X app
  - How to install
  - How to start
  - Package content
- Installation - R/Bioconductor
  - How to install
  - How to start
- Installation - Server deployment
  - Installation
  - Usage
  - Alternative installation
- Installation - Docker image
  - How to install Docker
  - How to use on Mac and Windows
  - How to use Linux
  - Troubleshooting
  - Package content
- Quick start demo
- Adding and managing files
  - Supported file formats
  - Adding files
  - Downloading and removing files
- Running the plot-set jobs
  - Selecting files
  - Setting up plot options
  - Plotting sequence motif density
  - Starting the plot set calculation
- Plotting
  - Previewing plot
  - Titles and axis panel
  - Guide lines and data scaling
  - Keys, labels and colors panel
- Heatmaps
  - Heatmap setup tab
  - Other options controlling heatmap appearance
- Getting PDFs and cluster info
  - PDF output size
- Batch operations
- Saving and loading plotsets
  - Load or save plotset
  - Plot set files structure
- Advanced options
- Genomes managment
- Error messages
- Versions history
  - CHANGES IN VERSION 1.4.1
  - CHANGES IN VERSION 1.3.0
  - CHANGES IN VERSION 1.2.0
  - CHANGES IN VERSION 1.0.0
  - CHANGES IN VERSION 0.99.1
  - CHANGES IN VERSION 0.99
  - CHANGES IN VERSION 0.9.3
  - CHANGES IN VERSION 0.9.2
  - CHANGES IN VERSION 0.9.1
  - CHANGES IN VERSION 0.9.0
  - CHANGES IN VERSION 0.8.2
  - CHANGES IN VERSION 0.8.1
  - CHANGES IN VERSION 0.8.0
  - CHANGES IN VERSION 0.7.0
  - CHANGES IN VERSION 0.6.0
  - CHANGES IN VERSION 0.5.0
- Explanations
- References
