# Supplementary material for: McClintock: An Integrated Pipeline for Detecting Transposable Element Insertions in Whole-Genome Shotgun Sequencing Data
Source: G3 (Bethesda). 2017 Jun 21;7(8):2763–78. doi: 10.1534/g3.117.043893 (PMC5555480; doi:10.1534/g3.117.043893)
Supplement: Supplementary file 3 [file 2763FileS3.zip › mcclintock_analysis_code/seqplots-master/inst/seqplots/www/tutorial.html]

# Welcome to SeqPlots tutorial

---

###


Previous


Next

You can stop the tutorial at any time by pressing [ESC] key.

---

Skip
Start tutorial
