# Supplementary material for: McClintock: An Integrated Pipeline for Detecting Transposable Element Insertions in Whole-Genome Shotgun Sequencing Data
Source: G3 (Bethesda). 2017 Jun 21;7(8):2763–78. doi: 10.1534/g3.117.043893 (PMC5555480; doi:10.1534/g3.117.043893)
Supplement: Supplementary file 3 [file 2763FileS3.zip › mcclintock_analysis_code/seqplots-master/inst/seqplots/www/upload/upload.html]

x

### Upload files

Info
Use "Add files" button or drag and drop files here.
Specify genome version and user initials. Comments are optional.
You can click the person/genome/comment icon to assign field value to multiple files.

Add files...


Start upload


Cancel upload


Set defaults...

*User*

*Genome*

Warn on page exit.

Submit
 Cancel

---
