# Supplementary figures and images for: McClintock: An Integrated Pipeline for Detecting Transposable Element Insertions in Whole-Genome Shotgun Sequencing Data
Source: G3 (Bethesda). 2017 Jun 21;7(8):2763–78. doi: 10.1534/g3.117.043893 (PMC5555480; doi:10.1534/g3.117.043893)

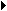

Supplement: Supplementary file 3 [file 2763FileS3.zip › mcclintock_analysis_code/seqplots-master/inst/seqplots/www/color/arrow.gif]

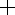

Supplement: Supplementary file 3 [file 2763FileS3.zip › mcclintock_analysis_code/seqplots-master/inst/seqplots/www/color/cross.gif]

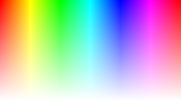

Supplement: Supplementary file 3 [file 2763FileS3.zip › mcclintock_analysis_code/seqplots-master/inst/seqplots/www/color/hs.png]

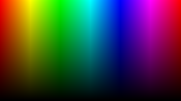

Supplement: Supplementary file 3 [file 2763FileS3.zip › mcclintock_analysis_code/seqplots-master/inst/seqplots/www/color/hv.png]

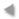

Supplement: Supplementary file 3 [file 2763FileS3.zip › mcclintock_analysis_code/seqplots-master/inst/seqplots/www/css/images/back_disabled.png]

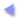

Supplement: Supplementary file 3 [file 2763FileS3.zip › mcclintock_analysis_code/seqplots-master/inst/seqplots/www/css/images/back_enabled.png]

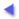

Supplement: Supplementary file 3 [file 2763FileS3.zip › mcclintock_analysis_code/seqplots-master/inst/seqplots/www/css/images/back_enabled_hover.png]

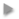

Supplement: Supplementary file 3 [file 2763FileS3.zip › mcclintock_analysis_code/seqplots-master/inst/seqplots/www/css/images/forward_disabled.png]

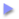

Supplement: Supplementary file 3 [file 2763FileS3.zip › mcclintock_analysis_code/seqplots-master/inst/seqplots/www/css/images/forward_enabled.png]

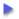

Supplement: Supplementary file 3 [file 2763FileS3.zip › mcclintock_analysis_code/seqplots-master/inst/seqplots/www/css/images/forward_enabled_hover.png]

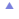

Supplement: Supplementary file 3 [file 2763FileS3.zip › mcclintock_analysis_code/seqplots-master/inst/seqplots/www/css/images/sort_asc.png]

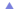

Supplement: Supplementary file 3 [file 2763FileS3.zip › mcclintock_analysis_code/seqplots-master/inst/seqplots/www/css/images/sort_asc_disabled.png]

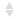

Supplement: Supplementary file 3 [file 2763FileS3.zip › mcclintock_analysis_code/seqplots-master/inst/seqplots/www/css/images/sort_both.png]

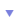

Supplement: Supplementary file 3 [file 2763FileS3.zip › mcclintock_analysis_code/seqplots-master/inst/seqplots/www/css/images/sort_desc.png]

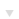

Supplement: Supplementary file 3 [file 2763FileS3.zip › mcclintock_analysis_code/seqplots-master/inst/seqplots/www/css/images/sort_desc_disabled.png]

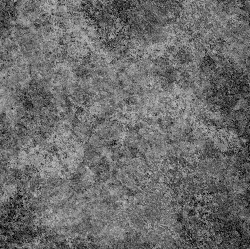

Supplement: Supplementary file 3 [file 2763FileS3.zip › mcclintock_analysis_code/seqplots-master/inst/seqplots/www/css/mask2.png]

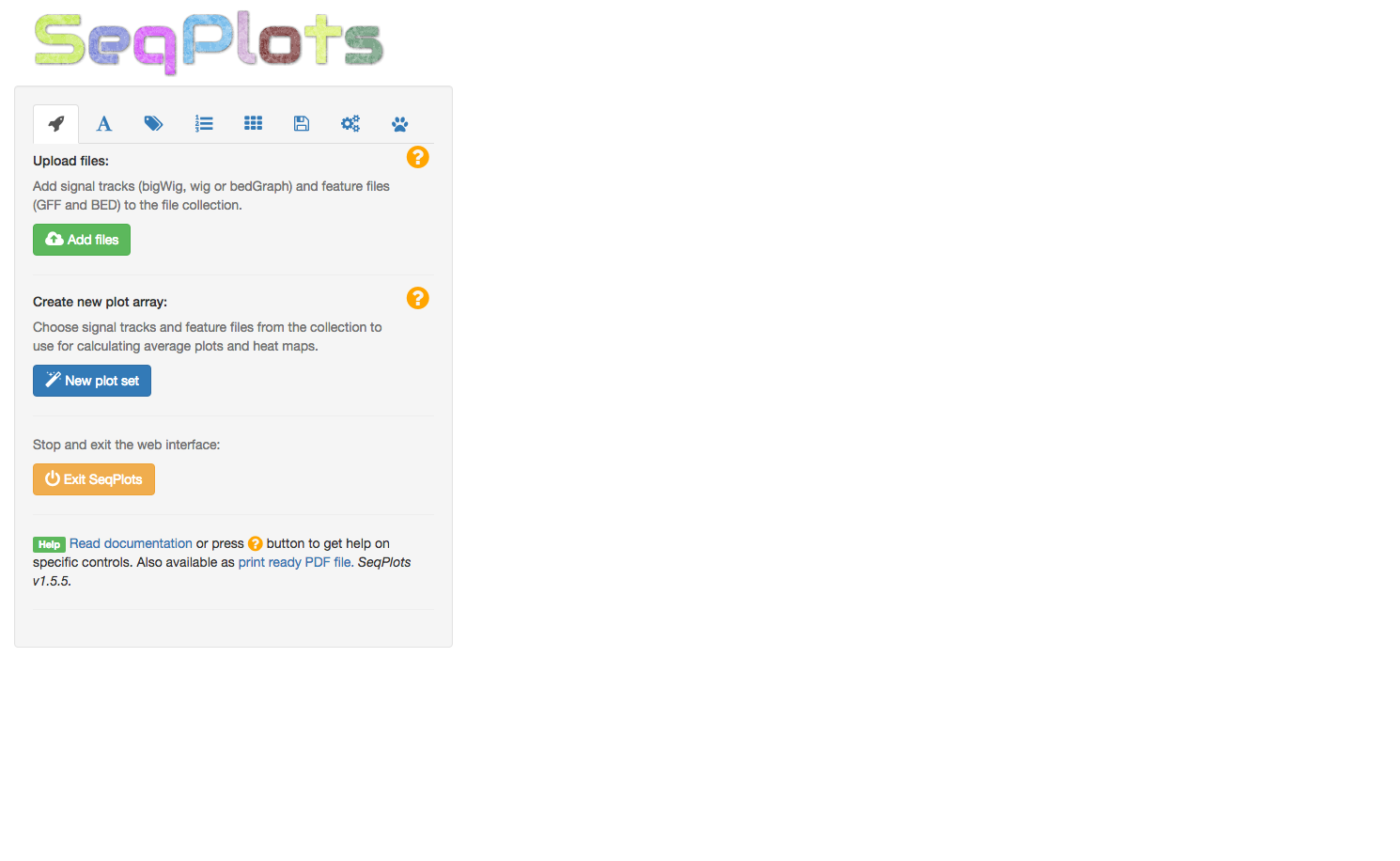

Supplement: Supplementary file 3 [file 2763FileS3.zip › mcclintock_analysis_code/seqplots-master/inst/seqplots/www/help/img/01_QuickStart/01_QuickStart_01.png]

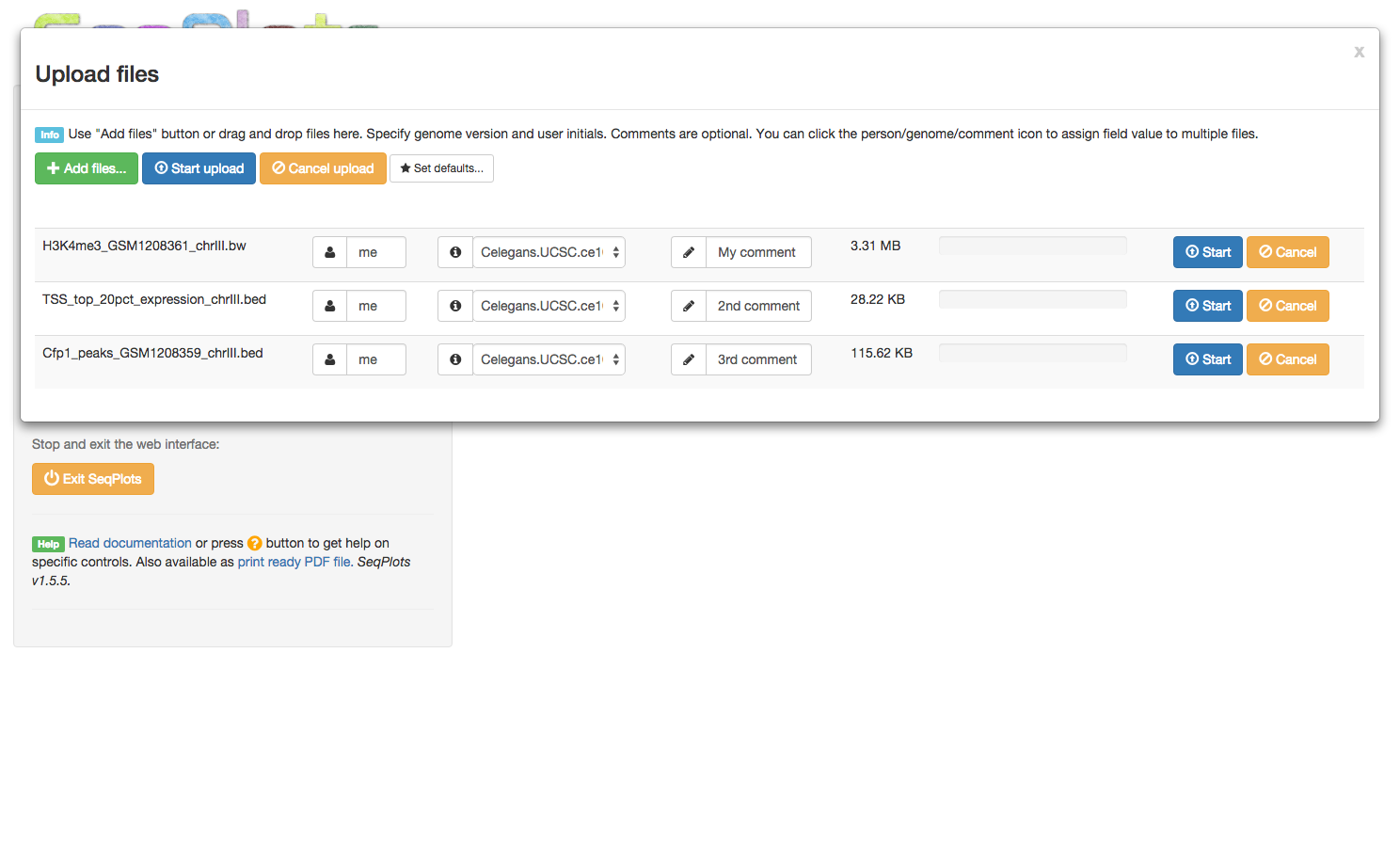

Supplement: Supplementary file 3 [file 2763FileS3.zip › mcclintock_analysis_code/seqplots-master/inst/seqplots/www/help/img/01_QuickStart/01_QuickStart_02.png]

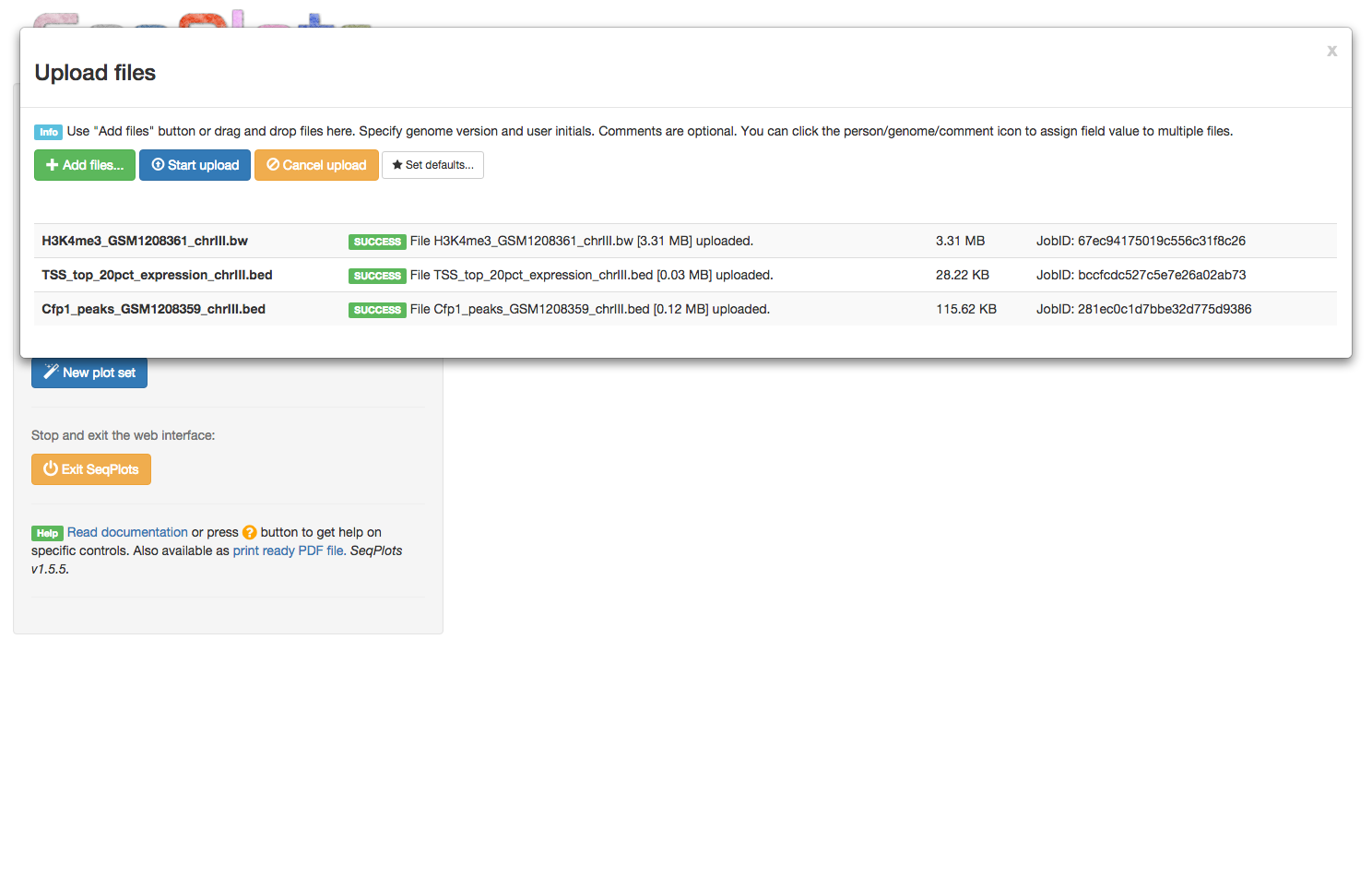

Supplement: Supplementary file 3 [file 2763FileS3.zip › mcclintock_analysis_code/seqplots-master/inst/seqplots/www/help/img/01_QuickStart/01_QuickStart_03.png]

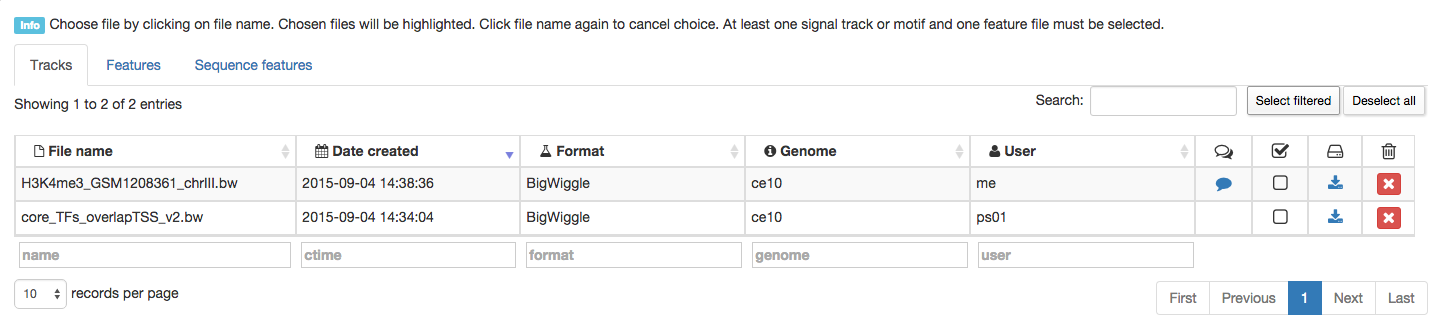

Supplement: Supplementary file 3 [file 2763FileS3.zip › mcclintock_analysis_code/seqplots-master/inst/seqplots/www/help/img/01_QuickStart/01_QuickStart_04.png]

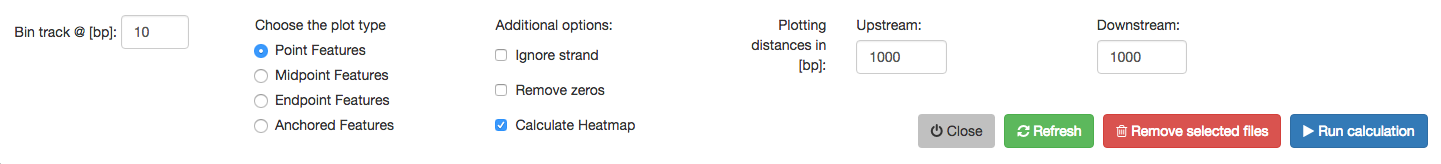

Supplement: Supplementary file 3 [file 2763FileS3.zip › mcclintock_analysis_code/seqplots-master/inst/seqplots/www/help/img/01_QuickStart/01_QuickStart_05.png]

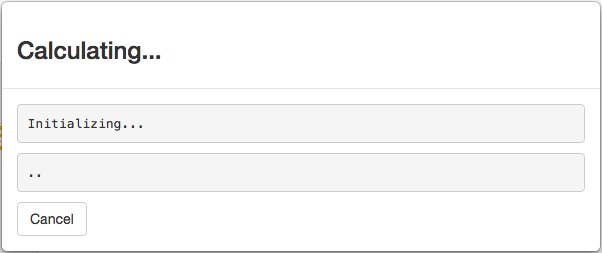

Supplement: Supplementary file 3 [file 2763FileS3.zip › mcclintock_analysis_code/seqplots-master/inst/seqplots/www/help/img/01_QuickStart/01_QuickStart_06.png]

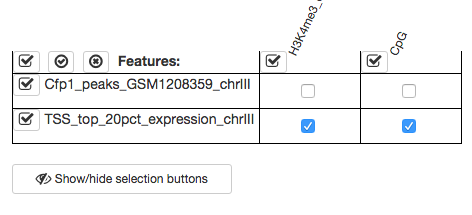

Supplement: Supplementary file 3 [file 2763FileS3.zip › mcclintock_analysis_code/seqplots-master/inst/seqplots/www/help/img/01_QuickStart/01_QuickStart_07.png]

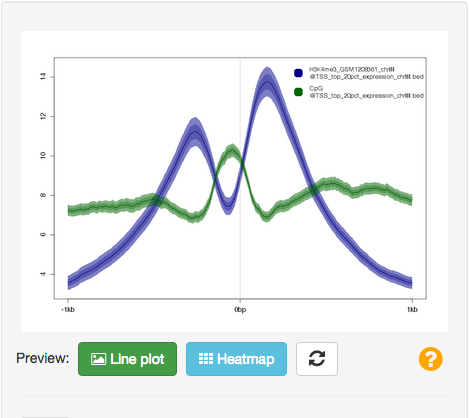

Supplement: Supplementary file 3 [file 2763FileS3.zip › mcclintock_analysis_code/seqplots-master/inst/seqplots/www/help/img/01_QuickStart/01_QuickStart_08.png]

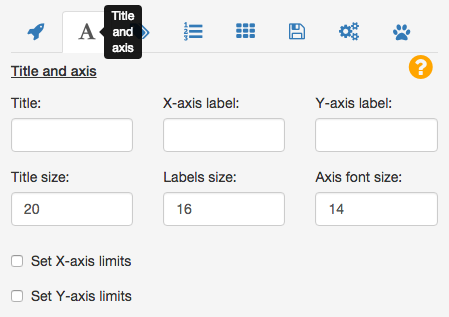

Supplement: Supplementary file 3 [file 2763FileS3.zip › mcclintock_analysis_code/seqplots-master/inst/seqplots/www/help/img/01_QuickStart/01_QuickStart_09.png]

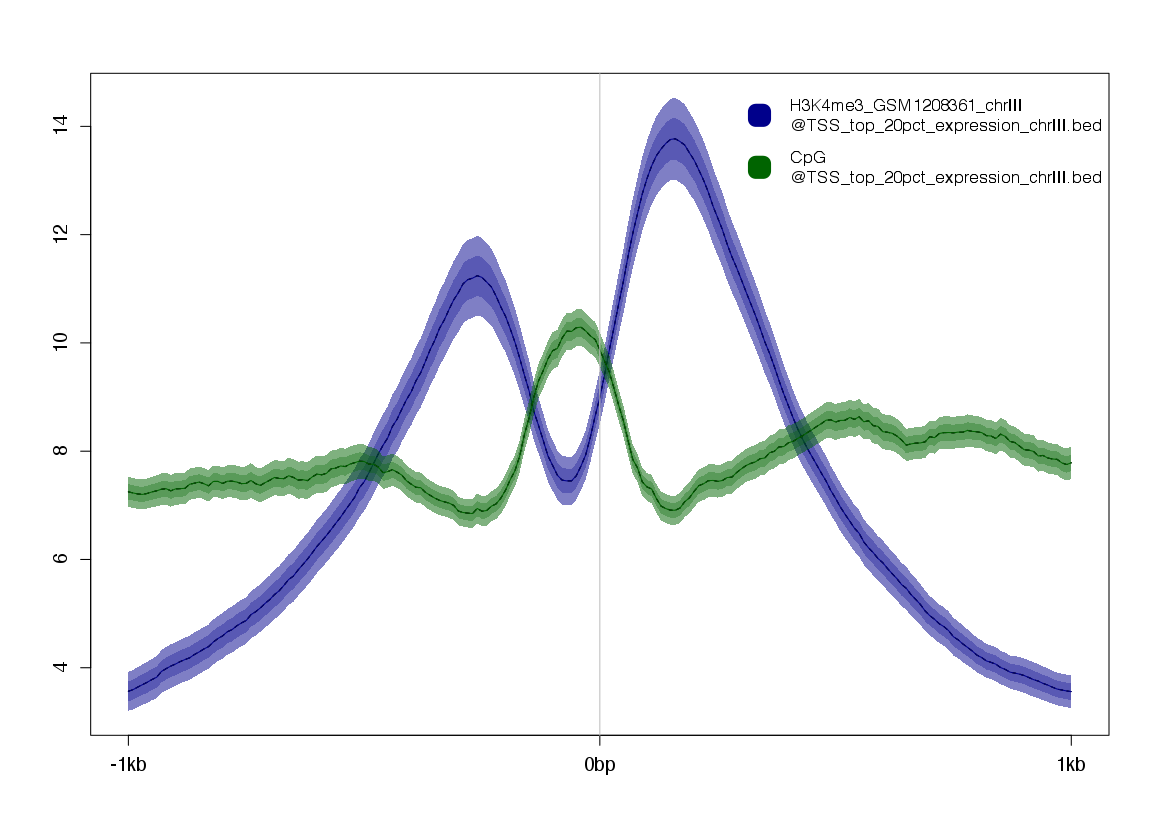

Supplement: Supplementary file 3 [file 2763FileS3.zip › mcclintock_analysis_code/seqplots-master/inst/seqplots/www/help/img/01_QuickStart/01_QuickStart_10.png]

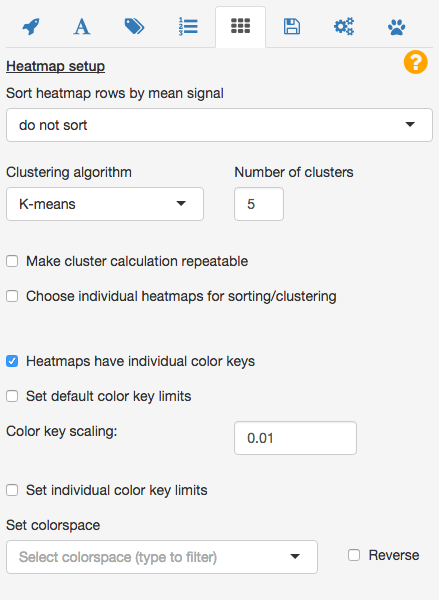

Supplement: Supplementary file 3 [file 2763FileS3.zip › mcclintock_analysis_code/seqplots-master/inst/seqplots/www/help/img/01_QuickStart/01_QuickStart_11.png]

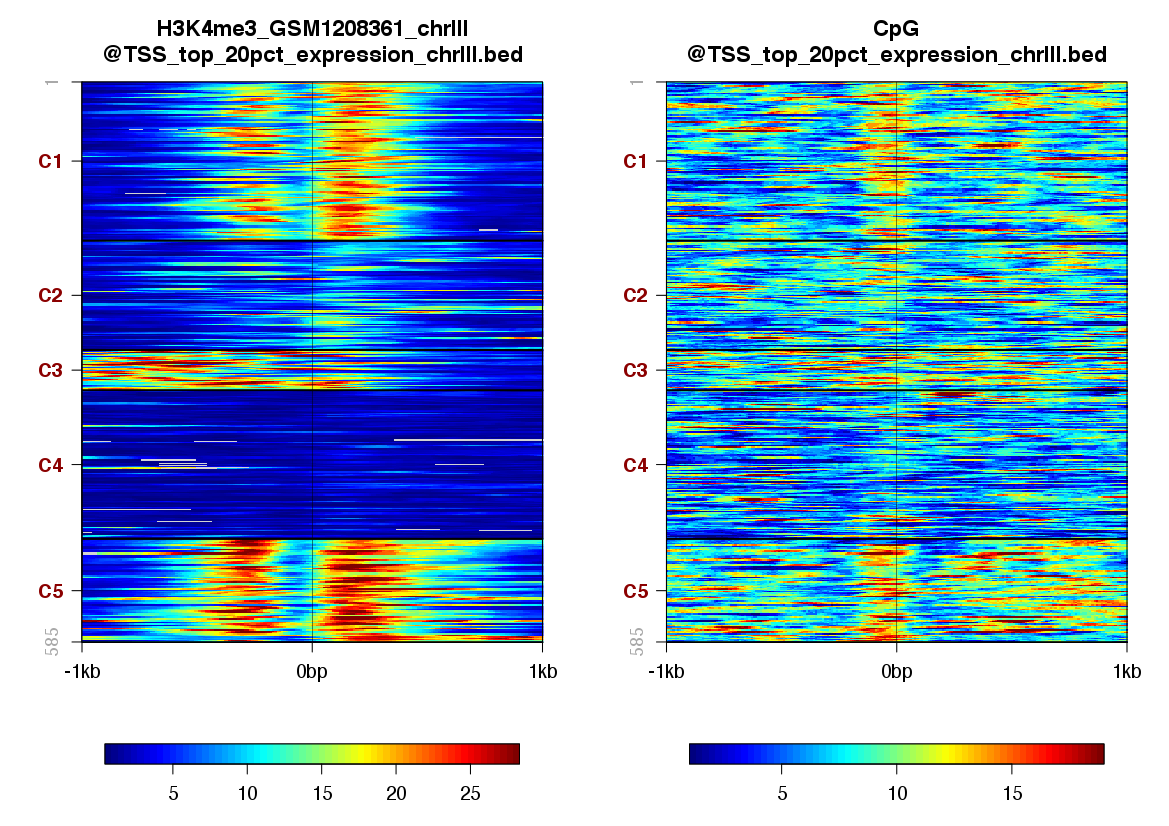

Supplement: Supplementary file 3 [file 2763FileS3.zip › mcclintock_analysis_code/seqplots-master/inst/seqplots/www/help/img/01_QuickStart/01_QuickStart_12.png]

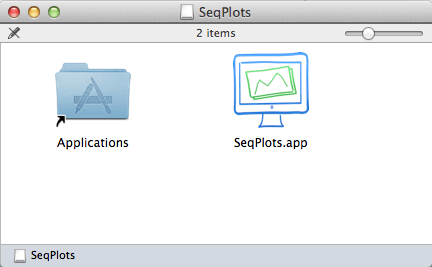

Supplement: Supplementary file 3 [file 2763FileS3.zip › mcclintock_analysis_code/seqplots-master/inst/seqplots/www/help/img/03_01.png]

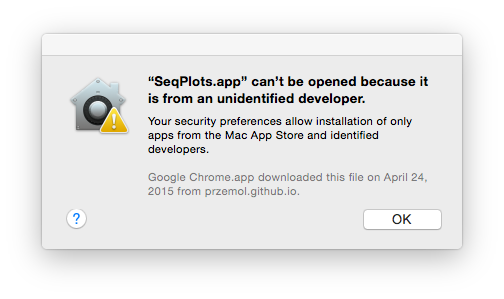

Supplement: Supplementary file 3 [file 2763FileS3.zip › mcclintock_analysis_code/seqplots-master/inst/seqplots/www/help/img/03_01a.png]

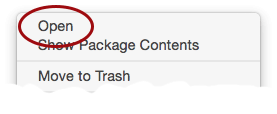

Supplement: Supplementary file 3 [file 2763FileS3.zip › mcclintock_analysis_code/seqplots-master/inst/seqplots/www/help/img/03_01b.png]

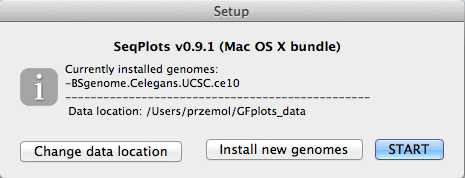

Supplement: Supplementary file 3 [file 2763FileS3.zip › mcclintock_analysis_code/seqplots-master/inst/seqplots/www/help/img/03_02.png]

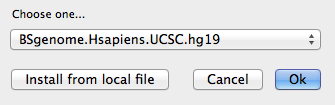

Supplement: Supplementary file 3 [file 2763FileS3.zip › mcclintock_analysis_code/seqplots-master/inst/seqplots/www/help/img/03_03.png]

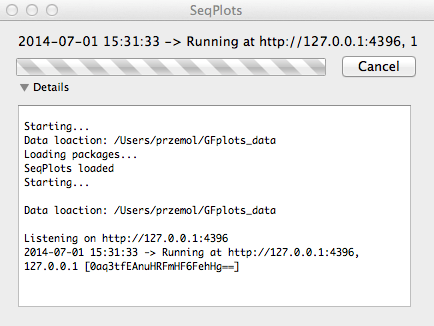

Supplement: Supplementary file 3 [file 2763FileS3.zip › mcclintock_analysis_code/seqplots-master/inst/seqplots/www/help/img/03_04.png]

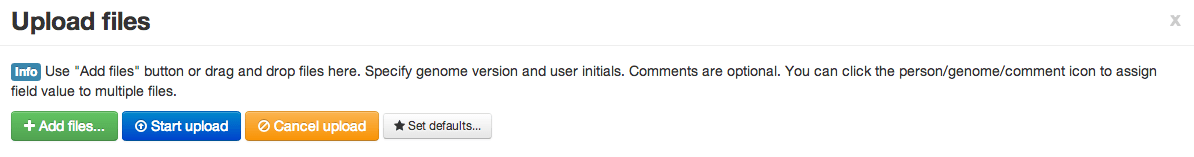

Supplement: Supplementary file 3 [file 2763FileS3.zip › mcclintock_analysis_code/seqplots-master/inst/seqplots/www/help/img/05_01.png]

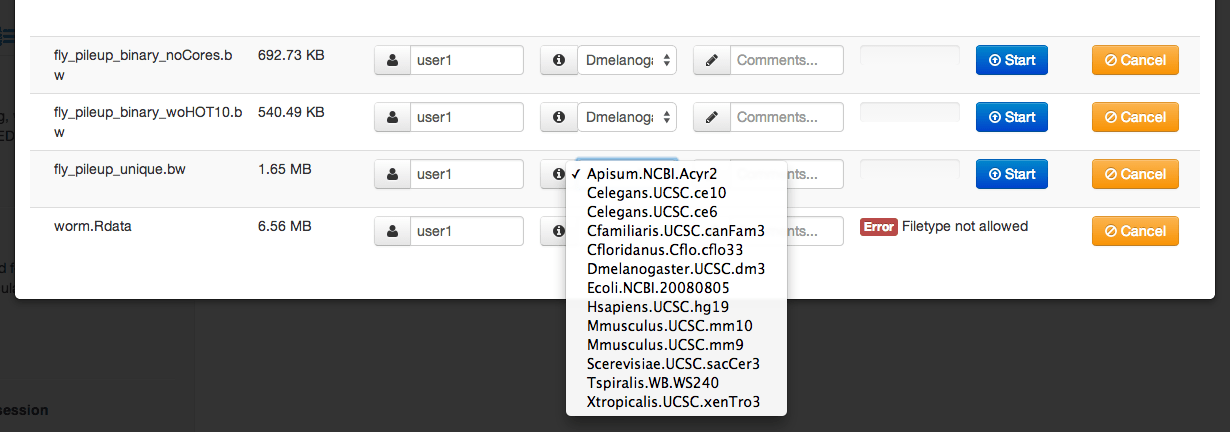

Supplement: Supplementary file 3 [file 2763FileS3.zip › mcclintock_analysis_code/seqplots-master/inst/seqplots/www/help/img/05_02.png]

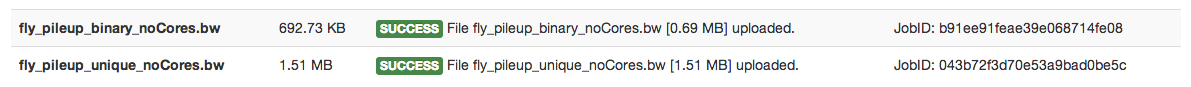

Supplement: Supplementary file 3 [file 2763FileS3.zip › mcclintock_analysis_code/seqplots-master/inst/seqplots/www/help/img/05_03.png]

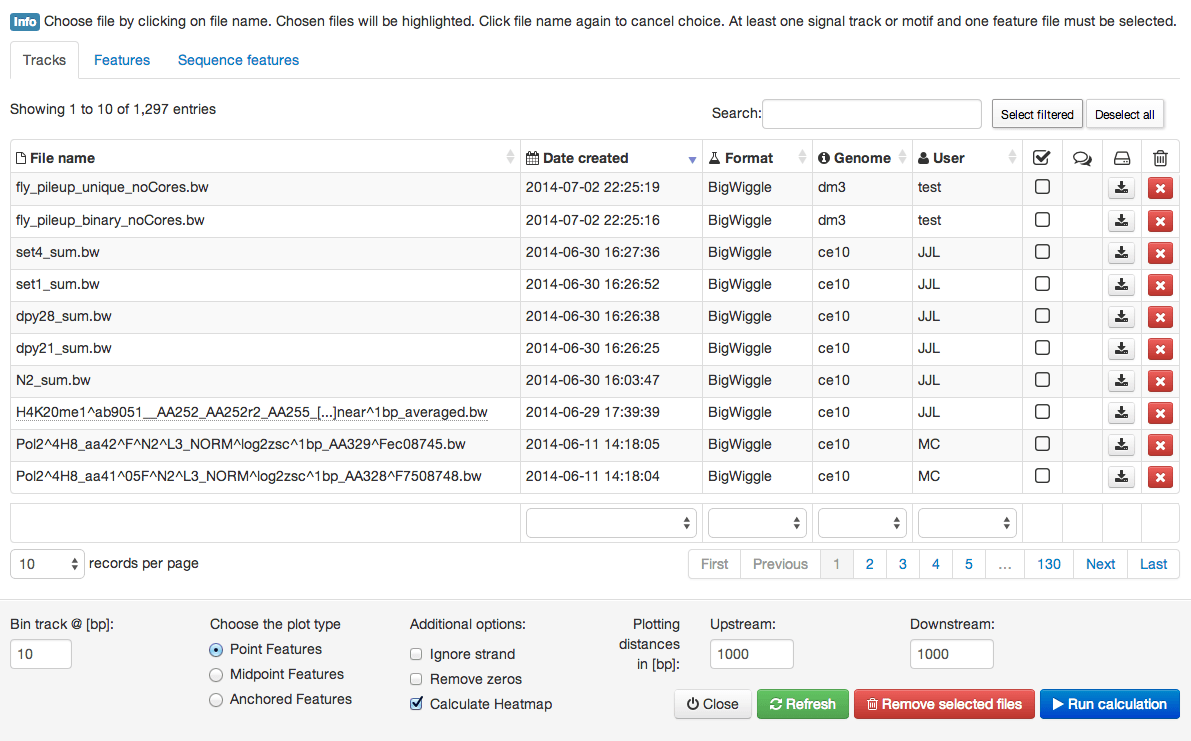

Supplement: Supplementary file 3 [file 2763FileS3.zip › mcclintock_analysis_code/seqplots-master/inst/seqplots/www/help/img/05_04.png]

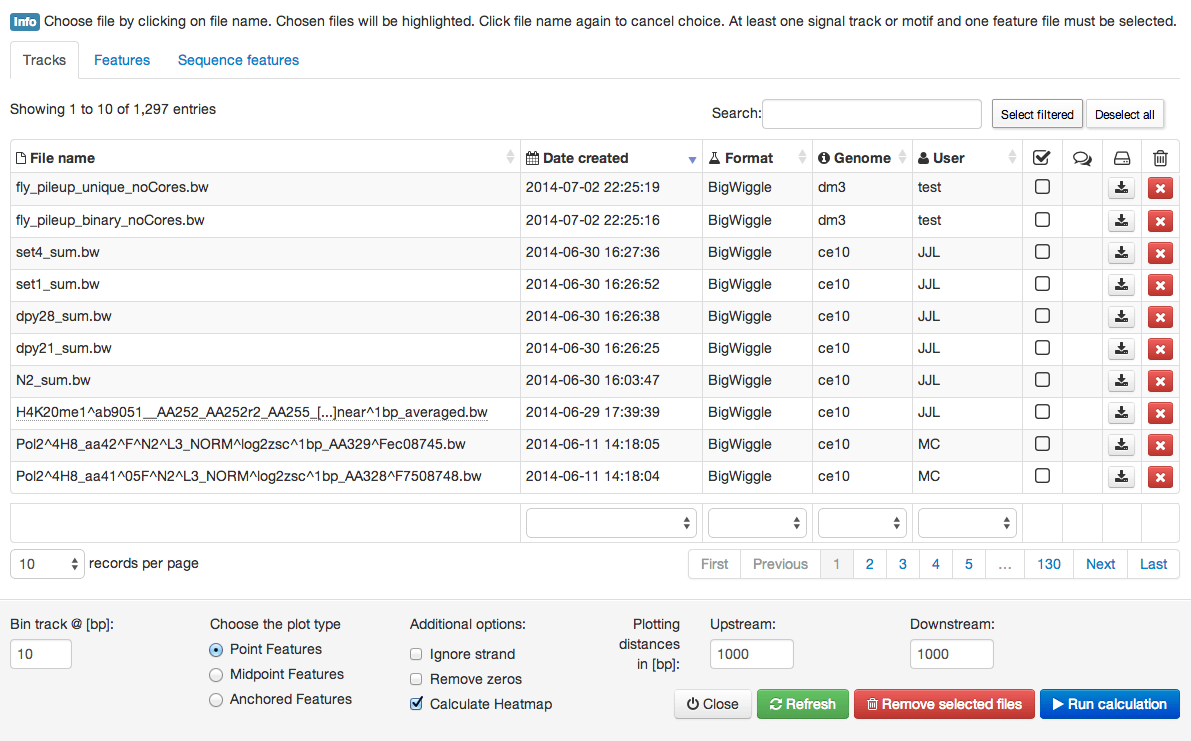

Supplement: Supplementary file 3 [file 2763FileS3.zip › mcclintock_analysis_code/seqplots-master/inst/seqplots/www/help/img/06/06_01.png]

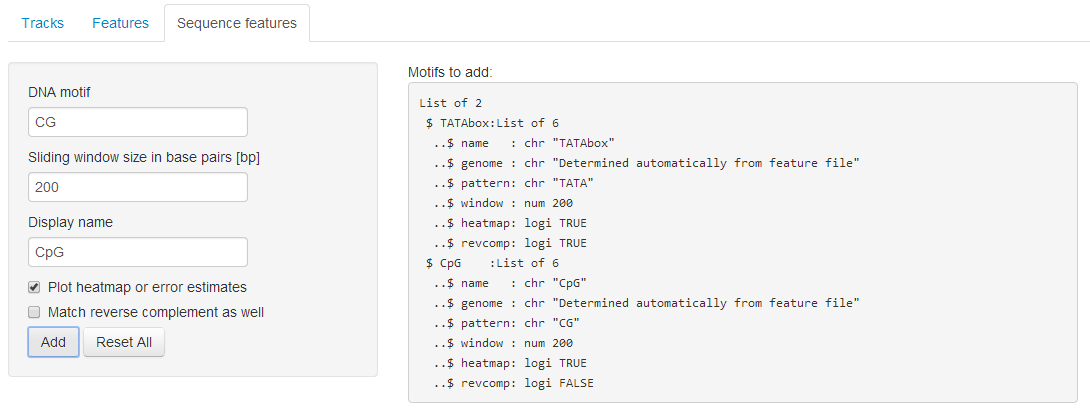

Supplement: Supplementary file 3 [file 2763FileS3.zip › mcclintock_analysis_code/seqplots-master/inst/seqplots/www/help/img/06/06_02.png]

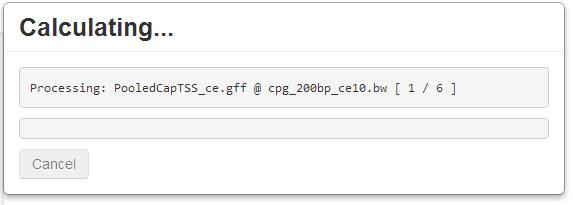

Supplement: Supplementary file 3 [file 2763FileS3.zip › mcclintock_analysis_code/seqplots-master/inst/seqplots/www/help/img/06/06_03.png]

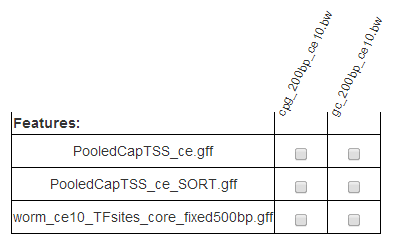

Supplement: Supplementary file 3 [file 2763FileS3.zip › mcclintock_analysis_code/seqplots-master/inst/seqplots/www/help/img/06/06_04.png]

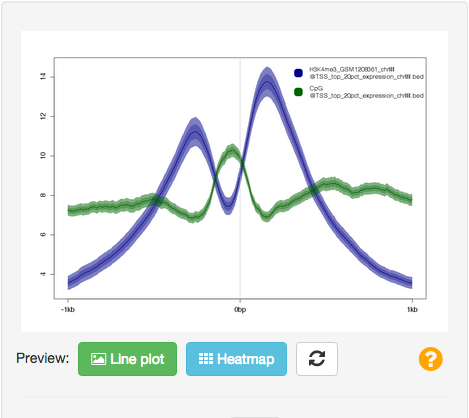

Supplement: Supplementary file 3 [file 2763FileS3.zip › mcclintock_analysis_code/seqplots-master/inst/seqplots/www/help/img/07_01.png]

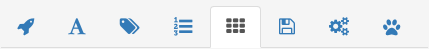

Supplement: Supplementary file 3 [file 2763FileS3.zip › mcclintock_analysis_code/seqplots-master/inst/seqplots/www/help/img/07_02.png]

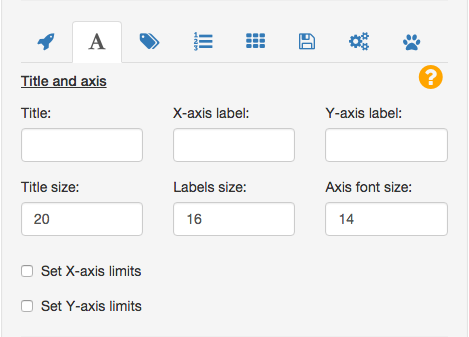

Supplement: Supplementary file 3 [file 2763FileS3.zip › mcclintock_analysis_code/seqplots-master/inst/seqplots/www/help/img/07_03.png]

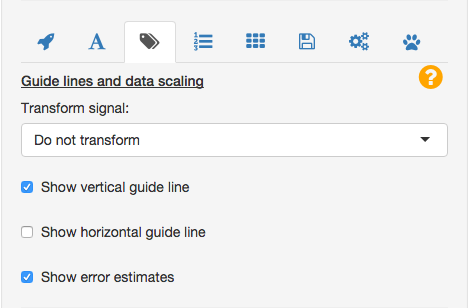

Supplement: Supplementary file 3 [file 2763FileS3.zip › mcclintock_analysis_code/seqplots-master/inst/seqplots/www/help/img/07_04.png]

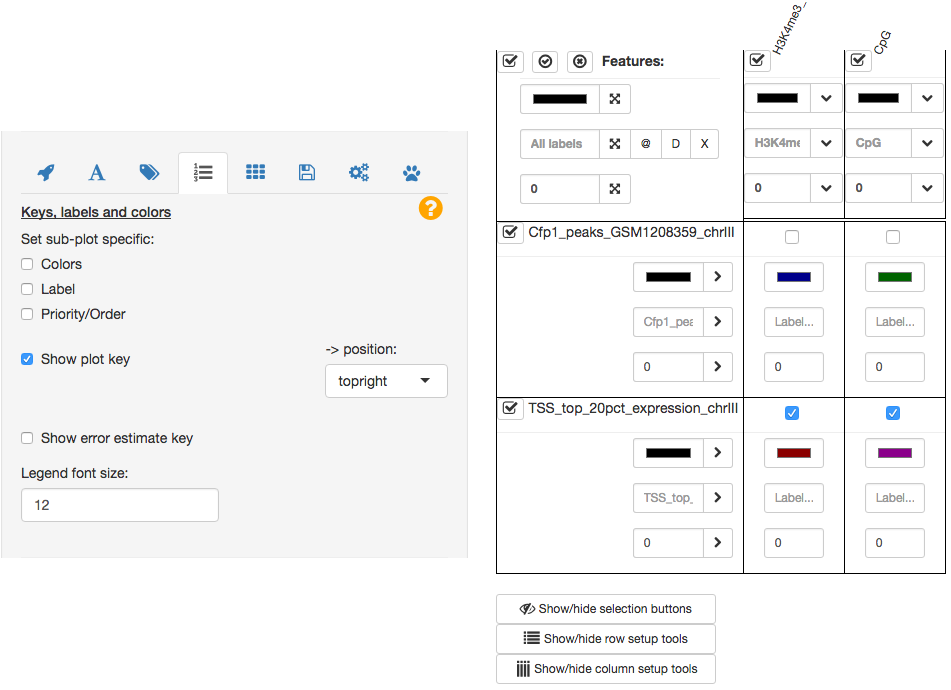

Supplement: Supplementary file 3 [file 2763FileS3.zip › mcclintock_analysis_code/seqplots-master/inst/seqplots/www/help/img/07_05_06.png]

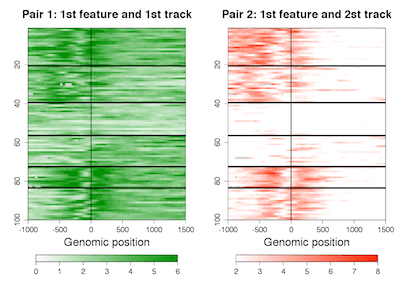

Supplement: Supplementary file 3 [file 2763FileS3.zip › mcclintock_analysis_code/seqplots-master/inst/seqplots/www/help/img/08_01.png]

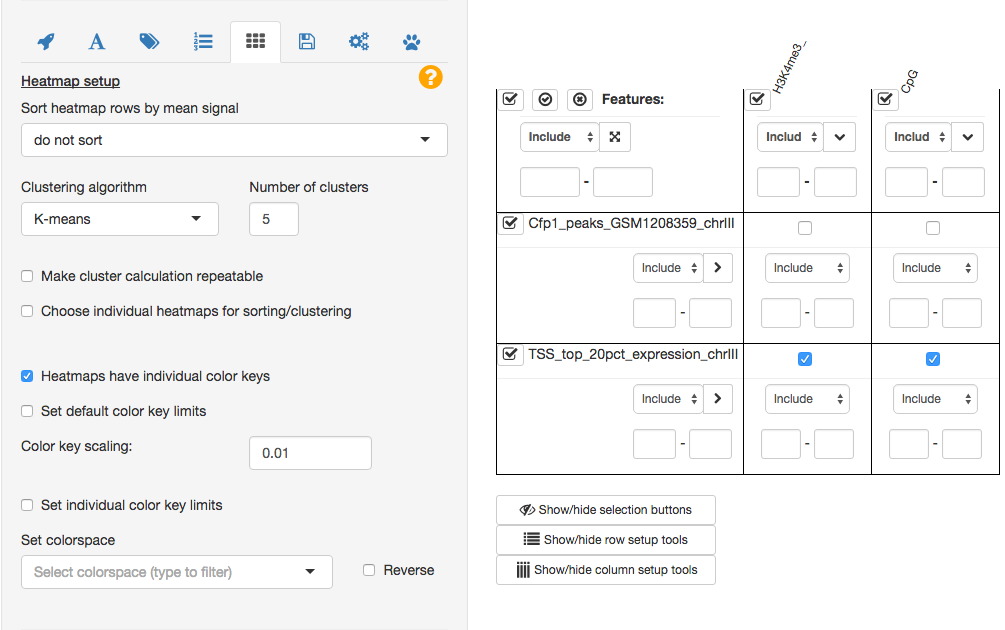

Supplement: Supplementary file 3 [file 2763FileS3.zip › mcclintock_analysis_code/seqplots-master/inst/seqplots/www/help/img/08_02.png]

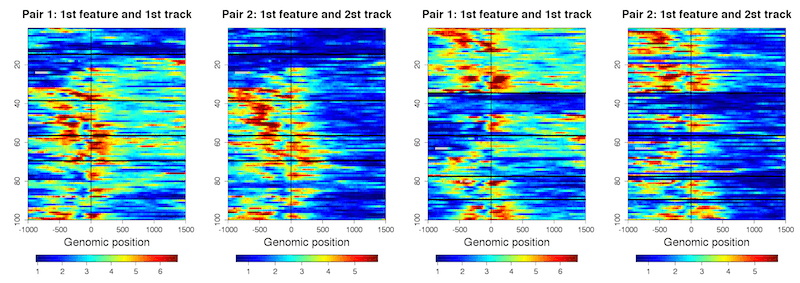

Supplement: Supplementary file 3 [file 2763FileS3.zip › mcclintock_analysis_code/seqplots-master/inst/seqplots/www/help/img/08_03.png]

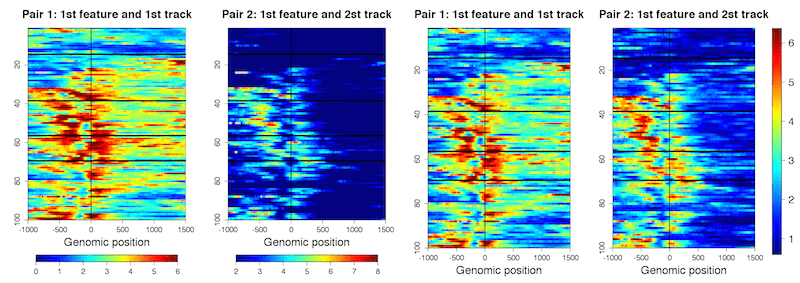

Supplement: Supplementary file 3 [file 2763FileS3.zip › mcclintock_analysis_code/seqplots-master/inst/seqplots/www/help/img/08_04.png]

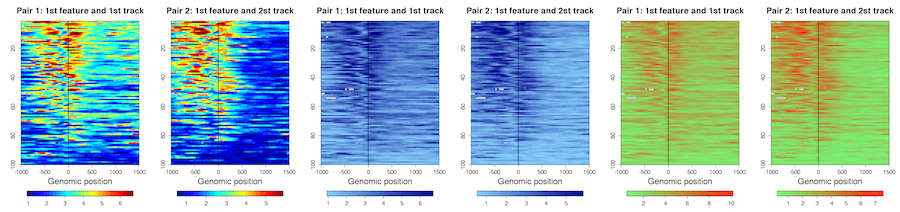

Supplement: Supplementary file 3 [file 2763FileS3.zip › mcclintock_analysis_code/seqplots-master/inst/seqplots/www/help/img/08_05.png]

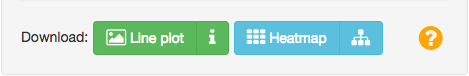

Supplement: Supplementary file 3 [file 2763FileS3.zip › mcclintock_analysis_code/seqplots-master/inst/seqplots/www/help/img/09_01.png]

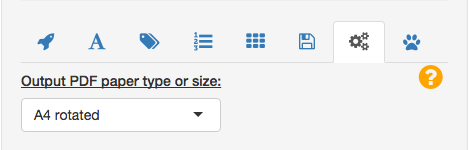

Supplement: Supplementary file 3 [file 2763FileS3.zip › mcclintock_analysis_code/seqplots-master/inst/seqplots/www/help/img/09_02.png]

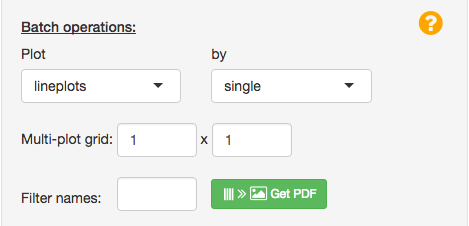

Supplement: Supplementary file 3 [file 2763FileS3.zip › mcclintock_analysis_code/seqplots-master/inst/seqplots/www/help/img/09_03.png]

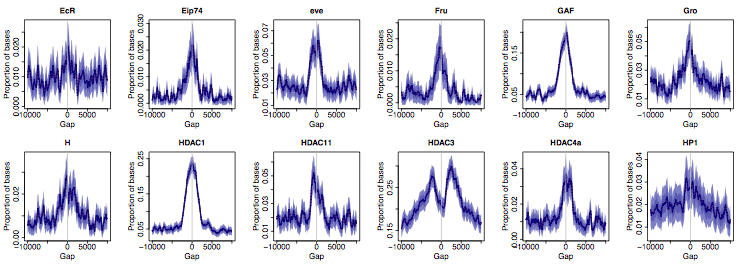

Supplement: Supplementary file 3 [file 2763FileS3.zip › mcclintock_analysis_code/seqplots-master/inst/seqplots/www/help/img/09_04.png]

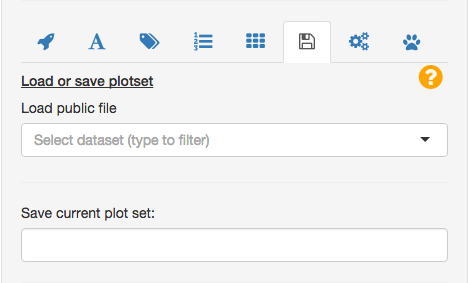

Supplement: Supplementary file 3 [file 2763FileS3.zip › mcclintock_analysis_code/seqplots-master/inst/seqplots/www/help/img/10_00.png]

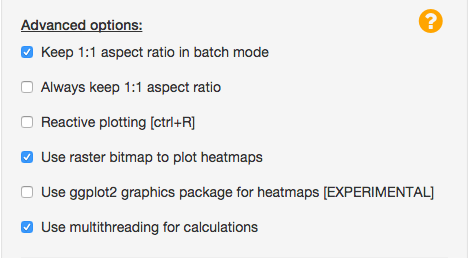

Supplement: Supplementary file 3 [file 2763FileS3.zip › mcclintock_analysis_code/seqplots-master/inst/seqplots/www/help/img/10_01.png]

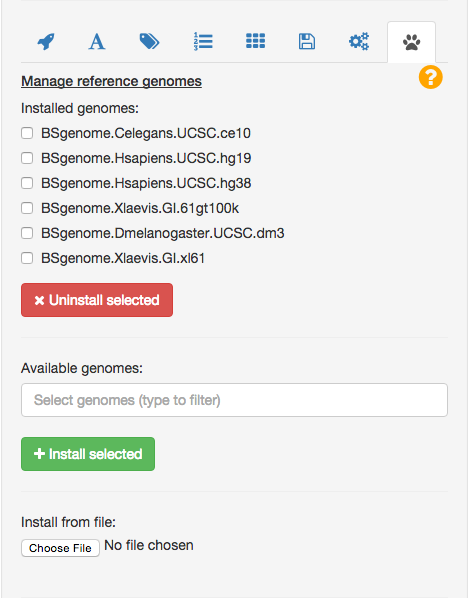

Supplement: Supplementary file 3 [file 2763FileS3.zip › mcclintock_analysis_code/seqplots-master/inst/seqplots/www/help/img/11_01.png]

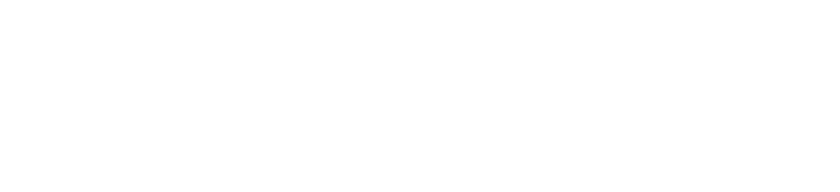

Supplement: Supplementary file 3 [file 2763FileS3.zip › mcclintock_analysis_code/seqplots-master/inst/seqplots/www/help/img/ico_full.png]

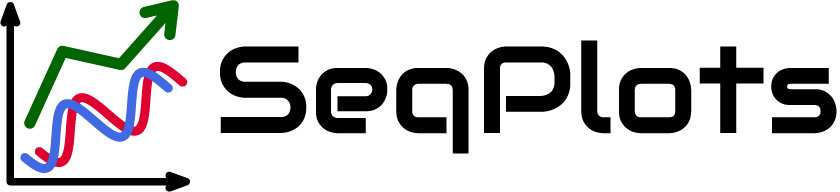

Supplement: Supplementary file 3 [file 2763FileS3.zip › mcclintock_analysis_code/seqplots-master/inst/seqplots/www/help/img/ico_full_color.png]

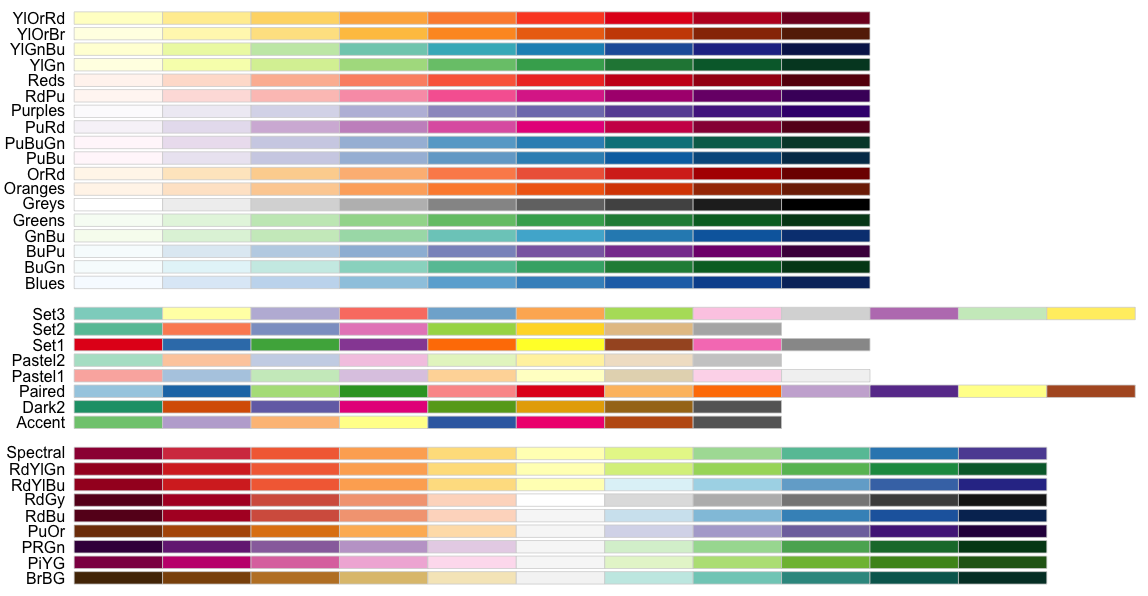

Supplement: Supplementary file 3 [file 2763FileS3.zip › mcclintock_analysis_code/seqplots-master/inst/seqplots/www/help/img/palettes.png]

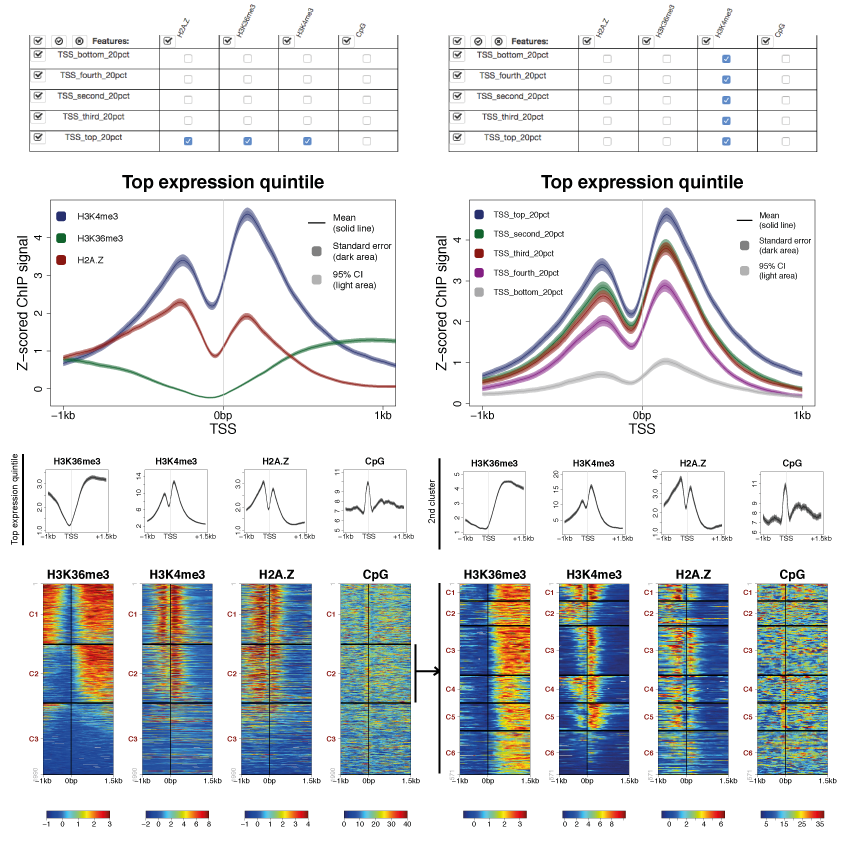

Supplement: Supplementary file 3 [file 2763FileS3.zip › mcclintock_analysis_code/seqplots-master/inst/seqplots/www/help/img/SeqPlots_fig1_web.png]

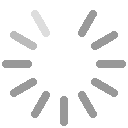

Supplement: Supplementary file 3 [file 2763FileS3.zip › mcclintock_analysis_code/seqplots-master/inst/seqplots/www/upload/img/loading.gif]

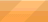

Supplement: Supplementary file 3 [file 2763FileS3.zip › mcclintock_analysis_code/seqplots-master/inst/seqplots/www/upload/img/progressbar.gif]
